# Interactions of imidazole with water molecules

**DOI:** 10.1007/s00894-025-06515-4

**Published:** 2025-09-25

**Authors:** Alhadji Malloum, Jeanet Conradie

**Affiliations:** 1 Department of Physics, Faculty of Science, University of Maroua, PO BOX 46, Maroua, Cameroon; 2 Department of Chemistry, University of the Free State, PO BOX 339, Bloemfontein, 9300 South Africa

**Keywords:** Imidazole, Water clusters, Non-covalent interactions, DFT benchmarking

## Abstract

**Context:**

Understanding the interactions of imidazole and water molecules is essential for several chemical and biological activities. Literature mining shows that investigations of hydrated imidazole are rare. In this work, interactions between imidazole and explicit water molecules are investigated. The structures of the complexes formed by imidazole and water molecules are used to estimate imidazole’s hydration enthalpy and free energy. QTAIM investigation shows that imidazole accepts two strong hydrogen bondings while donating one. In addition, it also interacts with water molecules through weaker bonding interactions. The results reported in this work reproduce previous experimental observations and molecular dynamics simulations.

**Methods:**

The investigation started by generating initial configurations through global optimizations using classical potential energy. Then, a suitable functional of density functional theory (DFT) is selected between 20 functionals, including dispersion corrections, by benchmarking to the DLPNO-CCSD(T1)/CBS. The M06L-D3 functional is found to be the most accurate. The structures of the imidazole-water clusters, $\text {IMZ}(\text {H}_2\text {O})_n$, $n=1-12,\;64$, are then optimized at the M06L-D3/def2-TZVPP level of theory. Hydration free energy and enthalpy are estimated using the cluster continuum solvation model. Calculations are performed using Gaussian 16 and ORCA suite of programs. Quantum theory of atoms in molecules (QTAIM) is performed using the AIMAll program.

**Supplementary Information:**

The online version contains supplementary material available at 10.1007/s00894-025-06515-4.

## Introduction

Imidazole (IMZ) is a five-membered heterocyclic and aromatic molecule containing two nonadjacent nitrogen atoms. Imidazole is soluble in water and is found in several natural products, such as alkaloids. Imidazole is used in several chemical and industrial applications. It can be found in compounds that have several pharmaceutical applications. Due to its importance and solubility, understanding micro-hydration is essential for theoretical studies of imidazole in water. Therefore, we investigate the micro-hydration of imidazole with one to twelve explicit water molecules, $\text {IMZ}(\text {H}_2\text {O})_n$, $n=1-12$, as well as $n=64$.

The interaction of imidazole with water molecules has been studied by a few authors in the literature. Most investigations have been performed using molecular dynamics (MD) simulations. Duboué-Dijon et al. [10] characterized the number of hydrogen bonds established between imidazole and water molecules in the hydrated imidazole. The authors used neutron scattering, classical, and *ab-initio* molecular dynamics simulations. All the methodologies used by the authors converged to the same conclusion: imidazole donates one hydrogen bond (through the NH donor) and accepts two hydrogen bondings (through the N3’s lone pair). They have further observed that the two donating water molecules are out of the imidazole plane [10]. Later, Pagliai et al. [39] have also investigated the hydration of imidazole using classical molecular dynamics and Car-Parrinello molecular dynamics (CPMD) simulations. It has been found that the study of hydrogen bondings in classical MD reproduces the results of CPMD. Furthermore, the authors found that the hydrogen bonding interactions between imidazole and water molecules are in perfect agreement with the results of Duboué-Dijon et al. [10] (presented above). The position of water molecules interacting with the N3 atom is out of the imidazole plane [39]. Recently, a similar investigation was reported by Al-Madhagi and coworkers [2] on aqueous imidazole solutions using neutron diffraction and x-ray scattering. They concluded that the most probable structure is the hydrated imidazole in aqueous imidazole solution [2]. In perfect agreement with Duboué-Dijon et al. [10] and Pagliai et al. [39], the authors found that imidazole donates one hydrogen bond through N1 while accepting two hydrogen bond through N3, with the donating water molecules positioning out of the imidazole plane [2].

In addition to molecular dynamics simulations and experimental study of the hydrated imidazole, the interaction of imidazole with one water molecule is investigated at HF/6-31 G(d) and MP2/6-31 G(d) levels of theory [3, 36]. Two structures were located: imidazole acts as a hydrogen bond donor and acceptor. The energetic difference between these two structures is 0.13 kcal/mol [36]. As reported above, this study has been limited to one explicit water molecule, which does not allow the proper interpretation of experimental observation and MD simulations. Recently, hydration of imidazole and 2-aminoimidazole has been reported at the B3LYP/cc-pVTZ level of theory by Kerkeni and Bacchus-Montabonel [23]. The incremental addition of water molecules from one to six has been assessed. The authors found that by increasing the number of explicit water molecules, the latter establish hydrogen bondings with imidazole to form an “umbrella” chain around imidazole [23]. Besides the limited number of explicit water molecules, the DFT functional B3LYP used by the authors does not consider dispersion corrections, which are essential in imidazole-water clusters. Recently, Melli, Barone, and Puzzarini [35] has investigated the interaction of Imidazole and one water molecule using a semiexperimental approach. This approach has confirmed the existence of two isomers of imidazole-water complexe.

Few authors have also reported micro-hydration of molecules containing imidazole. Sharma, Rao, and Sastry [50] investigated the hydration of ions binding to imidazole and methylimidazole. All the assessed ions are cations, and they bind to imidazole N3 atoms. Incremental adding of water molecules shows that they prefer mainly to bind to the cation and the N1H donor [50]. Deprotonation and binding of imidazole on the gold surface in aqueous solution have been investigated [20]. Bhattacherjee and Wategaonkar investigate the hydration of imidazole derivatives with one explicit water molecule [8].

Exploration of the literature shows that the hydration of imidazole has received negligible attention despite its evident importance. Apart from molecular dynamics simulations, investigation of explicit hydration of imidazole using *ab-initio* or density functional theory (DFT) is rare. Even for the reported investigations, the imidazole-water clusters’ potential energy surfaces (PESs) have not been thoroughly explored. This limitation can considerably affect the accuracy of the located structures. Therefore, we thoroughly explored the PESs of the micro-hydrated imidazole (or imidazole-water clusters), $\text {IMZ}(\text {H}_2\text {O})_n$, $n=1-12,\;64$. We benchmark several DFT-D3 functionals to the DLPNO-CCSD(T1)/CBS to select the most accurate one for the investigation of $\text {IMZ}(\text {H}_2\text {O})_n$ complexes. Initial configurations are generated using classical molecular dynamics implemented in ABCluster. Quantum theory of atoms in molecules (QTAIM) analysis is performed to understand the non-covalent interactions in imidazole-water clusters.

## Methodology

The investigation starts by generating the possible structures of the micro-hydrated imidazole $\text {IMZ}(\text {H}_2\text {O})_n$, $n=1-12$ (see the “Sampling initial configurations” section). The generated structures are used in calculating the hydration enthalpy and free energy of imidazole. The calculation procedure is presented in the “Hydration enthalpy and free energy” section. Before selecting the suitable DFT functionals for the investigation, we benchmark twenty DFT functionals, including dispersion corrections. The benchmarking procedure and the computational details are presented in the “Computational details” section.

### Sampling initial configurations

For a given cluster size *n*, there are several possible configurations of $\text {IMZ}(\text {H}_2\text {O})_n$ cluster. Thus, it is important to properly identify these configurations and to consider all those that have meaningful contributions to the cluster’s population. One of the possible approaches to generate the possible configurations using the affordable classical molecular dynamics is the bee colony algorithm implemented in the ABCluster code [60, 61]. ABCluster tries to reproduce how bees identify the best nectar in nature and applies it to identify possible configurations of molecular clusters. The classical energy used in the program considers only electrostatic and Lenard-Jones interactions. The potential energy is calculated using the CHARMM force field [54]. The generated structures have been fully optimized using a DFT functional carefully selected after benchmarking twenty DFT functionals. The selection is presented in the “DFT benchmarking” section.

**Fig. 1:**
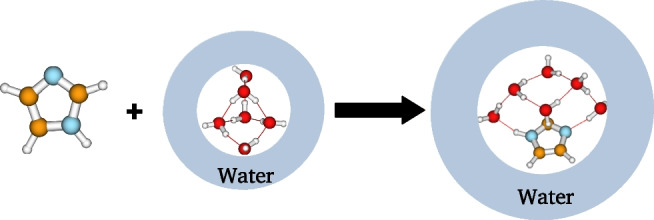
Schematic representation of the hydration free energy and enthalpy calculation used in this work. We use the example of six explicit water molecules

### Hydration enthalpy and free energy

As an application of the structures of the $\text {IMZ}(\text {H}_2\text {O})_n$ clusters, we calculated the hydration free energy and enthalpy of imidazole at several ranges of temperatures. The hydration free energy and enthalpy of imidazole are calculated in this work using the cluster continuum solvation model. The schematic representation of the model is given in Fig. 1. The idea of the model is to describe quantum mechanically few explicit water molecules around imidazole while the remaining solvent molecules are considered a dielectric continuum medium. Thus, we apply the hybrid solvation model, combining explicit and implicit solvation. Within the cluster continuum solvation model, the hydration free energy and enthalpy are the free energy and enthalpy of the following reaction:

1 $$\begin{aligned} \left[ \text {IMZ}\right] _{gas} + \left[ (\text {H}_2\text {O})_n\right] _{water}\longrightarrow \left[ \text {IMZ}(\text {H}_2\text {O})_n\right] _{water} \end{aligned}$$

Thus, the hydration free energy and enthalpy of imidazole can then be calculated using the following equations:

2 $$\begin{aligned} \Delta H_{solv}(\text {IMZ})_n&=H\left[ \text {IMZ}(\text {H}_2\text {O})_n\right] _{water}\nonumber \\&-H\left[ (\text {H}_2\text {O})_n\right] _{water}-H\left[ \text {IMZ}\right] _{gas}, \end{aligned}$$

3 $$\begin{aligned} \Delta G_{solv}(\text {IMZ})_n&=G\left[ \text {IMZ}(\text {H}_2\text {O})_n\right] _{water}\nonumber \\&-G\left[ (\text {H}_2\text {O})_n\right] _{water}-G\left[ \text {IMZ}\right] _{gas}, \end{aligned}$$

where *H*[*X*] and *G*[*X*] are the enthalpy and free energy of *X*, respectively. As can be seen in Eqs. 2 and 3, the hydration enthalpy and free energy are functions of the number of explicit water molecules *n*. The obtained function of *n* is expected to converge with increasing water molecule *n*. Consequently, the hydration enthalpy and free energy to be retained is the one obtained when *n* tends to infinity. It is important to note that the enthalpy $H\left[ \text {IMZ}(\text {H}_2\text {O})_n\right] _{water}]$ and $H\left[ (\text {H}_2\text {O})_n\right] _{water}$, in Eqs. 2 and 3, are calculated as Boltzmann weighted average of the enthalpy and free energy of the located structures of the $n-$mer. Same for the free energy. The structures of $\left[ (\text {H}_2\text {O})_n\right] _{water}$ and $\left[ \text {IMZ}(\text {H}_2\text {O})_n\right] _{water}$ are optimized in the solvent phase through the self-consistent reaction field (SCRF) using the solvation model based on density (SMD). Thus, the located structures are optimized in the implicit solvent phase.

### Computational details

**Fig. 2:**
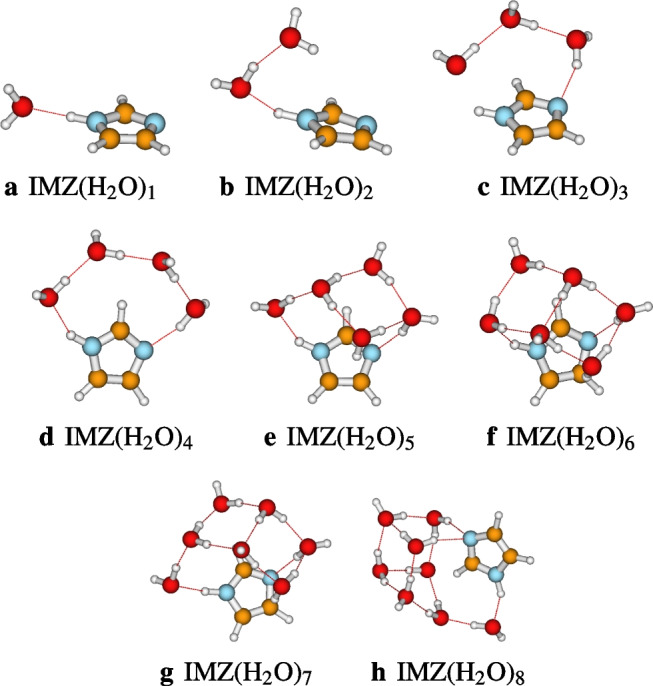
Structures of the hydrated imidazole used to benchmark DFT functionals. The structures reported here are optimized at the MP2/def2-TZVPP level of theory

To identify the most suitable functional for the study of the micro-hydration of imidazole, we perform a benchmark of some selected DFT functionals. For benchmarking the DFT functionals, we have selected twenty DFT functionals that include dispersion corrections [14, 16]. The inclusion of empirical dispersion is essential to expect accurate descriptions from DFT functionals, considering the dispersive nature of the hydrated imidazole structures. The functionals include APFD [4], B3LYP-D3 [7, 24], B3PW91-D3 [7, 42], B97D3 [15], BLYP-D3 [6, 24] BP86-D3 [6, 41] BPBE-D3 [6, 43] CAM-B3LYP-D3 [56], M052X-D3 [65], M05-D3 [64], M062X-D3 [63], M06HF-D3 [63], M06L-D3 [63], M06-D3 [63], MN15 [57, 58], PBE1PBE-D3 [1], PBEPBE-D3 [43], PW6B95D3 [62], TPSSTPSS-D3 [53], and $\omega$B97XD [9]. The functionals were associated with the def2-TZVPP basis set for the calculations. For each of the functional, the obtained lowest energy IMZ$(\text {H}_2\text {O})_n$ as shown in Fig. 2 have been optimized in addition to the structures of imidazole and the water molecules. The optimizations have been performed using the Gaussian 16 suite of code [11]. The *tight* option and *ultrafine* grid have been used for the accuracy of the calculations. Calculations have been performed in the gas phase. Corrections of basis set superposition error (BSSE) are not applied in this work. For a triple-zeta basis set, BSSE corrections are expected to be less considerable on the calculated binding energies and hydration free energies [30].

The selected DFT functionals have been benchmarked to the DLPNO-CCSD(T1)/CBS level of theory. DLPNO-CCSD(T1) [17] is the improved and more accurate implementation of the initial DLPNO-CCSD(T0) [46, 49]. Calculations at this level of theory have been performed using the ORCA computational Chemistry code (version 5.0.3) [37]. For the accuracy of the calculations, we used *tightpno* and *tightscf* options. The *AutoAux* generation procedure has been used to generate the auxiliary basis sets automatically [52]. For the complete basis set (CBS) extrapolations, we employed the two-point energy strategies, which request two single-point energy calculations (aug-cc-pVTZ and aug-cc-pVQZ for the present work [22]). In the two-point energy strategy, one needs to calculate two single-point energies using aug-cc-pVXZ basis sets (where X=D, T, Q, 5,... corresponding to the angular moment $L=2$, 3, 4, 5,..., respectively). For $L=M$ and $L=N$ ($M>N$), the SCF (self-consistent field) part of the energy is extrapolated using [66]

4 $$\begin{aligned} E_{SCF}(\infty )=\dfrac{E_{SCF}(M)\exp \left( -\alpha \sqrt{N}\right) -E_{SCF}(N)\exp \left( -\alpha \sqrt{M}\right) }{\exp \left( -\alpha \sqrt{N}\right) -\exp \left( -\alpha \sqrt{M}\right) }, \end{aligned}$$

while the correlation part is extrapolated by [18]

5 $$\begin{aligned} E_{corr}(\infty )=\dfrac{E_{corr}(N)N^{\beta }-E_{corr}(M)M^{\beta }}{N^{\beta }-M^{\beta }}, \end{aligned}$$

with $\alpha =5.79$ and $\beta =3.05$ for $M=4$ and $N=3$ used in this work [38].

The non-covalent bondings are studied using the quantum theory of atoms in molecules (QTAIM) analysis. QTAIM analysis is performed using the AIMAll program [21]. QTAIM analysis is performed based on the electron density computed at the M06L-D3/def2-TZVPP level of theory.

## Results and discussions

Before performing the current investigation, we identify the most suitable DFT functionals by benchmarking twenty DFT functionals. The benchmark binding energies are calculated at the DLPNO-CCSD(T1)/CBS level of theory. The benchmarking study is presented in the “DFT benchmarking” section. The identified functional is then used to optimize the structures of the IMZ(H$_2$O)$_n$ clusters for $n=1-12$. The structures and their relative energies are presented in the “[Sec Sec8]” section. Non-covalent interactions of the identified structures are performed using the quantum theory of atoms in molecules (QTAIM) analysis (see the “[Sec Sec9]” section). In addition, the relative population of the clusters is presented and discussed in the “Relative population and clustering incremental energies” section. Besides, clustering incremental energies (electronic energies, free energies, and enthalpies) are also reported in the “Relative population and clustering incremental energies” section. Finally, the hydration free energy and enthalpy are presented in the “Solvation enthalpy and free energy of imidazole in water” section.

**Fig. 3:**
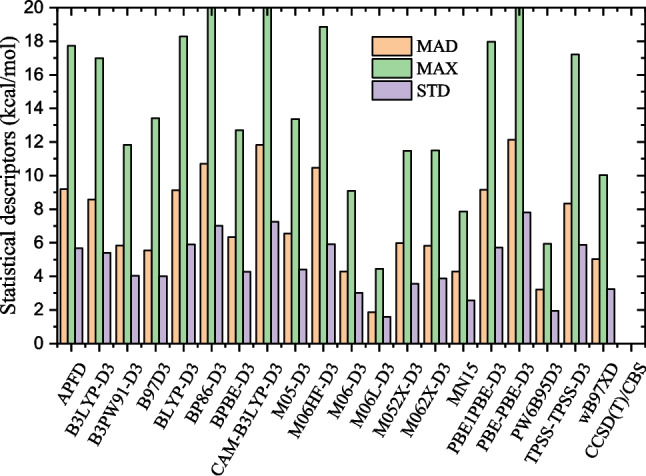
Statistical descriptors of the twenty DFT functionals assessed in this work. MAD is mean absolute deviation, MAX is maximum absolute deviation, and STD is standard deviation in reference to the DLPNO-CCSD(T)/CBS level of theory. CCSD(T)/CBS represents the DLPNO-CCSD(T)/CBS level of theory. The M06L-D3 functional has the smallest mean absolute deviation

**Table 1:** Binding energies of the hydrated imidazole calculated using twenty DFT functionals associated with the def2TZVPP basis set

n	M06-D3	M06L-D3	MN15	PW6B95D3	$\omega$B97XD	CCSD(T)/CBS
1	$-$6.1	$-$5.8	$-$6.3	$-$6.4	$-$6.4	$-$6.0
2	$-$16.6	$-$15.6	$-$17.4	$-$16.2	$-$16.8	$-$15.1
3	$-$30.0	$-$28.5	$-$30.7	$-$29.8	$-$30.5	$-$28.0
4	$-$42.3	$-$40.4	$-$42.2	$-$42.3	$-$43.8	$-$39.9
5	$-$54.9	$-$52.2	$-$55.2	$-$53.6	$-$55.6	$-$49.8
6	$-$68.2	$-$64.5	$-$67.8	$-$65.8	$-$68.5	$-$60.9
7	$-$79.2	$-$75.5	$-$79.1	$-$77.9	$-$80.9	$-$72.5
8	$-$93.2	$-$88.5	$-$91.9	$-$90.0	$-$94.1	$-$84.1
MAD	4.3	1.9	4.3	3.2	5.0	0.0
MAX	9.1	4.4	7.9	5.9	10.0	0.0
STD	3.0	1.6	2.6	2.0	3.2	0.0

Values of functionals that perform best are shown, see Table S1 for a full list. CCSD(T)/CBS stands for DLPNO-CCSD(T1)/CBS, MAD is mean absolute deviation, MAX is the maximum deviation, and STD is the standard deviation, calculated in reference to the DLPNO-CCSD(T1)/CBS level of theory

### DFT benchmarking

We use the most stable structures generated by ABCluster to perform the benchmark. These structures, as optimized at the MP2/def2-TZVPP level of theory, are provided in Fig. 2. Each of the structures of Fig. 2 has been optimized at the corresponding DFT functional to calculate the binding energies. Binding energies, $\Delta E_n$, are calculated using Eq. 6:

6 $$\begin{aligned} \Delta E_n=E_n-E(IMZ)-nE(\text {H}_2\text {O}) \end{aligned}$$

where $E_n$ is the energy of IMZ(H$_2$O)$_n$, *E*(*IMZ*) is the energy of the imidazole molecule, and $E(\text {H}_2\text {O})$ is the energy of the water molecule. The calculated binding energies are reported in the supplementary material (Table S1).

To avoid cumbersome presentation, the benchmark binding energies are reported in the supplementary material while the statistical descriptors of the functionals, including mean absolute deviation (MAD), maximum absolute deviation (MAX), and standard deviation (STD), are reported in Fig. 3. The DLPNO-CCSD(T1)/CBS binding energies, as well as the binding energies calculated using DFT functionals achieving a MAD of lower than 5.0 kcal/mol, are reported in Table 1. Among the assessed functionals, the most accurate DFT functional is M06L-D3, which has a mean absolute deviation of 1.9 kcal/mol. The second most accurate functional is the PW6B95D3 with a MAD of 3.2 kcal/mol. The M06L-D3 and the PW6B95D3 functionals have been recommended in previous works for their accuracy. The M06L-D3 functional is reported to provide satisfactory results for the energetic study in the stacking of nickel and copper chelates [26]. Furthermore, functional PW6B95D3 is the best in calculating the ammonia clusters’ binding energies [27]. Elsewhere, the worst performance is noted for the functionals PBEPBE-D3, CAM-B3LYP-D3, BP86-D3, and M06HF-D3. These functionals have achieved a mean absolute deviation higher than 10.5 kcal/mol. Larger DFT benchmarking studies on non-covalent systems have been performed in the literature [12, 34]. Mardirossian and Head-Gordon [34] performed a benchmark of several DFT functionals on the MGCDB84 database, comprising nearly 5000 data points. They have recommended the functionals $\omega$B97X-V, $\omega$B97X-D3, and $\omega$B97XD as best hybrid GGA, while $\omega$B97M-V, $\omega$M05-D, M06-2X-D3, and MN15 are recommended as best hybrid meta-GGA functionals [34]. As can be seen, the recommended functionals $\omega$B97XD, M062X-D3, and MN15 by Mardirossian and Head-Gordon [34] are outperformed by M06L-D3 regarding the specific case of the imidazole-water clusters in this work. Moreover, Grimme and co-workers [12] performed a benchmark of DFT functionals on the GMTKN55 database comprising 2462 single-point calculations. From their investigations, they recommended the functionals $\omega$B97X-V, M052X-D3, $\omega$B97X-D3, and PW6B95-D3(BJ) [12]. The M052X-D3, which is among the recommended functionals, is outperformed in the present work by M06L-D3. It appears that the M06L-D3, which performs well for the imidazole-water clusters, is not among the functionals recommended by Mardirossian and Head-Gordon [34] and Grimme and coworkers [12]. This justifies the need of performing specific benchmarking, whenever possible, as done in the present investigation, for a good description of the system under investigation.

Considering the above results, the optimizations, frequency calculations, and other properties of IMZ(H$_2$O)$_n$, $n=1-12,\; 64$ are calculated at the M06L-D3/def2-TZVPP level of theory. Furthermore, clustering incremental energies, hydration free energy, and enthalpy of imidazole are also calculated at the M06L-D3/def2-TZVPP level of theory.

### Structures of IMZ(H$_2$O)$_n$, $n=1-12$ and $n=64$

**Fig. 4:**
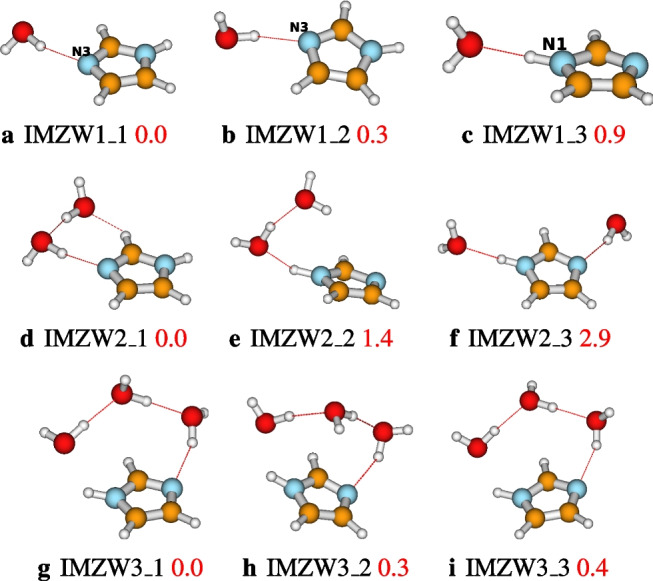
Structures of IMZ(H$_2$O)$_{1-3}$ clusters as optimized at the M06L-D3/def2-TZVPP level of theory. Relative electronic energies are provided in kcal/mol

**Fig. 5:**
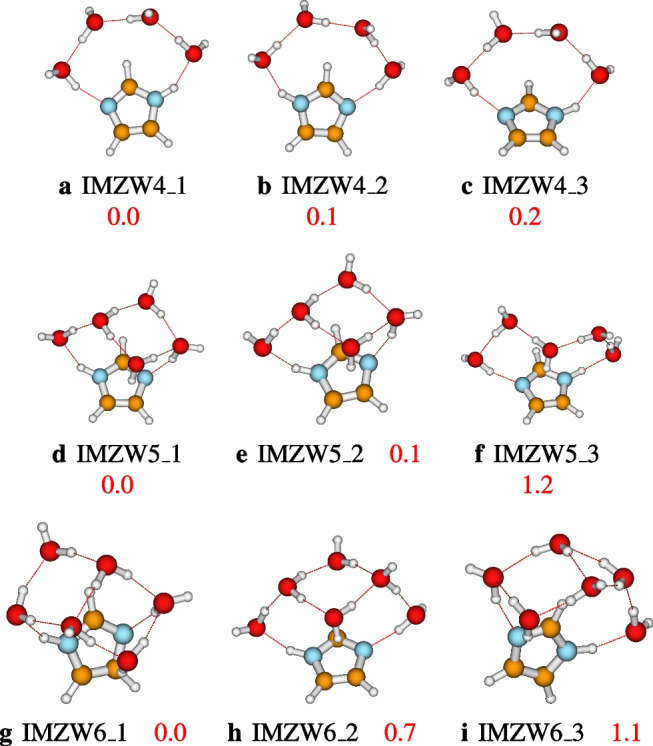
Structures of IMZ(H$_2$O)$_{4-6}$ clusters as optimized at the M06L-D3/def2-TZVPP level of theory. Relative electronic energies are provided in kcal/mol

**Fig. 6:**
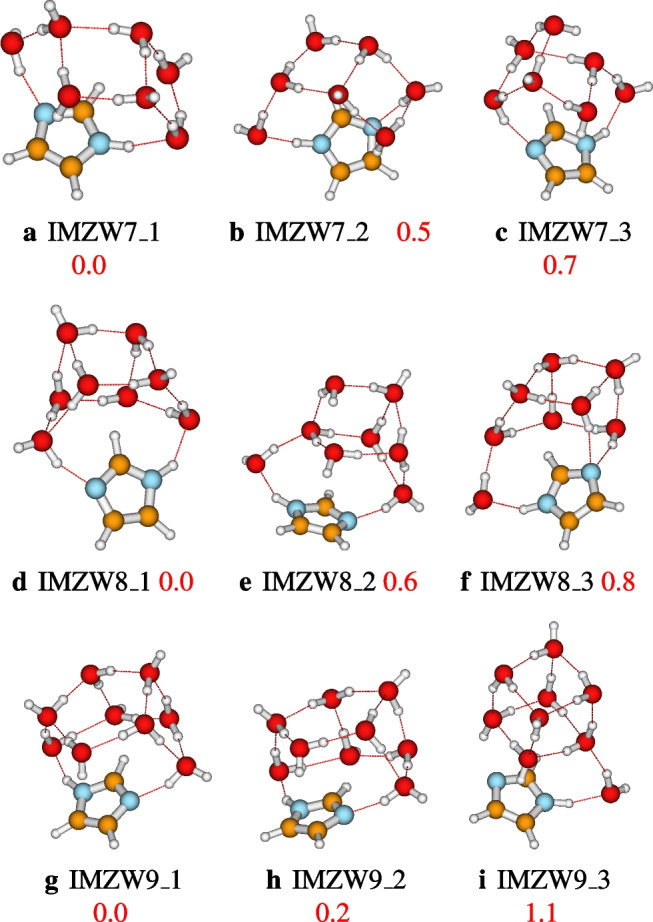
Structures of IMZ(H$_2$O)$_{7-9}$ clusters as optimized at the M06L-D3/def2-TZVPP level of theory. Relative electronic energies are provided in kcal/mol

**Fig. 7:**
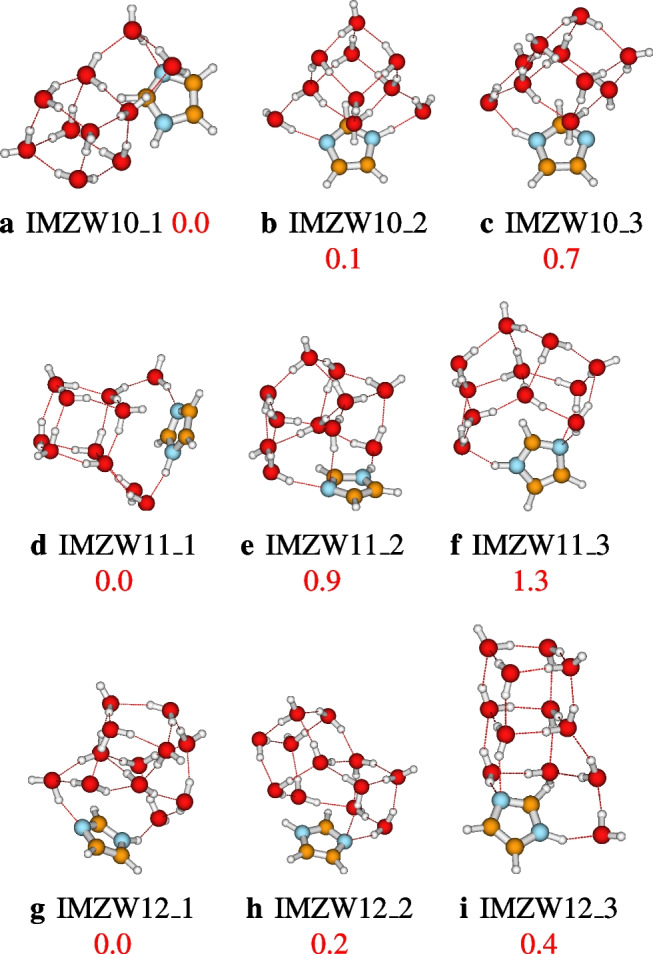
Structures of IMZ(H$_2$O)$_{10-12}$ clusters as optimized at the M06L-D3/def2-TZVPP level of theory. Relative electronic energies are provided in kcal/mol

After identifying the most accurate DFT functional suitable for studying imidazole-water clusters, the structures located using ABCluster are optimized at the M06L-D3/def2-TZVPP level of theory. To avoid a cumbersome presentation of the structures, only the three most stable structures are presented in the main manuscript. These structures are reported in Figs. 4, 5, 6, and 7. For $n=1-12$, the number of structures reported per size is 3, 3, 9, 17, 19, 17, 20, 26, 23, 30, 28, and 30, respectively. A total of 225 structures of IMZ(H$_2$O)$_{1-12}$ have been located at the M06L-D3/def2-TZVPP level of theory.

Three structures of imidazole-water monomer are located, all within 0.9 kcal/mol. In the most stable structure (**IMZW1_1**), imidazole acts as a hydrogen bond acceptor through N3, while the water molecule acts as a hydrogen bond donor. **IMZW1_2** has a similar hydrogen bond network. The difference between **IMZW1_1** and **IMZW1_2** is the orientation of the hydrogen bond in the imidazole plane (see Fig. 4). In the third structure, **IMZW1_3**, lying 0.9 kcal/mol above the most stable, imidazole acts as a hydrogen bond donor through N1. It has been found that the hydrogen bondings in all three structures are in the imidazole plane. It is important to recall that a previous investigation at the MP2/6-31 G(d) level of theory has reported two possible structures: **IMZW1_1** and **IMZW1_3** [36]. Recently, Melli, Barone, and Puzzarini [35] have used a semi-experimental approach to confirm the existence of two isomers of the imidazole-water monomer. Experimental observations indicate that the imidazole N3 atom establishes two out-of-plane hydrogen bonds with two water molecules, while the imidazole N1 atom establishes one in-plane hydrogen bonding with one water molecule [2, 10]. This observation can not be confirmed with one water molecule and will be further discussed later in this work.

The exploration of the PES of the imidazole-water dimer allowed us to locate three stable configurations (see Fig. 4). The most stable structure, **IMZW2_1**, is obtained by attaching one water molecule to the most stable monomer, **IMZW1_1**, as a hydrogen bond donor to the first water molecule. In addition to the hydrogen bonding established with the first water molecule, the second water molecule also establishes a CH$\cdots$O hydrogen bond with CH of imidazole. It can be seen that **IMZW2_1** has three hydrogen bondings while the other two isomers have only two hydrogen bondings. The smaller number of hydrogen bondings explains the lower stability of **IMZW2_2** and **IMZW2_3**. All three isomers can be built from monomers by adding one water molecule.

For IMZ(H$_2$O)$_{3}$, nine structures are located on its PES at the M06L-D3/def2-TZVPP level of theory. The first three most stable structures (reported in Fig. 4) have the same hydrogen bond network; the difference lies in the orientation of the free OH groups in water molecules. N1 acts as a proton donor in these structures, while N3 acts as a proton acceptor. The donating water molecule is out of the imidazole plane as predicted experimentally [2, 10]. However, N1H$\cdots$O hydrogen bonding is not collinear as observed experimentally. This is probably due to the small number of explicit water molecules, which is not yet enough to mimic bulk conditions. Besides, the isomer **IMZW3_4** lies 0.9 kcal/mol above the most stable. This isomer has been found to be the most stable by Kerkeni et al. [23]. The difference in the most stable structure could be due to the lack of thorough exploration by Kerkeni et al. [23] or the lack of dispersion corrections in B3LYP used by the authors.

Incremental water addition leads to an “umbrella” configuration of the most stable structures of imidazole-water clusters. This behavior has also been remarked on by Kerkeni et al. [23]. However, apart from $n=4$, the most stable structures located by Kerkeni et al. [23] are not found to be the most stable in this work. As mentioned above, this difference could be due to the authors’ absence of thorough exploration of the PESs and/or the lack of dispersion corrections in the B3LYP functional used. Besides Kerkeni et al. [23], Jagoda-Cwiklik and coworkers have investigated the micro-hydration of imidazole [19]. Jagoda-Cwiklik and coworkers calculated the ionization potential of the microhydrated imidazole up to $n=5$ explicit water molecules [19] at the MP2/aug-cc-pVDZ level of theory. For $n=1-4$, the most stable structures of IMZ(H$_2$O)$_{n}$ located in this work are in agreement with the report of Jagoda-Cwiklik and coworkers [19]. However, for IMZ(H$_2$O)$_{5}$, the most stable structure reported by Jagoda-Cwiklik and coworkers [19] is not found to be the most stable in this work (see Fig. 5).

As will be clearly discussed in the next section, that from $n=3$, both N1H and N3 atoms of imidazole are involved in hydrogen bonding. In addition, as found experimentally, apart from hydrogen bondings established by N1H and N3 atoms, weaker non-covalent interactions occur between imidazole and water molecules [2]. Up to $n=12$, all the water molecules are accumulated on one face of the imidazole plane. The fact that the water molecules are concentrated on one face could be because the number of explicit water molecules ($n=12$) is not enough to allow water molecules on both faces. It is important to mention that the behavior of water molecules concentrated on one face of the imidazole plane has also been observed in phenol-water clusters [29, 47]. Additionally, from $n>7$, the hydrogen bond network in imidazole-water clusters exhibits a cubic or cage configuration. The cubic configuration is similar to the hydrogen bond network of neutral water clusters [25, 33, 48, 59].

After fully exploring the structures of IMZ(H$_2$O)$_{1-12}$ clusters, the two hydrogen bonds accepted by the imidazole N3 atom have not been located. However, it can be seen in all the structures from $n=3$ that the imidazole N3 atom establishes one out-of-plane hydrogen bonding. Furthermore, up to $n=12$, all the water molecules are collected on one face of imidazole. Therefore, if many water molecules are added to imidazole, one would expect a symmetric distribution of water molecules on both faces. This symmetric distribution could lead to the N3 accepting two hydrogen bonds, as observed experimentally. Consequently, in order to test this hypothesis, we examine the case of imidazole explicitly solvated by 64 water molecules.

**Fig. 8:**
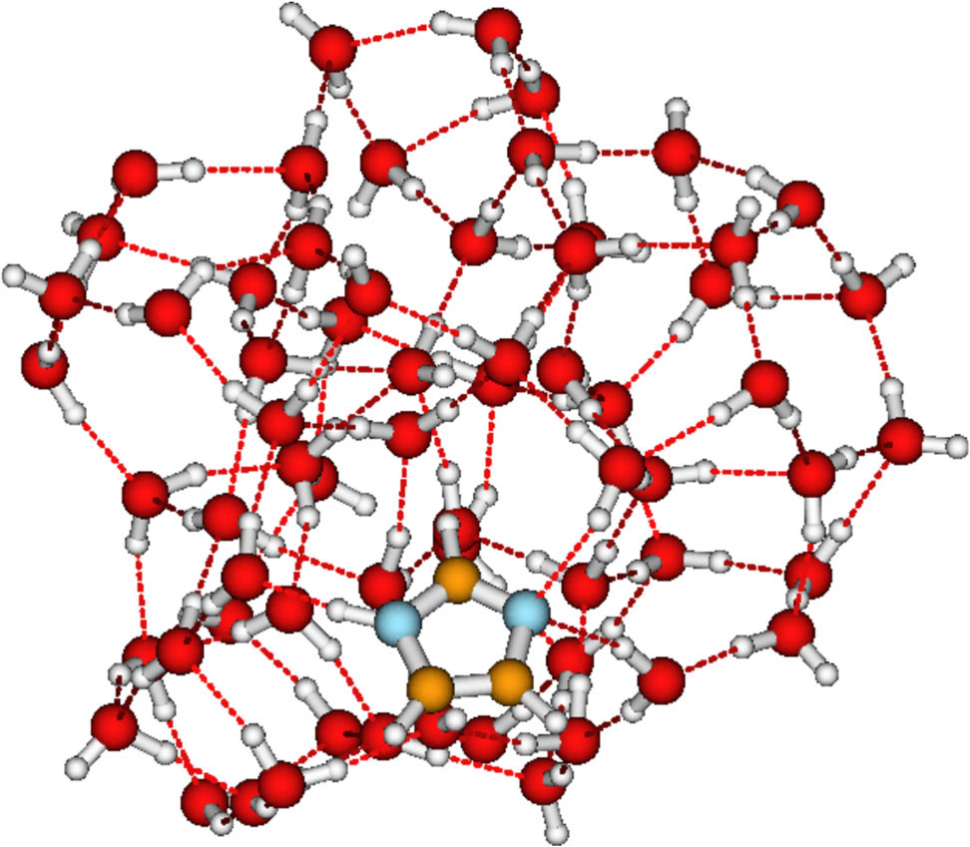
Optimized structure of the imidazole solvated with 64 explicit water molecules at the M06L-D3/def2-TZVPP level of theory

Initial configurations of IMZ(H$_2$O)$_{64}$ are generated with ABCluster as has been done for small-sized clusters. However, in the case of the $64-mer$, only the most stable structure generated by the ABCluster is further optimized at the M06L-D3/def2-TZVPP level of theory. The optimized structure is represented in Fig. 8. With 64 water molecules, the water molecules are now distributed on both faces of the imidazole plane, although a perfectly symmetric distribution is not achieved. A large number of explicit water molecules is necessary to obtain a perfect distribution with imidazole at the center of the solvated system as the optimized lowest energy geometry. However, it can be confirmed now that imidazole establishes two hydrogen bonding (in the accepting position) through the N3 atom and one collinear hydrogen bonding (in the donor position) through the N1H atoms (see Fig. 8). The quantum chemical calculations at the M06L-D3/def2-TZVPP level of theory are in perfect agreement with experimental observations and molecular dynamics simulations [2, 10, 39]. It is important to note that the present investigation provides the first quantum chemical validation of the experimental interpretation of imidazole hydration.

### Non-covalent interactions in IMZ(H$_2$O)$_n$

To determine the nature of the interactions between imidazole and water molecules, we performed an analysis through the quantum theory of atoms in molecules (QTAIM). QTAIM analysis is performed only on the most stable structures, which are considered a good representation of the whole cluster population. QTAIM analysis is performed based on the M06L-D3/def2-TZVPP optimized geometries. In the quantum theory of atoms in molecules, the topology of the electron density of the system is explored to determine all the critical points. Critical points are the points where the first derivatives of the electron density ($\rho$) vanish. The nature of the critical points is determined by the signs of the second derivatives of $\rho$ (Laplacian of $\rho$). Thus, there are nuclear critical points, ring critical points, cage critical points, and bond critical points. In this study, we are interested in bond critical points (BCPs). A bond critical point is located between two atoms that are connected by a bond path. When the overall sign of $\Delta \rho$ at a BCP is negative, the corresponding bonding is a covalent bond. Otherwise, the corresponding bonding is a non-covalent bonding [5, 13, 40]. The strength of the bonding is proportional to the value of the electron density at the bond critical point. The higher the electron density, the stronger the bonding.

**Fig. 9:**
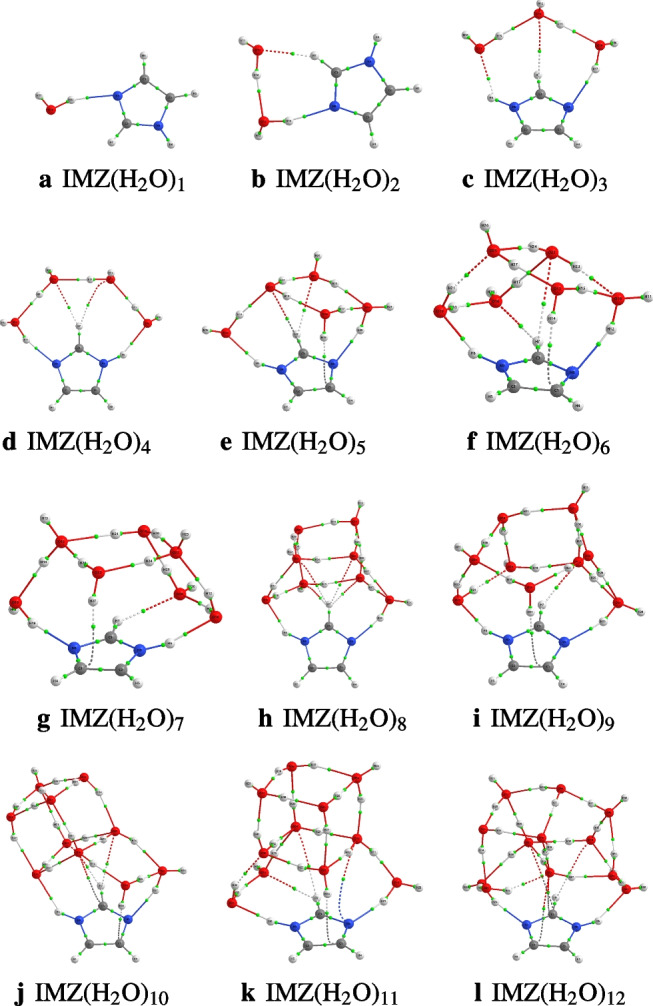
Low-lying energy structures with their bond paths and bond critical points determined from QTAIM analysis. Small green spheres are bond critical points

Bond critical points of the most stable structures, accompanied by the electron density values, the Laplacian of the electron density, the ellipticity, the kinetic energy density, and the distance between the bond path and the geometric bond length, are provided as supplementary material. Visual representations of the bond paths and the bond critical points of the most stable structures are provided in Fig. 9. Dash lines indicate non-covalent bondings, while solid lines represent covalent interactions. After thoroughly exploring the BCPs data, we have been interested in the BCPs where the $\Delta \rho$ is positive (non-covalent interactions). It appears that the non-covalent interactions are dominated by strong OH$\cdots$O hydrogen bondings established between water molecules. Since we are interested in the interaction between imidazole and water molecules, further attention is not accorded to OH$\cdots$O hydrogen bondings. Concerning the interactions between imidazole and water molecules, we have identified four types of non-covalent interactions: two hydrogen bondings involving the two nitrogen atoms (OH$\cdots$N3 and N1H$\cdots$O), one weaker hydrogen bonding CH$\cdots$O, and one OH$\cdots \pi$ bonding interaction. The minimum and maximum values of $\rho$ and $\Delta \rho$ at the bond critical points associated with these bonding interactions are reported in Table 2. Considering the value of the electron density at the corresponding bonding interactions, it is clear that the hydrogen bondings OH$\cdots$N3 and N1H$\cdots$O are the strongest interactions, while the CH$\cdots$O and OH$\cdots \pi$ are weaker interactions. The interactions identified in this work perfectly agree with previous experimental observation and molecular dynamics simulations [2, 10, 39].

**Table 2:** Minimum and maximum values of the electron density $\rho$ and the Laplacian of the electron density $\Delta \rho$ at some selected bond critical points between imidazole and water molecules

	$\rho (ea^{-3})$			$\nabla ^{2}(ea^{-5}) \rho$	
Bondig	Min	Max		Min	Max
OH$\cdots$N3	0.0044	0.0482		0.0161	0.0827
N1H$\cdots$O	0.0188	0.0420		0.0804	0.1072
CH$\cdots$O	0.0027	0.0156		0.0127	0.0591
OH$\cdots \pi$	0.0037	0.0123		0.0138	0.0344

$\rho$ and $\Delta \rho$ are reported in atomic unit

**Fig. 10:**
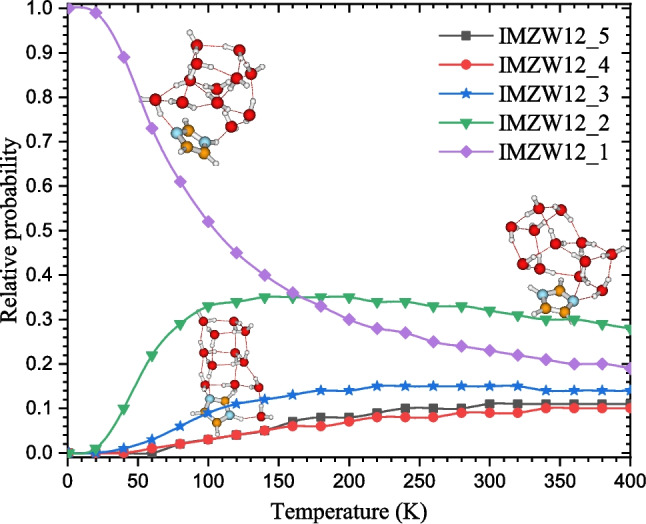
Relative probability calculated using Boltzmann distribution of the structures of IMZ(H$_2$O)$_{12}$ cluster from 0 to 400 K. Structures with a probability lower than 5% are considered to be negligible. The relative population are calculated at the M06L-D3/def2-TZVPP level of theory

### Relative population and clustering incremental energies

It has been shown that the stability of the structures of molecular clusters is temperature-dependent. In other words, the most stable structures at a given temperature differ from those at different temperatures. Temperature-dependence of molecular cluster stability has been assessed in previous works [44, 45, 51, 55]. In this work, we assess the stability of the structures of IMZ(H$_2$O)$_{12}$ for temperatures ranging from 0 to 400 K. The relative probability determined as Boltzmann weights of all the structures is reported in Fig. 10. To avoid cumbersome, only structures with probability above 5% are considered in Fig. 10. Overall, the five most stable structures contribute considerably to the cluster population. IMZW12_1 and IMZW12_2 dominate the population of the IMZ(H$_2$O)$_{12}$ cluster.

**Fig. 11:**
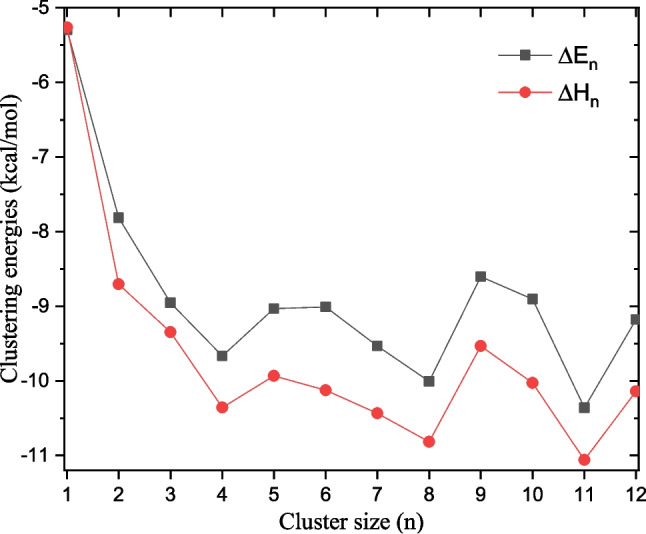
Clustering incremental electronic energy and enthalpy (at room temperature) calculated using Eq. 7

Clustering incremental electronic energies and enthalpy are calculated using the following equation:

7 $$\begin{aligned} \Delta E_n=E_n-E_{n-1}-E(\text {H}_2\text {O}), \end{aligned}$$

where $E_n$ is the energy of IMZ(H$_2$O)$_{n}$ and $E(\text {H}_2\text {O})$ is the energy of the water molecule. The same equation is used to calculate the clustering incremental enthalpy, replacing the electronic energy with the enthalpy. Clustering incremental energy and enthalpy calculated in this work are reported in Fig. 11. It can be seen that the clustering of electronic energy and enthalpy follows the same trend. From $n=1$ to $n=4$, the clustering energy decreases monotonically from ${-5.3}$ kcal/mol to ${-9.7}$kcal/mol. From $n=4$ to $n=12$, the clustering electronic energy curve oscillates around an average. In addition, the clustering enthalpy exhibits the same behavior with lower values due to thermal corrections to enthalpy (see Fig. 11). The oscillations of the clustering energy curve from $n=4$ could indicate that only four water molecules are tightly interacting with imidazole. It could also indicate that the addition of explicit water molecules above $n=4$ has negligible effects on the energetic study of the system. Thus, for the quantum chemistry study of the imidazole solvation, four explicit water molecules could be enough to represent the solvation in a hybrid solvation model.

**Fig. 12:**
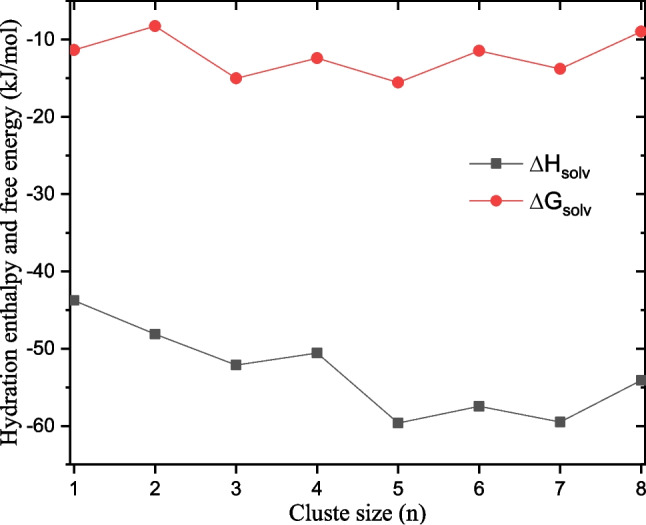
Hydration enthalpy and free energy of imidazole as a function of the number of explicit water molecules

**Fig. 13:**
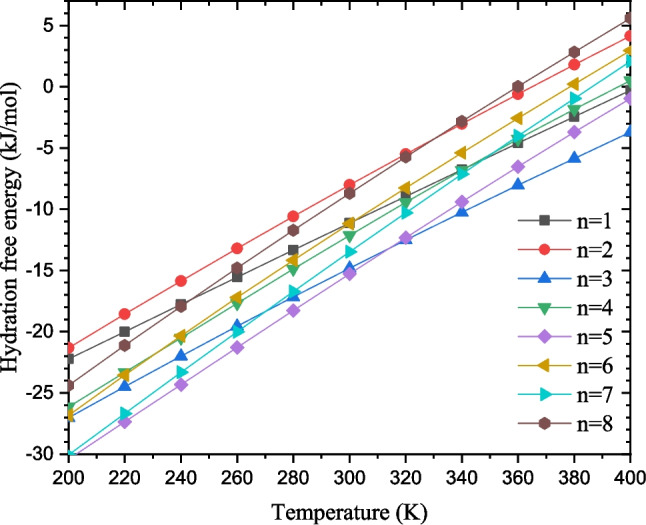
Temperature-dependence of the hydration enthalpy and free energy for cluster sizes $n=1-8$

### Solvation enthalpy and free energy of imidazole in water

Hydration enthalpy and free energy of imidazole are calculated as explained in the methodological section at the M06L-D3/def2-TZVPP level of theory (see the “Hydration enthalpy and free energy” section). To calculate the hydration enthalpy and free energy using Eqs. 2 and 3, the most stable structures of neutral water clusters, (H$_2$O)$_n$, are retrieved from our previous work [28], and re-optimized at the M06L-D3/def2-TZVPP level of theory. It is important to note that the thermodynamic properties are calculated based on rigid-rotor and harmonic approximations. Furthermore, the intermolecular interactions between the molecules are considered negligible. These approximations can affect the accuracy of the calculated thermodynamic properties. Nevertheless, regarding the hydration enthalpy and free energy of imidazole, some error cancellations are expected to occur based on Eqs. 2 and 3. Therefore, these error cancellations considerably reduce the overall error on the hydration enthalpy and free energy of imidazole.

We start by assessing the change of the hydration enthalpy and free energy as a function of the cluster size *n* (see Fig. 12). We have reported the data used to plot Fig. 12 in Table S2 of the supplementary material. Examination of Fig. 12 shows that the hydration enthalpy stabilizes from $n=5$, indicating its convergence. The overall variation of the hydration enthalpy (from $n=1$ to 8) is 15.8 kJ mol$^{-1}$, while from $n=5$ to $n=7$, its variation is within 2.2 kJ mol$^{-1}$. As far as the hydration free energy is concerned, it seems to be unaffected by the change in the number of explicit water molecules. The overall change of the hydration free energy is within 7.3 kJ mol$^{-1}$. Nevertheless, it is almost stable from $n=3$ to $n=7$, with a variation of 4.1 kJ mol$^{-1}$ in the range. The hydration enthalpy and free energy are determined as the average of individual values in the stable ranges. The hydration enthalpy is estimated as the average values from $n=5$ to $n=7$, while the hydration free energy is estimated as the average from $n=3$ to $n=7$. Thus, the hydration enthalpy and free energy of imidazole are estimated to be ${-58.9}$ kJ mol$^{-1}$ and ${-13.6}$ kJ mol$^{-1}$, respectively. It is important to mention that imidazole’s hydration enthalpy and free energy are estimated in this work for the first time.

Temperature-dependence of the hydration enthalpy and free energy is assessed from 200 to 400 K. The hydration enthalpy is almost constant in the range 200 to 400 K. (see data in Table S2 of the supplementary material). The hydration free energy of imidazole increases linearly as a function of temperature from 200 to 400 K (see Fig. 13). The study of the proton’s solvation in ammonia and methanol followed the same trend regarding the hydration enthalpy and free energy [31, 32]. It is important to note that at some temperatures between 360 and 400 K, the hydration free energy of imidazole is positive. This positive value indicates that at high temperatures, the hydration of imidazole is not a spontaneous process and will require some external energy. This behavior of the hydration enthalpy and free energy is also found in the study of the hydration of phenol [29].

## Conclusions

In this work, the interaction of imidazole with water molecules is studied through several aspects. We started by generating initial configurations using classical molecular dynamics. Before performing the optimizations, we carefully selected the most suitable DFT functional:

1. We calculate binding energies of imidazole-water clusters for $n=1-8$ at the DLPNO-CCSD(T1)/CBS level of theory to serve as a benchmark.
2. The binding energies are calculated using twenty candidate DFT functionals.
3. Comparing the DFT binding energies with the benchmark DLPNO-CCSD(T1)/CBS allowed us to select the most suitable function.

The numerical calculations revealed that the smallest mean absolute deviation is found for the M06L-D3 level of theory. Thus, M06L-D3 is used for all the following calculations. DFT benchmarking also revealed that the second most suitable functional is the PW6B95D3 functional.

Structures of IMZ(H$_2$O)$_{n}$, $n=1-12,\;64$, are optimized at the M06L-D3/def2-TZVPP level of theory. The incremental addition of water molecules shows that the water molecules are concentrated on one face of the imidazole plane, which is up to $n=12$. We investigated the IMZ(H$_2$O)$_{64}$ for further insights. It comes out that imidazole establishes two hydrogen bonds (in the accepting position) through the N3 atom and one collinear hydrogen bonding (in the donor position) through the N1H atoms, in agreement with experimental observations. QTAIM analysis shows that, in addition to the strong hydrogen bondings involving the nitrogen atoms, imidazole also establishes weak CH$\cdots$O hydrogen bonding and OH$\cdots \pi$ bonding interactions. The structures optimized in this work are used to calculate imidazole’s hydration enthalpy and free energy for the first time. The hydration enthalpy and free energy are estimated using the cluster continuum solvation model. At room temperature, the hydration enthalpy and free energy of imidazole are estimated to be ${-58.9}$ kJ mol$^{-1}$ and ${-13.6}$ kJ mol$^{-1}$, respectively.

The results of this work can be used for further exploration of imidazole hydration or processes involving imidazole in water.

## Supplementary material

Supplementary material contains the binding energies calculated at the DLPNO-CCSD(T)/CBS and the twenty DFT functionals. It also contains the full list of the structures of imidazole-water clusters from $n=1$ to $n=12$ and their relative electronic energies. Cartesian coordinates of all the structures located in this work are reported in the supporting information.

## Supplementary Information

Below is the link to the electronic supplementary material.

Supplementary file 1 (pdf 11663 KB)

Supplementary file 2 (pdf 480 KB)

## Data Availability

Data is provided within the manuscript or supplementary information files.

## References

[1] Adamo C, Barone V (1999) Toward reliable density functional methods without adjustable parameters: the pbe0 model. J Chem Phys 110:6158–6170

[2] Al-Madhagi LH, Callear SK, Schroeder SL (2020) Hydrophilic and hydrophobic interactions in concentrated aqueous imidazole solutions: a neutron diffraction and total x-ray scattering study. Phys Chem Chem Phys 22:5105–5113 32073011 10.1039/c9cp05993h

[3] Alagona G, Ghio C, Nagy P et al (1990) Comparative study of imidazole hydration: ab initio and electrostatic calculations vs. cambridge structural database analysis. J Comput Chem 9:1038–1046

[4] Austin A, Petersson GA, Frisch MJ et al (2012) A density functional with spherical atom dispersion terms. J Chem Theory Comput 8:4989–5007 26593191 10.1021/ct300778e

[5] Bader RF (1998) A bond path: a universal indicator of bonded interactions. J Phys Chem A 102:7314–7323

[6] Becke AD (1988) Density-functional exchange-energy approximation with correct asymptotic behavior. Phys Rev A 38:3098 10.1103/physreva.38.3098 9900728

[7] Becke AD (1993) Density-functional thermochemistry. III. The role of exact exchange. J Chem Phys 98:5648–5652

[8] Bhattacherjee A, Wategaonkar S (2015) Conformational preferences of monohydrated clusters of imidazole derivatives revisited. Phys Chem Chem Phys 17:20080–20092 26138267 10.1039/c5cp02422f

[9] Chai JD, Head-Gordon M (2008) Long-range corrected hybrid density functionals with damped atom–atom dispersion corrections. Phys Chem Chem Phys 10:6615–6620 18989472 10.1039/b810189b

[10] Duboué-Dijon E, Mason PE, Fischer HE et al (2017) Changes in the hydration structure of imidazole upon protonation: neutron scattering and molecular simulations. J Chem Phys 146

[11] Frisch MJ, Trucks GW, Schlegel HB et al (2016) Gaussian 16 Revision A.03. Gaussian Inc. Wallingford CT

[12] Goerigk L, Hansen A, Bauer C et al (2017) A look at the density functional theory zoo with the advanced gmtkn55 database for general main group thermochemistry, kinetics and noncovalent interactions. Phys Chem Chem Phys 19:32184–32215 29110012 10.1039/c7cp04913g

[13] Grabowski SJ (2011) What is the covalency of hydrogen bonding? Chem Rev 111:2597–2625 21322583 10.1021/cr800346f

[14] Grimme S (2006) Semiempirical gga-type density functional constructed with a long-range dispersion correction. J Comp Chem 27:1787–1799 16955487 10.1002/jcc.20495

[15] Grimme S (2006) Semiempirical gga-type density functional constructed with a long-range dispersion correction. J Comput Chem 27:1787–1799 16955487 10.1002/jcc.20495

[16] Grimme S, Antony J, Ehrlich S et al (2010) A consistent and accurate ab initio parametrization of density functional dispersion correction (dft-d) for the 94 elements h-pu. J Chem Phys 132:154104 20423165 10.1063/1.3382344

[17] Guo Y, Riplinger C, Becker U et al (2018) Communication: an improved linear scaling perturbative triples correction for the domain based local pair-natural orbital based singles and doubles coupled cluster method [dlpno-ccsd (t)]. J Chem Phys 148 10.1063/1.5011798 29306283

[18] Helgaker T, Klopper W, Koch H et al (1997) Basis-set convergence of correlated calculations on water. J Chem Phys 106:9639–9646

[19] Jagoda-Cwiklik B, Slavícek P, Cwiklik L et al (2008) Ionization of imidazole in the gas phase, microhydrated environments, and in aqueous solution. J Phys Chem A 112:3499–3505 18335914 10.1021/jp711476g

[20] Jiang Y, Wang Y, Sanvito S et al (2022) Density functional study on the deprotonation and binding mechanism of imidazole on gold electrodes in an aqueous environment. J Phys Chem C 126:12424–12434

[21] Keith TA (2019) Tk gristmill software. Overland Park KS, USA 11:16. (aim.tkgristmill.com)

[22] Kendall RA, Dunning TH Jr, Harrison RJ (1992) Electron affinities of the first-row atoms revisited. systematic basis sets and wave functions. J Chem Phys 96:6796–6806

[23] Kerkeni B, Bacchus-Montabonel MC (2020) Proton-induced charge transfer on imidazole and 2-aminoimidazole. Role of the substituent and influence of stepwise hydration. J Phys Chem A 124:1003–1010 31935089 10.1021/acs.jpca.9b10602

[24] Lee C, Yang W, Parr RG (1988) Development of the Colle-Salvetti correlation-energy formula into a functional of the electron density. Phys Rev B 37:785 10.1103/physrevb.37.785 9944570

[25] Liu X, Lu WC, Wang C et al (2011) Energetic and fragmentation stability of water clusters (h2o)n, n = 2-30. Chem Phys Lett 508:270–275

[26] Malenov DP, Ninković DB, Zarić SD (2015) Stacking of metal chelates with benzene: can dispersion-corrected DFT be used to calculate organic-inorganic stacking? Chem Phys Chem 16:761–768 25630762 10.1002/cphc.201402589

[27] Malloum A, Conradie J (2021) Accurate binding energies of ammonia clusters and benchmarking of hybrid DFT functionals. Comput Theor Chem 1200

[28] Malloum A, Conradie J (2021) Structures of water clusters in the solvent phase and relative stability compared to gas phase. Polyhedron 193:114856

[29] Malloum A, Conradie J (2023) Microsolvation of phenol in water: structures, hydration free energy and enthalpy. Mol Simul 49:403–414

[30] Malloum A, Conradie J (2024) Assessing computational methods to calculate the binding energies of dimers of five-membered heterocyclic molecules. J Phys Chem A 128:10775–10784 39659037 10.1021/acs.jpca.4c05409

[31] Malloum A, Fifen JJ, Dhaouadi Z et al (2017) Solvation energies of the proton in ammonia explicitly versus temperature. J Chem Phys 146:134308 28390380 10.1063/1.4979568

[32] Malloum A, Fifen JJ, Conradie J (2018) Solvation energies of the proton in methanol revisited and temperature effects. Phys Chem Chem Phys 20:29184–29206 30427006 10.1039/c8cp05823g

[33] Malloum A, Fifen JJ, Dhaouadi Z et al (2019) Structures, relative stabilities and binding energies of neutral water clusters,. New J Chem 43:13020–13037

[34] Mardirossian N, Head-Gordon M (2017) Thirty years of density functional theory in computational chemistry: an overview and extensive assessment of 200 density functionals. Mol Phys 115:2315–2372

[35] Melli A, Barone V, Puzzarini C (2021) Unveiling bifunctional hydrogen bonding with the help of quantum chemistry: the imidazole-water adduct as test case. J Phys Chem A 125:2989–2998 33818109 10.1021/acs.jpca.1c01679 PMC8154618

[36] Nagy PI, Durant G, Smith DA (1993) Theoretical studies on hydration of pyrrole, imidazole, and protonated imidazole in the gas phase and aqueous solution. J Am Chem Soc 115:2912–2922

[37] Neese F (2012) The orca program system. Wiley Interdiscip Rev Comput Mol Sci 2:73–78

[38] Neese F, Valeev EF (2011) Revisiting the atomic natural orbital approach for basis sets: robust systematic basis sets for explicitly correlated and conventional correlated ab initio methods? J Chem Theory Comput 7:33–43 26606216 10.1021/ct100396y

[39] Pagliai M, Funghi G, Vassetti D et al (2019) Imidazole in aqueous solution: hydrogen bond interactions and structural reorganization with concentration. J Phys Chem B 123:4055–4064 31002509 10.1021/acs.jpcb.9b01611

[40] Parthasarathi R, Subramanian V, Sathyamurthy N (2006) Hydrogen bonding without borders: an atoms-in-molecules perspective. J Phys Chem A 110:3349–3351 16526611 10.1021/jp060571z

[41] Perdew JP (1986) Density-functional approximation for the correlation energy of the inhomogeneous electron gas. Phys Rev B 33:8822 10.1103/physrevb.33.8822 9938299

[42] Perdew JP, Wang Y (1992) Accurate and simple analytic representation of the electron-gas correlation energy. Phys Rev B 45:13244 10.1103/physrevb.45.13244 10001404

[43] Perdew JP, Burke K, Ernzerhof M (1996) Generalized gradient approximation made simple. Phys Rev Lett 77:3865 10062328 10.1103/PhysRevLett.77.3865

[44] Pérez C, Muckle MT, Zaleski DP et al (2012) Structures of cage, prism, and book isomers of water hexamer from broadband rotational spectroscopy. Science 336:897–901 10.1126/science.1220574 22605772

[45] Rakshit A, Yamaguchi T, Asada T et al (2017) Understanding the structure and hydrogen bonding network of (h 2 o) 32 and (h 2 o) 33: an improved Monte Carlo temperature basin paving (MCTBP) method and quantum theory of atoms in molecules (QTAIM) analysis. RSC Adv 7:18401–18417

[46] Riplinger C, Pinski P, Becker U et al (2016) Sparse maps-a systematic infrastructure for reduced-scaling electronic structure methods. II. Linear scaling domain based pair natural orbital coupled cluster theory. J Chem Phys 144 10.1063/1.4939030 26772556

[47] Roth W, Schmitt M, Jacoby C et al (1998) Double resonance spectroscopy of phenol: evidence for ice-like structures in aromate–water clusters? Chem Phys 239:1–9

[48] Sahu N, Singh G, Nandi A et al (2016) Toward an accurate and inexpensive estimation of CCSD (T)/CBS binding energies of large water clusters. J Phys Chem A 120:5706–5714 27351269 10.1021/acs.jpca.6b04519

[49] Saitow M, Becker U, Riplinger C et al (2017) A new near-linear scaling, efficient and accurate, open-shell domain-based local pair natural orbital coupled cluster singles and doubles theory. J Chem Phys 146 10.1063/1.4981521 28456208

[50] Sharma B, Rao JS, Sastry GN (2011) Effect of solvation on ion binding to imidazole and methylimidazole. J Phys Chem A 115:1971–1984 21332239 10.1021/jp1120492

[51] Shields RM, Temelso B, Archer KA et al (2010) Accurate predictions of water cluster formation, . J Phys Chem A 114:11725–11737 20882961 10.1021/jp104865w

[52] Stoychev GL, Auer AA, Neese F (2017) Automatic generation of auxiliary basis sets. J Chem Theory Comput 13:554–562 28005364 10.1021/acs.jctc.6b01041

[53] Tao J, Perdew JP, Staroverov VN et al (2003) Climbing the density functional ladder: nonempirical meta–generalized gradient approximation designed for molecules and solids. Phys Rev Lett 91:146401 14611541 10.1103/PhysRevLett.91.146401

[54] Vanommeslaeghe K, Hatcher E, Acharya C et al (2010) CHARMM general force field: a force field for drug-like molecules compatible with the CHARMM all-atom additive biological force fields. J Comput Chem 31:671–690 19575467 10.1002/jcc.21367 PMC2888302

[55] Wang Y, Babin V, Bowman JM et al (2012) The water hexamer: cage, prism, or both. full dimensional quantum simulations say both. J Am Chem Soc 134:11116–11119 22731508 10.1021/ja304528m

[56] Yanai T, Tew DP, Handy NC (2004) A new hybrid exchange-correlation functional using the coulomb-attenuating method (cam-b3lyp). Chem Phys Lett 393:51–57

[57] Yu HS, He X, Li SL et al (2016) Correction: MN15: a Kohn-Sham global-hybrid exchange–correlation density functional with broad accuracy for multi-reference and single-reference systems and noncovalent interactions. Chem Sci 7:6278–6279 30124670 10.1039/c6sc90044e PMC6063197

[58] Yu HS, He X, Li SL et al (2016) MN15: a Kohn-Sham global-hybrid exchange–correlation density functional with broad accuracy for multi-reference and single-reference systems and noncovalent interactions. Chem Sci 7:5032–5051 30155154 10.1039/c6sc00705h PMC6018516

[59] Yuan D, Li Y, Ni Z et al (2017) Benchmark relative energies for large water clusters with the generalized energy-based fragmentation method. J Chem Theory Comput 13:2696–2704 28478670 10.1021/acs.jctc.7b00284

[60] Zhang J, Dolg M (2015) Abcluster: the artificial bee colony algorithm for cluster global optimization. Phys Chem Chem Phys 17:24173–24181 26327507 10.1039/c5cp04060d

[61] Zhang J, Dolg M (2016) Global optimization of clusters of rigid molecules using the artificial bee colony algorithm. Phys Chem Chem Phys 18:3003–3010 26738568 10.1039/c5cp06313b

[62] Zhao Y, Truhlar DG (2005) Design of density functionals that are broadly accurate for thermochemistry, thermochemical kinetics, and nonbonded interactions. J Phys Chem A 109:5656–5667 16833898 10.1021/jp050536c

[63] Zhao Y, Truhlar DG (2008) The m06 suite of density functionals for main group thermochemistry, thermochemical kinetics, noncovalent interactions, excited states, and transition elements: two new functionals and systematic testing of four m06-class functionals and 12 other functionals. Theor Chem Acc 120:215–241

[64] Zhao Y, Schultz NE, Truhlar DG (2005) Exchange-correlation functional with broad accuracy for metallic and nonmetallic compounds, kinetics, and noncovalent interactions. J Chem Phys 123:161103 10.1063/1.2126975 16268672

[65] Zhao Y, Schultz NE, Truhlar DG (2006) Design of density functionals by combining the method of constraint satisfaction with parametrization for thermochemistry, thermochemical kinetics, and noncovalent interactions. J Chem Theory Comput 2:364–382 26626525 10.1021/ct0502763

[66] Zhong S, Barnes EC, Petersson GA (2008) Uniformly convergent n-tuple- augmented polarized (n zap) basis sets for complete basis set extrapolations. i. self-consistent field energies. J Chem Phys 129:184116 19045395 10.1063/1.3009651

