# Supplementary material for: Interactions of imidazole with water molecules
Source: J Mol Model. 2025 Sep 25;31(10):278. doi: 10.1007/s00894-025-06515-4 (PMC12464142; doi:10.1007/s00894-025-06515-4)
Supplement: Supplementary file 1 — (pdf 11663 KB) [file 894_2025_6515_MOESM1_ESM.pdf]

Interactions of Imidazole with Water Molecules

Alhadji Malloum[†,◇,*] and Jeanet Conradie[†]

[†] Department of Chemistry, University of the Free State, PO BOX 339, Bloemfontein 9300, South Africa.

[◇] Department of Physics, Faculty of Science, University of Maroua, PO BOX 46, Maroua, Cameroon.

February 11, 2025

SUPPLEMENTARY MATERIAL:

1 Data description

This supplementary material contains:

- Benchmark binding energies calculated at the DLPNO-CCSD(T1)/CBS level of theory, and binding energies calculated using twenty DFT functionals (see Table 2).
- Structures and relative energies of the imidazole-water clusters, $IMZ(H_2O)_n$, for $n = 1 - 12$ (see Figure 1 to Figure 10).

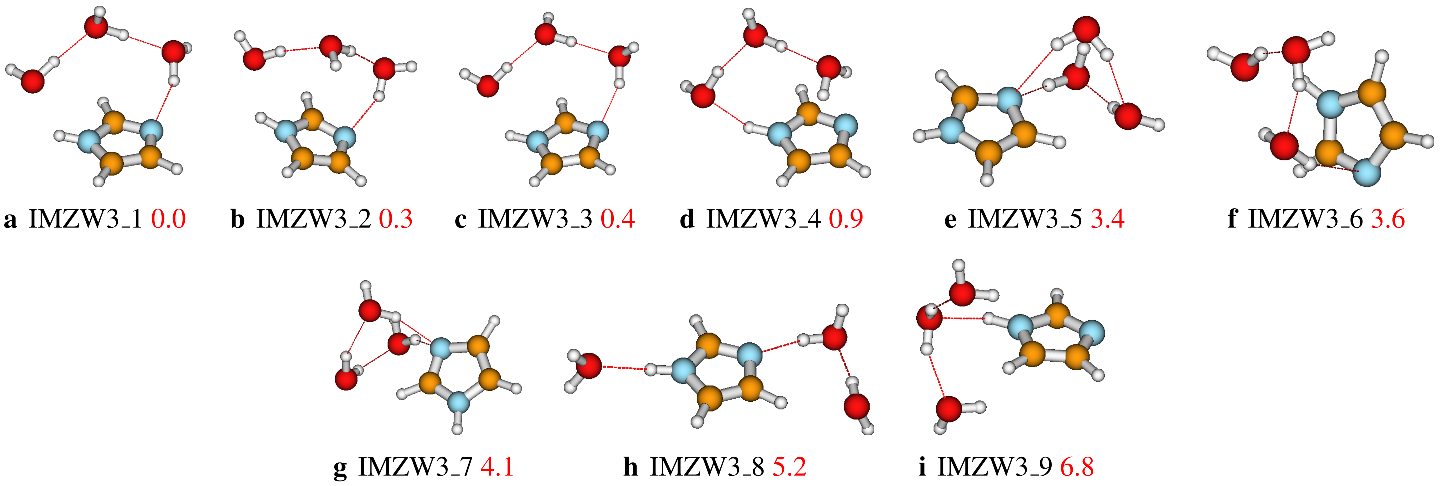

Figure S1 Structures of $IMZ(H_2O)_3$ clusters as optimized at the M06L-D3/def2-TZVPP level of theory. Relative electronic energies are provided in kcal/mol.

* *E-mail: almayega@gmail.com; MalloumA@ufs.ac.za*

Table S1 Binding energies of the hydrated imidazole calculated using twenty DFT functionals associated with the def2TZVPP basis set. CCSD(T)/CBS stands for DLPNO-CCSD(T)/CBS, MAD is mean absolute deviation, MAX is the maximum deviation, and STD is the standard deviation.

n	APFD	B3LYP-D3	B3PW91-D3	B97D3	BLYP-D3	BP86-D3	BPBE-D3	CAM-B3LYP-D3	M05-D3	M06HF-D3	M06-D3	M06L-D3	M052X-D3	M062X-D3	MN15	PBE1PBE-D3	PBE-PBE-D3	PW6B95D3	TPSS-TPSS-D3	ωB97XD	CCSD(T)/CBS
1	-6.9	-6.9	-6.4	-6.1	-6.7	-6.6	-6.2	-7.4	-6.8	-6.4	-6.1	-5.8	-6.6	-6.4	-6.3	-7.0	-7.1	-6.4	-6.7	-6.4	-6.0
2	-18.3	-17.9	-16.1	-1.7	-18.1	-18.5	-17.3	-18.9	-16.3	-18.3	-16.6	-15.6	-17.7	-15.7	-17.4	-18.1	-18.9	-16.2	-16.0	-16.8	-15.1
3	-32.9	-32.6	-30.9	-29.5	-32.7	-33.3	-30.9	-34.9	-31.5	-13.1	-30.0	-28.5	-31.6	-31.8	-30.7	-32.8	-34.2	-29.8	-32.2	-30.5	-28.0
4	-47.2	-46.7	-44.4	-42.7	-46.9	-48.1	-44.5	-49.8	-44.9	-48.0	-42.3	-40.4	-44.3	-44.3	-42.2	-47.3	-49.7	-42.3	-46.8	-43.8	-39.9
5	-60.3	-59.5	-56.8	-54.4	-60.3	-62.2	-57.5	-62.9	-57.2	-59.9	-54.9	-52.2	-56.6	-57.1	-55.2	-59.9	-63.3	-53.6	-59.4	-55.6	-49.8
6	-74.3	-73.4	-69.9	-67.2	-74.5	-76.9	-70.7	-77.7	-70.9	-73.4	-68.2	-64.5	-69.8	-70.1	-67.8	-74.1	-78.5	-65.8	-73.4	-68.5	-60.9
7	-88.0	-86.9	-82.7	-79.8	-87.8	-90.6	-83.3	-92.1	-83.7	-88.0	-79.2	-75.5	-82.1	-81.8	-79.1	-88.1	-93.4	-77.9	-87.4	-80.9	-72.5
8	-101.8	-101.1	-95.9	-92.4	-102.3	-105.5	-96.8	-107.2	-97.4	-102.9	-93.2	-88.5	-95.5	-95.6	-91.9	-102.0	-108.2	-90.0	-101.3	-94.1	-84.1
MAD	9.2	8.6	5.8	5.5	9.1	10.7	6.3	11.8	6.6	10.5	4.3	1.9	6.0	5.8	4.3	9.2	12.1	3.2	8.3	5.0	0.0
MAX	17.7	17.0	11.8	13.4	18.3	21.5	12.7	23.2	13.4	18.8	9.1	4.4	11.5	11.5	7.9	18.0	24.2	5.9	17.2	10.0	0.0
STD	5.7	5.4	4.0	4.0	5.9	7.0	4.3	7.2	4.4	5.9	3.0	1.6	3.6	3.9	2.6	5.7	7.8	2.0	5.9	3.2	0.0

Table S2 Variation of the hydration enthalpy and free energy of imidazole as a function of temperature from 200 to 400 K. The hydration enthalpy and free energy are reported in $kJ\,mol^{-1}$.

	Hydration enthalpy								Hydration free energy							
T(K)	$n=1$	$n=2$	$n=3$	$n=4$	$n=5$	$n=6$	$n=7$	$n=8$	$n=1$	$n=2$	$n=3$	$n=4$	$n=5$	$n=6$	$n=7$	$n=8$
200	-44.4	-49.5	-53.5	-52.6	-60.9	-59.5	-61.5	-56.1	-22.2	-21.3	-27.0	-26.1	-30.4	-26.8	-30.1	-24.4
220	-44.3	-49.3	-53.2	-52.2	-60.7	-59.1	-61.1	-55.7	-20.0	-18.6	-24.5	-23.3	-27.4	-23.5	-26.7	-21.1
240	-44.2	-49.0	-52.9	-51.8	-60.4	-58.7	-60.7	-55.3	-17.8	-15.9	-22.0	-20.5	-24.3	-20.3	-23.3	-17.9
260	-44.1	-48.7	-52.7	-51.4	-60.2	-58.3	-60.3	-54.9	-15.6	-13.2	-19.6	-17.7	-21.3	-17.2	-20.0	-14.8
280	-43.9	-48.4	-52.4	-51.0	-59.9	-57.8	-59.9	-54.5	-13.3	-10.6	-17.2	-14.9	-18.3	-14.2	-16.7	-11.7
298.15	-43.8	-48.1	-52.1	-50.6	-59.6	-57.5	-59.5	-54.1	-11.3	-8.3	-15.0	-12.4	-15.6	-11.4	-13.8	-9.0
300	-43.8	-48.1	-52.1	-50.5	-59.6	-57.4	-59.5	-54.1	-11.1	-8.0	-14.8	-12.1	-15.3	-11.2	-13.5	-8.7
320	-43.6	-47.8	-51.8	-50.1	-59.3	-57.0	-59.1	-53.7	-8.9	-5.5	-12.5	-9.4	-12.3	-8.3	-10.3	-5.7
340	-43.4	-47.4	-51.5	-49.7	-59.0	-56.6	-58.7	-53.2	-6.8	-3.0	-10.3	-6.8	-9.4	-5.4	-7.1	-2.8
360	-43.2	-47.1	-51.2	-49.3	-58.7	-56.1	-58.3	-52.8	-4.6	-0.6	-8.0	-4.3	-6.5	-2.6	-4.0	0.0
380	-43.0	-46.8	-50.9	-48.9	-58.4	-55.7	-57.9	-52.4	-2.4	1.8	-5.8	-1.8	-3.7	0.2	-1.0	2.8
400	-42.8	-46.4	-50.6	-48.4	-58.0	-55.3	-57.5	-52.0	-0.3	4.1	-3.7	0.5	-1.0	3.0	2.1	5.6

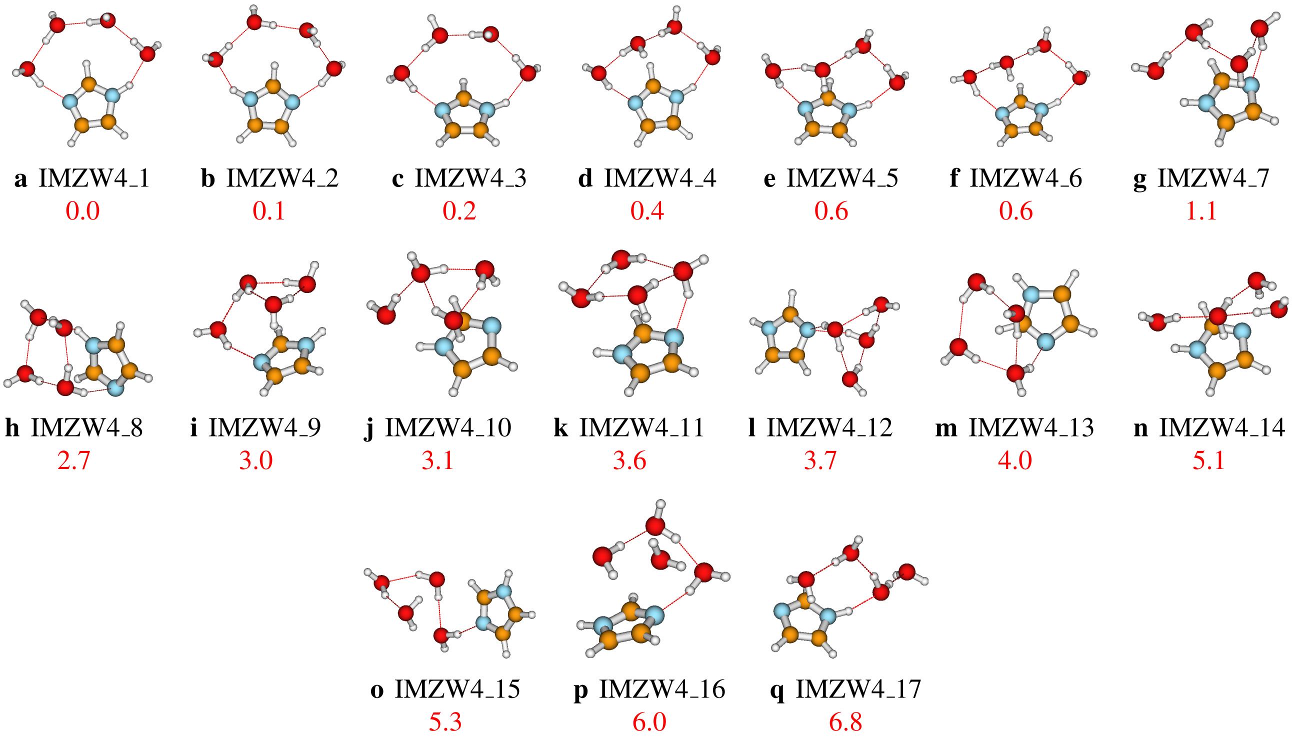

Figure S2 Structures of IMZ(H_2O)$_4$ clusters as optimized at the M06L-D3/def2-TZVPP level of theory. Relative electronic energies are provided in kcal/mol.

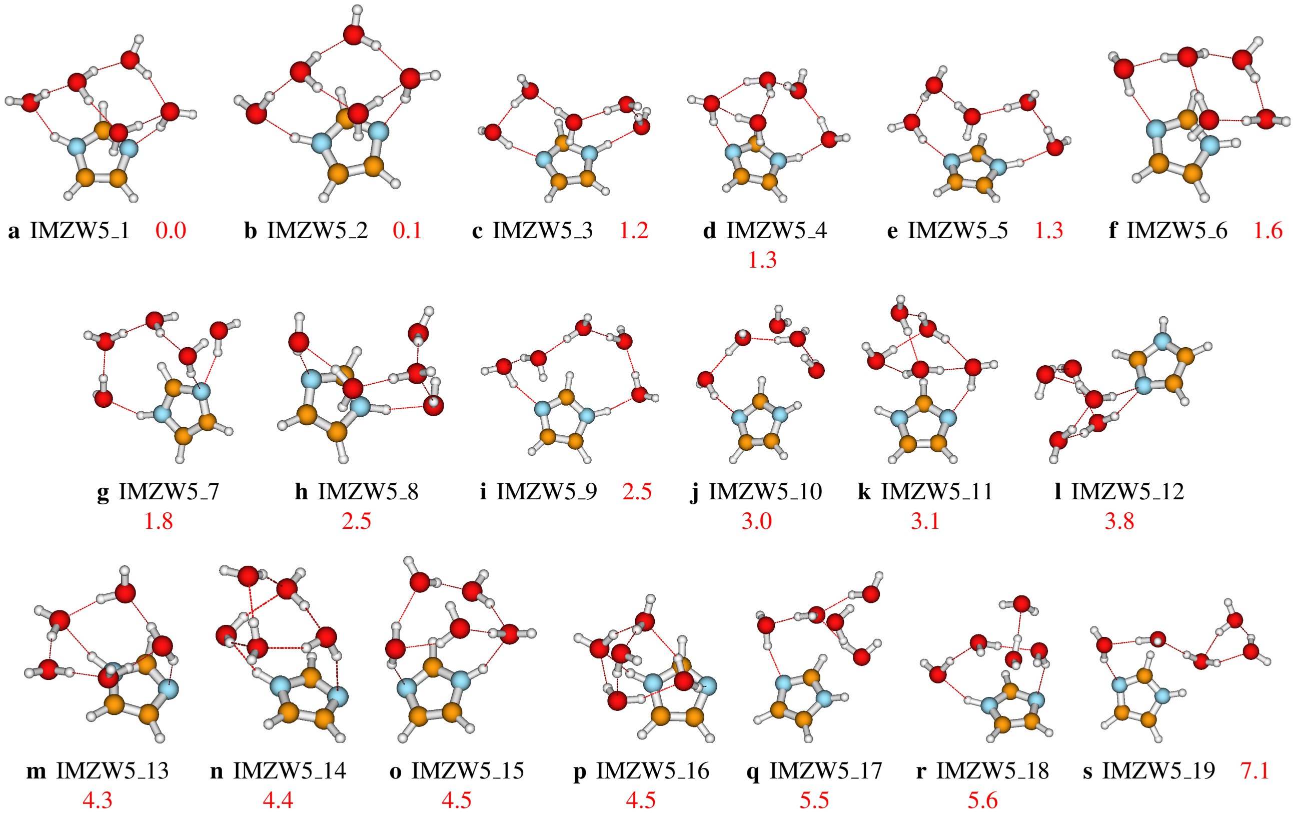

Figure S3 Structures of IMZ(H_2O)$_5$ clusters as optimized at the M06L-D3/def2-TZVPP level of theory. Relative electronic energies are provided in kcal/mol.

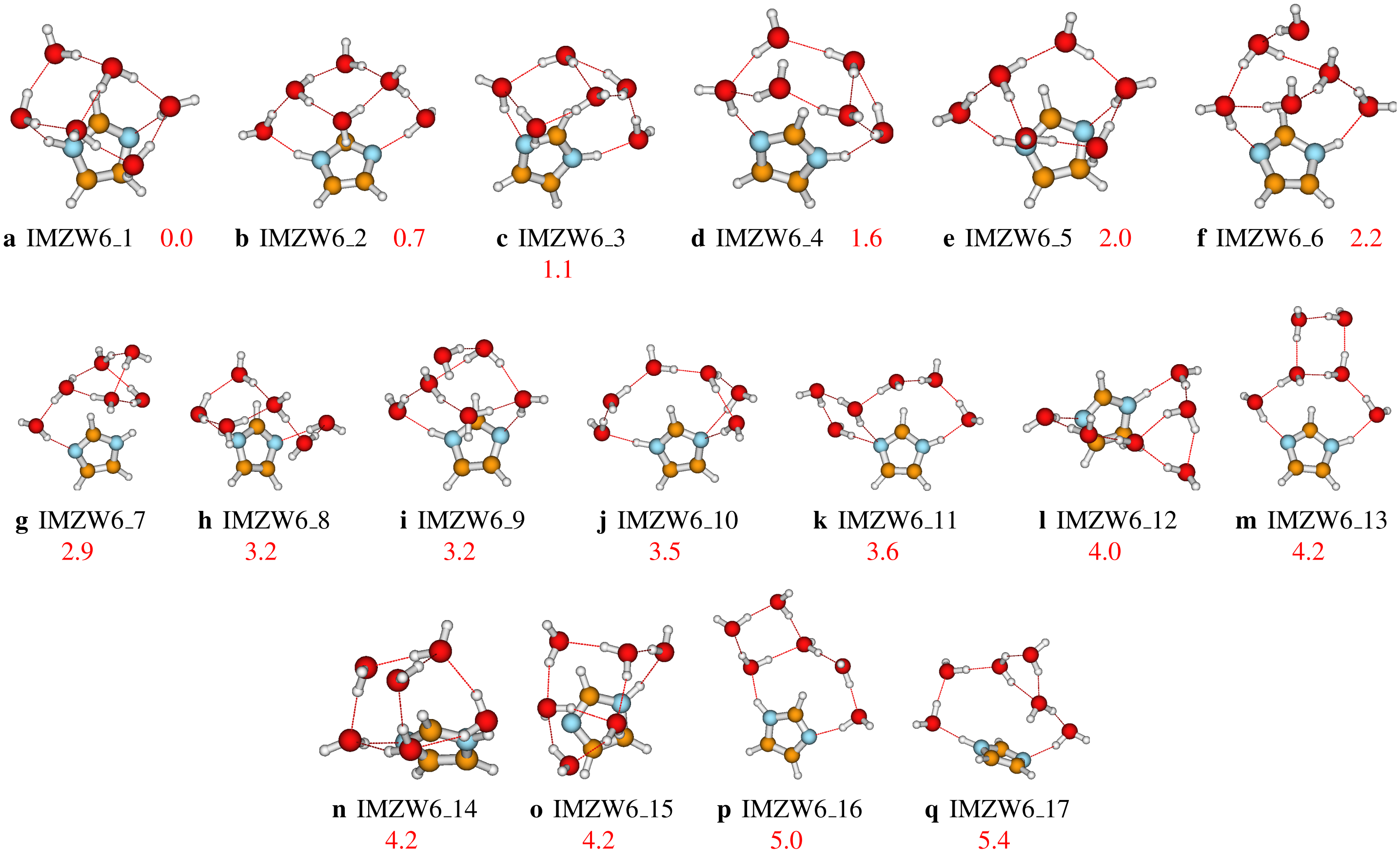

Figure S4 Structures of IMZ(H_2O)$_6$ clusters as optimized at the M06L-D3/def2-TZVPP level of theory. Relative electronic energies are provided in kcal/mol.

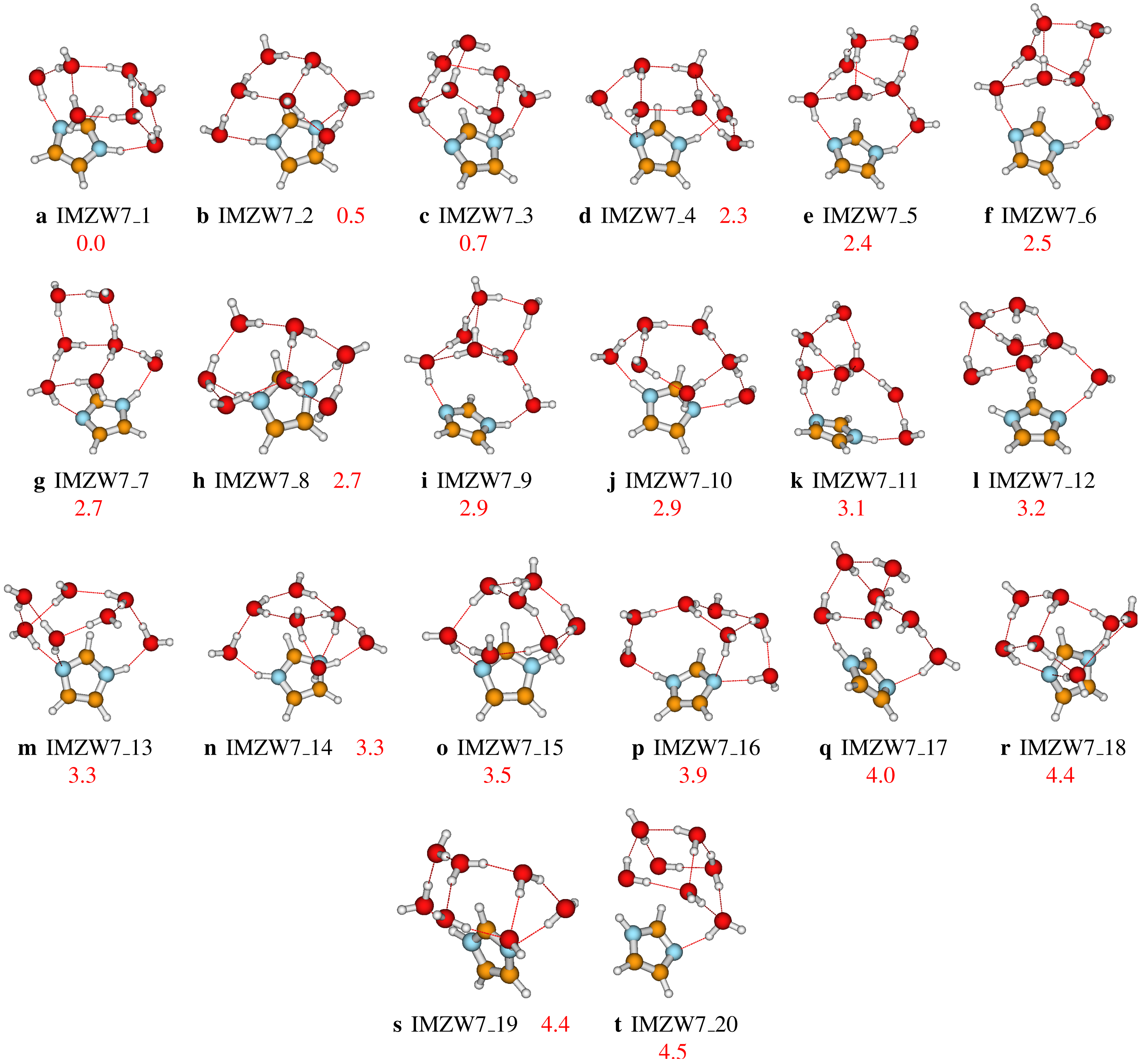

Figure S5 Structures of IMZ$(H_2O)_7$ clusters as optimized at the M06L-D3/def2-TZVPP level of theory. Relative electronic energies are provided in kcal/mol.

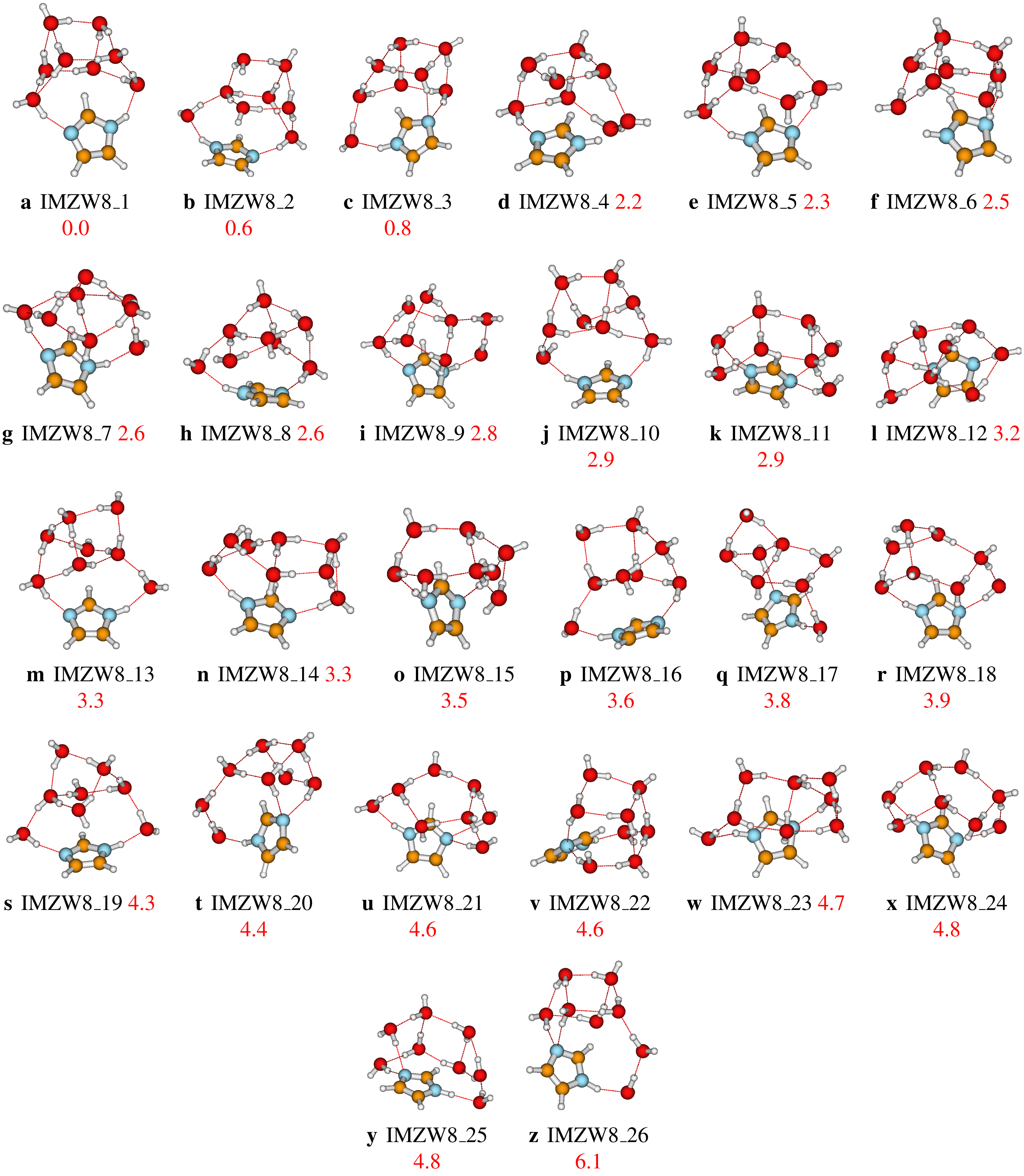

Figure S6 Structures of IMZ(H_2O)$_8$ clusters as optimized at the M06L-D3/def2-TZVPP level of theory. Relative electronic energies are provided in kcal/mol.

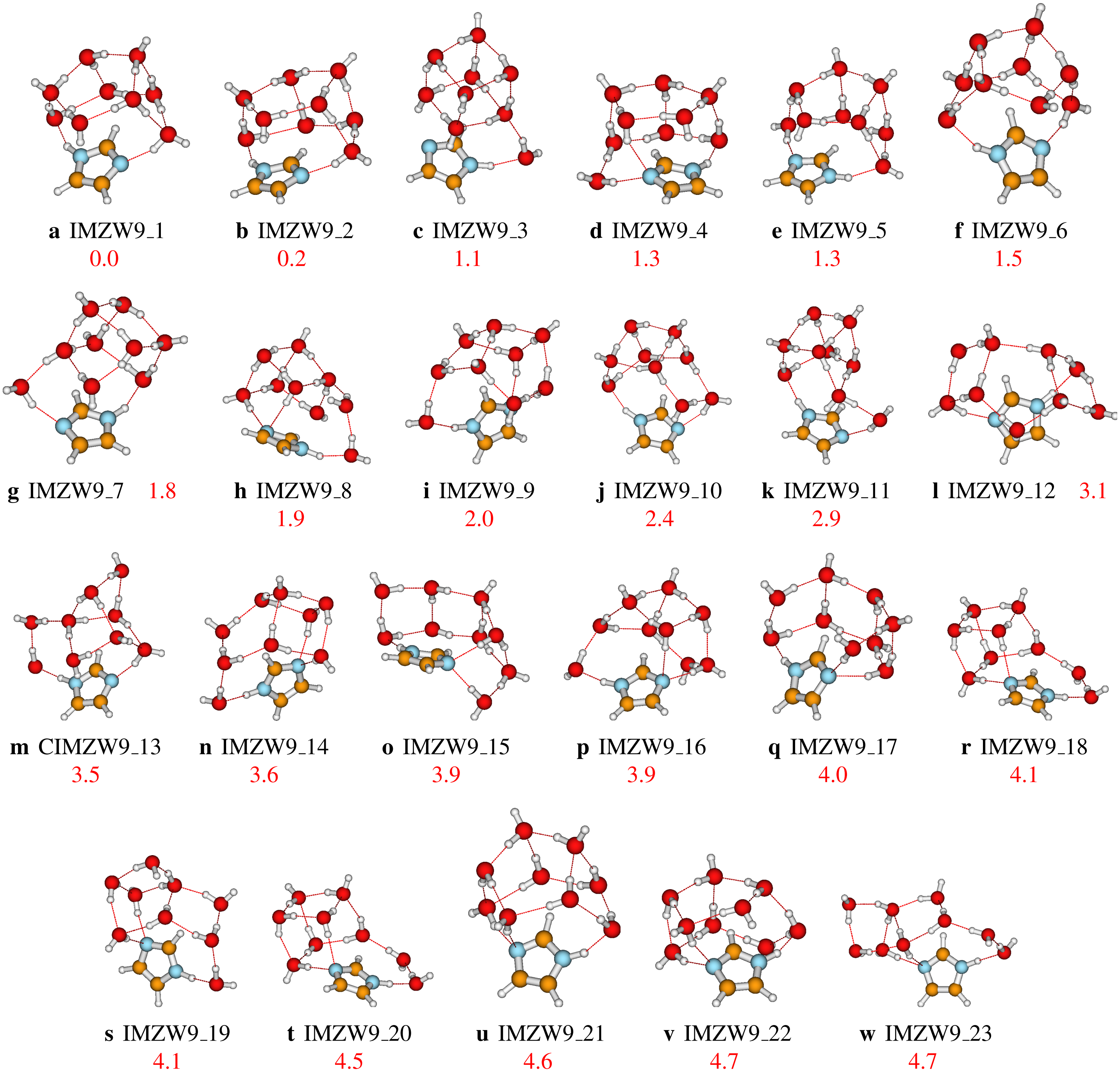

Figure S7 Structures of IMZ(H_2O)$_9$ clusters as optimized at the M06L-D3/def2-TZVPP level of theory. Relative electronic energies are provided in kcal/mol.

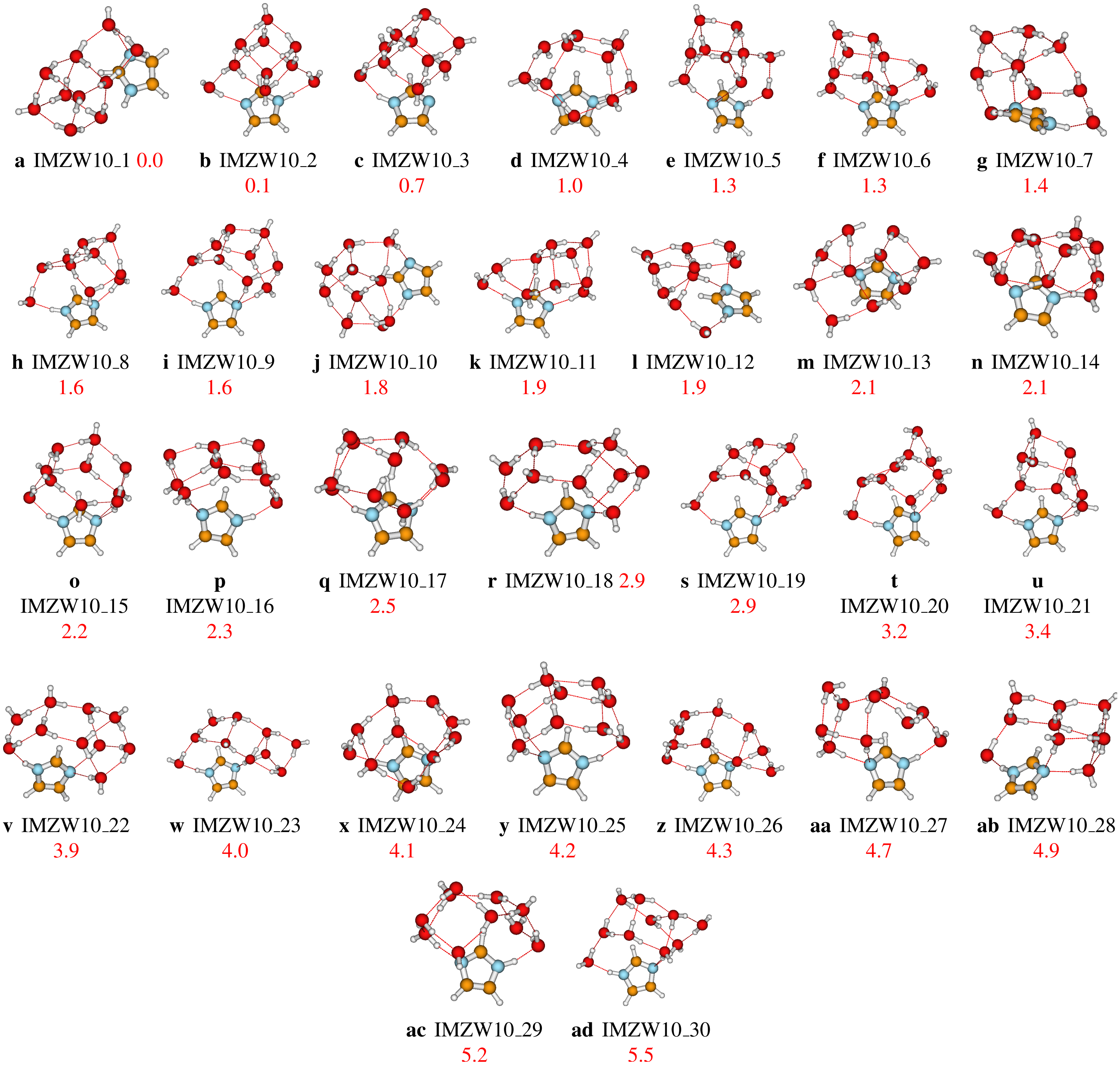

Figure S8 Structures of IMZ(H_2O)$_{10}$ clusters as optimized at the M06L-D3/def2-TZVPP level of theory. Relative electronic energies are provided in kcal/mol.

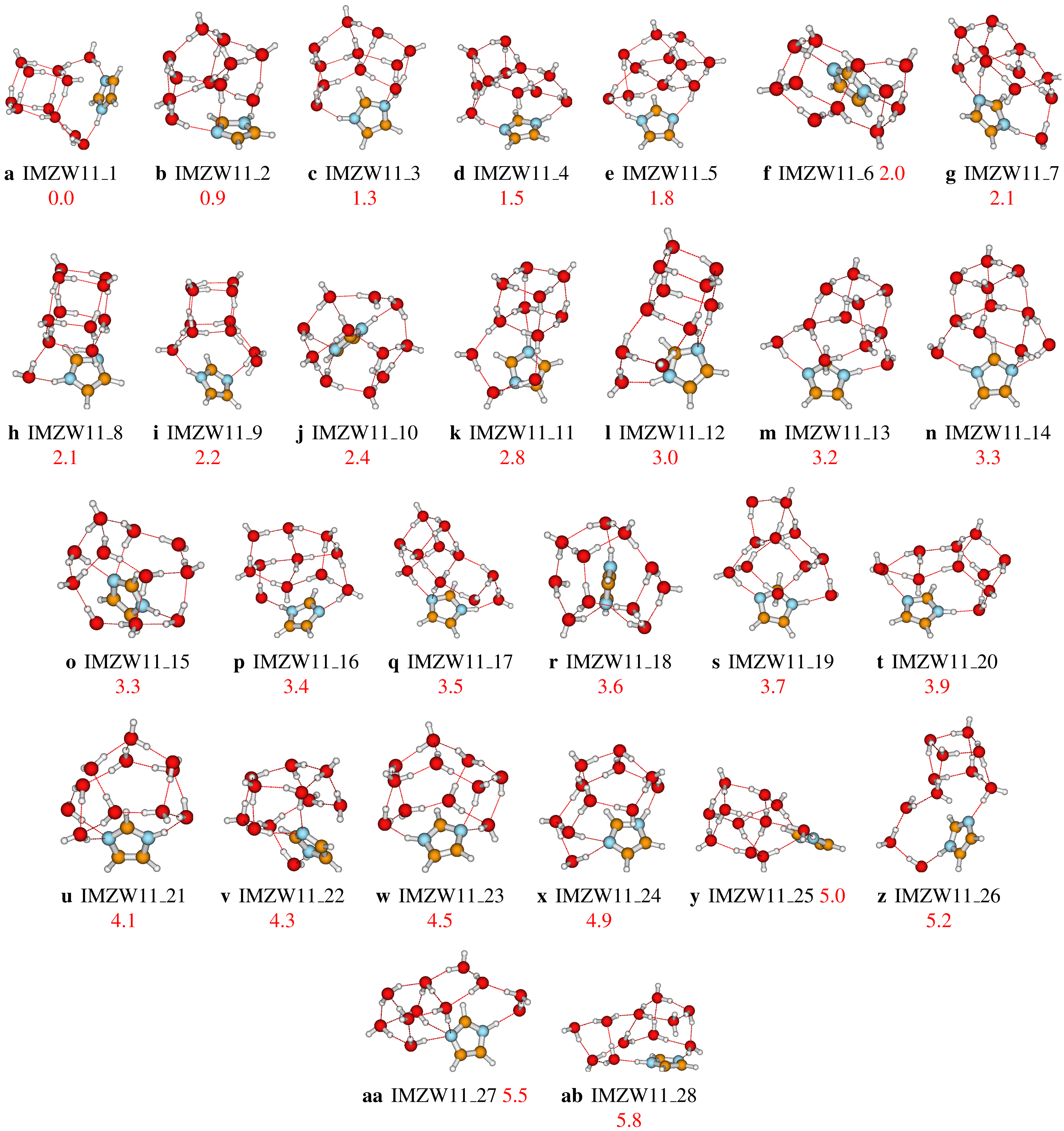

Figure S9 Structures of IMZ(H_2O)$_{11}$ clusters as optimized at the M06L-D3/def2-TZVPP level of theory. Relative electronic energies are provided in kcal/mol.

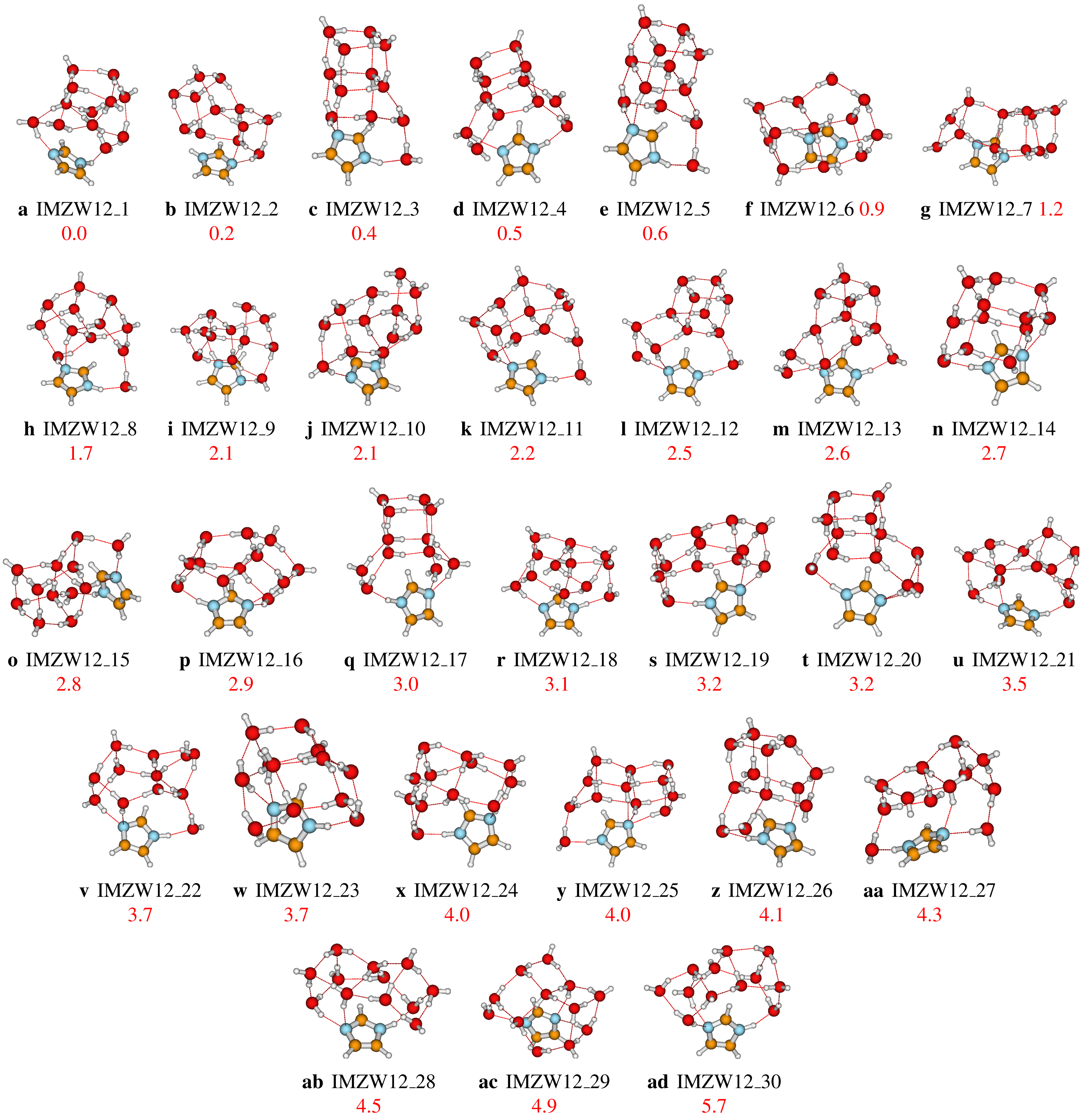

Figure S10 Structures of IMZ$(H_2O)_{12}$ clusters as optimized at the M06L-D3/def2-TZVPP level of theory. Relative electronic energies are provided in kcal/mol.
