# Supplementary material for: Interactions of imidazole with water molecules
Source: J Mol Model. 2025 Sep 25;31(10):278. doi: 10.1007/s00894-025-06515-4 (PMC12464142; doi:10.1007/s00894-025-06515-4)
Supplement: Supplementary file 2 — (pdf 480 KB) [file 894_2025_6515_MOESM2_ESM.pdf]

Cartesian coordinates of : IMZW1_1

Atomic number (AN) and Cartesian coordinates

AN	X	Y	Z
6	0.463656	1.100769	0.017311
6	1.769318	0.712526	-0.011896
6	0.449684	-1.039442	0.004720
1	0.067145	2.101292	0.031461
1	2.691141	1.263644	-0.027430
1	2.539557	-1.266451	-0.039032
1	0.151435	-2.073358	0.004686
7	-0.359231	-0.000212	0.027767
7	1.741420	-0.655963	-0.019428
8	-3.192695	-0.033711	0.083875
1	-2.212295	-0.019083	0.040038
1	-3.466687	0.213756	-0.799908

Cartesian coordinates of : IMZW1_2

Atomic number (AN) and Cartesian coordinates

AN	X	Y	Z
6	0.475523	1.104551	-0.008090
6	1.776995	0.701998	0.013605
6	0.438422	-1.035454	-0.008647
1	0.089867	2.109372	-0.014642
1	2.704829	1.242942	0.028785
1	2.525829	-1.285318	0.025861
1	0.128882	-2.066036	-0.013847
7	-0.359256	0.012616	-0.022117
7	1.734282	-0.666151	0.012906
8	-3.191968	-0.006447	-0.091109
1	-3.472601	-0.000334	0.824623
1	-2.211886	-0.000873	-0.038632

Cartesian coordinates of : IMZW1_3

Atomic number (AN) and Cartesian coordinates

AN	X	Y	Z
6	0.475542	1.104559	-0.008110
6	1.777007	0.701979	0.013631
6	0.438401	-1.035446	-0.008670
1	0.089909	2.109388	-0.014666
1	2.704852	1.242903	0.028833
1	2.525805	-1.285347	0.025900
1	0.128845	-2.066025	-0.013884

7	-0.359256	0.012640	-0.022164
7	1.734268	-0.666168	0.012928
8	-3.191966	-0.006444	-0.091083
1	-3.472578	-0.000364	0.824656
1	-2.211882	-0.000858	-0.038625

Cartesian coordinates of : IMZW1_4

Atomic number (AN) and Cartesian coordinates

AN	X	Y	Z
6	1.859761	-0.631945	-0.000207
6	0.579545	-1.103473	0.006212
6	0.592772	1.081507	-0.001588
1	2.773931	-1.200619	-0.001719
1	0.173148	-2.098473	0.011044
1	-1.241616	0.017460	0.007137
1	0.210775	2.087928	-0.003887
7	1.867084	0.742585	-0.005048
7	-0.220732	0.005745	0.005393
8	-3.103920	0.089645	-0.017109
1	-3.419153	-0.313366	0.794586
1	-3.382656	-0.524939	-0.699206

Cartesian coordinates of : IMZW2_ 1

Atomic number (AN) and Cartesian coordinates

AN	X	Y	Z
6	-0.781092	-0.175830	1.183562
6	-1.730218	0.573340	0.553128
6	-0.832815	-0.859903	-0.842537
1	-0.467994	-0.122436	2.212061
1	-2.379773	1.358930	0.892149
1	-2.341988	0.459915	-1.477471
1	-0.642848	-1.379313	-1.765926
7	-0.226075	-1.079253	0.306752
7	-1.752047	0.119633	-0.738187
8	1.468514	1.836363	-0.310432
1	0.663614	1.708963	0.199907
1	1.917805	0.977511	-0.233899
8	2.587434	-0.787805	-0.073623
1	1.674720	-1.068991	0.130291
1	3.040486	-0.831334	0.770466

Cartesian coordinates of : IMZW2_ 2

Atomic number (AN) and Cartesian coordinates

AN	X	Y	Z
6	1.629071	0.987697	-0.476592
6	2.531647	-0.016616	-0.294915
6	0.572819	-0.597064	0.502605
1	1.785608	1.948111	-0.936163
1	3.571820	-0.112285	-0.545321
1	2.212243	-1.909316	0.612660
1	-0.181557	-1.199546	0.978306
7	0.403869	0.619362	0.025194
7	1.840822	-1.018836	0.331231
8	-2.867612	-1.268120	-0.493464
1	-3.820634	-1.350073	-0.546446
1	-2.727434	-0.347693	-0.205734
8	-2.283079	1.377999	0.284595
1	-2.432713	1.427350	1.230198
1	-1.315867	1.216639	0.201883

Cartesian coordinates of : IMZW2_ 3

Atomic number (AN) and Cartesian coordinates

AN	X	Y	Z
6	-1.314739	0.816040	-0.859412
6	-2.135007	-0.234088	-0.574143
6	-0.622732	0.004223	0.995336
1	-1.347006	1.471670	-1.712325
1	-2.978596	-0.657672	-1.086702
1	-2.061929	-1.524131	1.111458
1	-0.069587	-0.192870	1.897731
7	-0.371228	0.965203	0.129326
7	-1.678649	-0.739784	0.613411
8	2.450963	1.093384	0.273299
1	2.846838	1.540997	-0.476365
1	1.488975	1.235106	0.160353
8	2.035993	-1.635747	-0.429630
1	2.753380	-1.916327	-0.999377
1	2.256282	-0.712850	-0.213968

Cartesian coordinates of : IMZW2_ 4

Atomic number (AN) and Cartesian coordinates

AN	X	Y	Z
6	0.734381	1.383538	0.000041
6	-0.628821	1.413650	-0.011852
6	0.082952	-0.653137	0.026129
1	1.420039	2.213470	-0.007126
1	-1.333916	2.224376	-0.030272
1	-1.992931	-0.234886	0.002173

1	0.050047	-1.729016	0.042628
7	1.177560	0.083202	0.023972
7	-1.029028	0.105507	0.005239
8	-3.717584	-0.902575	-0.006817
1	-4.135054	-0.565089	-0.802106
1	-4.191545	-0.464066	0.702954
8	3.839610	-0.870764	0.081829
1	2.916473	-0.538034	0.034649
1	4.119880	-0.905319	-0.833383

Cartesian coordinates of : IMZW2_ 5

Atomic number (AN) and Cartesian coordinates

AN	X	Y	Z
6	-2.522115	-0.048014	-0.331830
6	-1.562679	-1.012919	-0.435500
6	-0.764556	0.760638	0.561422
1	-3.542614	-0.091190	-0.672146
1	-1.571201	-2.002129	-0.855596
1	0.475868	-0.916917	0.228673
1	-0.054862	1.397994	1.061139
7	-2.019352	1.066835	0.295584
7	-0.443928	-0.481281	0.143809
8	2.804787	1.196174	-0.553397
1	1.922052	1.571118	-0.507468
1	3.289826	1.673756	0.122932
8	2.289425	-1.373019	0.426651
1	2.502050	-2.002945	-0.264042
1	2.564246	-0.512038	0.060173

Cartesian coordinates of : IMZW2_ 6

Atomic number (AN) and Cartesian coordinates

AN	X	Y	Z
6	2.521901	0.043178	0.277175
6	1.581132	1.023270	0.406438
6	0.713063	-0.759067	-0.513850
1	3.558038	0.079142	0.567414
1	1.622287	2.020503	0.805088
1	-0.488190	0.941528	-0.157712
1	-0.027488	-1.396717	-0.965985
7	1.975228	-1.076997	-0.302198
7	0.428987	0.495375	-0.107196
8	-2.298829	1.439878	-0.211079
1	-2.530038	1.564623	-1.133186
1	-2.565363	0.521616	-0.018991
8	-2.776621	-1.232002	0.486177
1	-2.770776	-1.741234	-0.327412

1	-1.920935	-1.425399	0.877169

Cartesian coordinates of : IMZW2_ 7

Atomic number (AN) and Cartesian coordinates

AN	X	Y	Z
6	-2.594917	0.051826	-0.290497
6	-1.624669	1.008203	-0.366508
6	-0.812405	-0.845741	0.457047
1	-3.629068	0.134694	-0.578304
1	-1.635441	2.026664	-0.709665
1	0.434843	0.846225	0.203457
1	-0.091345	-1.530382	0.869619
7	-2.083133	-1.113903	0.227602
7	-0.489243	0.418756	0.116830
8	2.953804	-1.158431	-0.345520
1	3.100430	-1.188568	-1.293324
1	3.816537	-1.356055	0.025035
8	2.202284	1.415679	0.405944
1	2.560704	0.562821	0.095917
1	2.393210	2.026925	-0.307409

Cartesian coordinates of : IMZW2_ 8

Atomic number (AN) and Cartesian coordinates

AN	X	Y	Z
6	1.485839	0.912664	0.652891
6	0.267411	0.514596	1.125935
6	0.197806	0.789723	-1.040193
1	2.387803	1.087996	1.214055
1	-0.086566	0.286609	2.114952
1	-1.530971	0.168242	0.019892
1	-0.209014	0.813416	-2.036705
7	1.438886	1.088191	-0.709783
7	-0.547592	0.445680	0.030142
8	-3.315870	-0.327295	-0.076189
1	-3.346262	-1.276450	0.060776
1	-3.757868	0.028950	0.697275
8	1.548264	-2.362405	-0.080996
1	1.198371	-2.077519	-0.927713
1	1.539971	-1.552649	0.440627

Cartesian coordinates of : IMZW2_ 9

Atomic number (AN) and Cartesian coordinates

AN X Y Z

6	-1.854799	0.711529	0.007237
6	-1.663459	-0.212941	-0.976327
6	-0.568836	-0.777663	0.837454
1	-2.445162	1.610530	-0.035653
1	-2.022243	-0.286751	-1.986315
1	-0.492836	-1.982383	-0.887356
1	0.061144	-1.357041	1.489787
7	-1.169849	0.353828	1.146919
7	-0.842389	-1.159773	-0.427291
8	1.418274	1.727143	-0.312947
1	1.894992	0.885863	-0.208545
1	0.581836	1.569596	0.134165
8	2.851288	-0.706103	-0.010873
1	3.361920	-0.782529	-0.819673
1	3.512087	-0.509537	0.656566

Cartesian coordinates of : IMZW2_10

Atomic number (AN) and Cartesian coordinates

AN	X	Y	Z
6	1.483250	0.930982	0.634035
6	0.278592	0.520324	1.130918
6	0.168038	0.778676	-1.035550
1	2.392316	1.122148	1.178353
1	-0.055434	0.294937	2.127428
1	-1.534375	0.144021	0.060231
1	-0.255902	0.789844	-2.025135
7	1.410498	1.096165	-0.728802
7	-0.554135	0.432477	0.049852
8	-3.312241	-0.381199	0.128015
1	-3.360069	-1.233011	-0.311053
1	-3.798396	0.203745	-0.456895
8	1.591046	-2.344350	-0.090199
1	1.574610	-1.536892	0.434713
1	1.232982	-2.060784	-0.933941

Cartesian coordinates of : IMZW2_11

Atomic number (AN) and Cartesian coordinates

AN	X	Y	Z
6	1.268521	1.184913	-0.519126
6	0.037068	0.869308	-1.013444
6	0.156394	0.372975	1.111142
1	2.104084	1.614528	-1.044818
1	-0.396631	0.963441	-1.992329
1	-1.626073	0.010461	0.023675
1	-0.152508	0.015148	2.078389

```
  7      1.343429      0.873638      0.819395
  7     -0.663518      0.353631      0.042264
  8     -3.358180     -0.641750      0.054451
  1     -3.941628      0.073468     -0.207458
  1     -3.441876     -1.276641     -0.660008
  8      2.118972     -2.046158     -0.170105
  1      2.017133     -1.202756      0.284731
  1      1.819881     -1.848435     -1.059998
 ---------------------------------------------------

Cartesian coordinates of :  IMZW2_12

 ---------------------------------------------------
 Atomic number (AN) and Cartesian coordinates
  AN          X             Y             Z
 ---------------------------------------------------
  6     -1.159039      1.328682     -0.414898
  6      0.074328      1.029522     -0.914080
  6     -0.154953      0.204237      1.096039
  1     -1.950591      1.882521     -0.889998
  1      0.551642      1.255567     -1.850154
  1      1.650711     -0.073386      0.021558
  1      0.095684     -0.322347      2.001018
  7     -1.303067      0.810785      0.852133
  7      0.704746      0.311282      0.064697
  8     -2.306667     -1.868716     -0.463490
  1     -2.524676     -2.526297      0.199187
  1     -2.125268     -1.076700      0.055155
  8      3.352634     -0.807215     -0.035320
  1      3.625333     -0.787075     -0.955097
  1      3.935645     -0.173947      0.388622
 ---------------------------------------------------

Cartesian coordinates of :  IMZW2_13

 ---------------------------------------------------
 Atomic number (AN) and Cartesian coordinates
  AN          X             Y             Z
 ---------------------------------------------------
  6     -2.049943     -0.088519     -0.587243
  6     -0.863445     -0.537356     -1.090699
  6     -0.764952     -0.383115      1.087011
  1     -2.944276      0.166381     -1.129763
  1     -0.529196     -0.736428     -2.092775
  1      0.934900     -0.982785     -0.023731
  1     -0.350372     -0.432069      2.079495
  7     -1.987516      0.006179      0.782766
  7     -0.054608     -0.732969     -0.004720
  8      2.798360     -1.172306     -0.091015
  1      3.079717     -1.374524      0.803870
  1      3.068213     -0.258966     -0.211836
  8      1.135637      2.367989      0.029407
  1      0.328688      2.026594     -0.361665
  1      1.305254      3.167812     -0.471467
```

Cartesian coordinates of : IMZW3_ 1

Atomic number (AN) and Cartesian coordinates

AN	X	Y	Z
6	0.790105	-0.330071	1.073144
6	1.918380	0.433471	1.071500
6	1.676567	-0.576241	-0.861634
1	0.075572	-0.464860	1.867207
1	2.369707	1.065044	1.813855
1	3.314784	0.692220	-0.507325
1	1.888690	-0.874831	-1.873538
7	0.645557	-0.965962	-0.137746
7	2.470858	0.263376	-0.168865
8	-2.072109	-1.629892	-0.437086
1	-1.098484	-1.509200	-0.396416
1	-2.242857	-2.376370	0.139754
8	-2.645264	0.781105	0.828173
1	-3.519886	1.051429	0.543100
1	-2.529693	-0.105888	0.433187
8	-0.855220	1.815023	-1.121840
1	-0.328050	1.030686	-1.293547
1	-1.474259	1.517025	-0.432041

Cartesian coordinates of : IMZW3_ 2

Atomic number (AN) and Cartesian coordinates

AN	X	Y	Z
6	2.334108	0.054280	-0.239946
6	1.368283	0.606624	-1.033240
6	0.766022	0.664216	1.067685
1	3.279554	-0.361667	-0.543424
1	1.300848	0.759177	-2.095108
1	-0.544686	1.361137	-0.428650
1	0.154346	0.861420	1.931876
7	1.955260	0.095152	1.081002
7	0.377536	1.005292	-0.177949
8	-2.275306	-0.988137	0.711637
1	-1.935750	-0.733668	1.572008
1	-1.512327	-1.415194	0.278307
8	-2.442157	1.468912	-0.613758
1	-2.483526	0.621996	-0.128662
1	-2.752544	2.118528	0.019331
8	-0.126292	-2.304464	-0.539466
1	0.562348	-1.671084	-0.773143
1	-0.458276	-2.604959	-1.388205

Cartesian coordinates of : IMZW3_ 3

Atomic number (AN) and Cartesian coordinates

AN	X	Y	Z
6	1.570920	-0.967500	0.440885
6	2.039678	0.268097	0.776382
6	0.938629	0.354325	-1.118263
1	1.686337	-1.891300	0.981262
1	2.615021	0.625666	1.610143
1	1.805971	2.085530	-0.298311
1	0.500387	0.772318	-2.007817
7	0.888387	-0.910835	-0.752874
7	1.630150	1.097912	-0.232607
8	-1.986043	1.964154	-0.385941
1	-2.876731	2.251550	-0.180764
1	-1.787864	1.311336	0.308165
8	-1.465683	-0.045277	1.555096
1	-0.510367	-0.080026	1.664530
1	-1.642107	-0.734278	0.887749
8	-1.801372	-1.817400	-0.610541
1	-0.854316	-1.655540	-0.791388
1	-1.836662	-2.736148	-0.338147

Cartesian coordinates of : IMZW3_ 4

Atomic number (AN) and Cartesian coordinates

AN	X	Y	Z
6	-2.076539	0.253314	-0.846760
6	-0.842410	-0.262316	-1.125290
6	-1.471306	-0.822276	0.891459
1	-2.703594	0.850871	-1.486082
1	-0.211192	-0.207498	-1.994119
1	0.434944	-1.398278	0.141524
1	-1.425424	-1.268872	1.869981
7	-2.471457	-0.102957	0.421292
7	-0.474739	-0.958469	-0.006964
8	2.277968	-1.749611	0.299997
1	2.465748	-0.826465	0.038918
1	2.533699	-2.267268	-0.465415
8	0.298069	2.231627	0.643738
1	-0.340571	1.911237	-0.003708
1	0.066553	1.747225	1.440117
8	2.551480	0.916721	-0.433225
1	1.799552	1.379594	-0.019076
1	3.325052	1.307205	-0.022978

Cartesian coordinates of : IMZW3_ 5

Atomic number (AN) and Cartesian coordinates

AN	X	Y	Z
6	-2.364000	-0.105298	-0.291390
6	-1.370526	-0.727712	-0.993176
6	-0.805373	-0.501579	1.106387
1	-3.312898	0.245150	-0.660105
1	-1.280430	-1.013082	-2.025601
1	0.542416	-1.368781	-0.266148
1	-0.202612	-0.566600	1.996540
7	-2.009341	0.031255	1.029416
7	-0.385690	-0.987233	-0.079410
8	2.362720	1.002621	0.539200
1	1.587142	1.408222	0.108722
1	3.106319	1.509024	0.207668
8	2.412232	-1.599767	-0.431163
1	2.686358	-2.171623	0.287822
1	2.506362	-0.696169	-0.070894
8	0.123808	2.245112	-0.645105
1	-0.482191	1.577093	-0.985304
1	-0.335952	2.592417	0.122875

Cartesian coordinates of : IMZW3_ 6

Atomic number (AN) and Cartesian coordinates

AN	X	Y	Z
6	2.039585	-0.243100	0.869374
6	0.790821	0.248496	1.124750
6	1.466420	0.861927	-0.861240
1	2.657909	-0.845356	1.512788
1	0.137379	0.166421	1.974898
1	-0.468218	1.392676	-0.152075
1	1.440693	1.329560	-1.830608
7	2.463754	0.146633	-0.379235
7	0.443845	0.964087	0.012133
8	-0.263731	-2.215029	-0.752344
1	-0.037980	-1.678447	-1.516346
1	0.374934	-1.932798	-0.087604
8	-2.318861	1.699219	-0.344803
1	-2.483459	0.776566	-0.068143
1	-2.611021	2.225312	0.401557
8	-2.546141	-0.986555	0.377462
1	-2.353843	-0.989124	1.317275
1	-1.760676	-1.404861	-0.021853

Cartesian coordinates of : IMZW3_ 7

Atomic number (AN) and Cartesian coordinates

```
  AN          X              Y             Z
 ----------------------------------------------------
  6     -2.056384      0.281922      0.834971
  6     -0.814273     -0.200723      1.135966
  6     -1.451014     -0.894788     -0.836156
  1     -2.685965      0.911369      1.440337
  1     -0.178126     -0.084161      1.995096
  1      0.467509     -1.398157     -0.069647
  1     -1.407125     -1.403086     -1.784170
  7     -2.456241     -0.158622     -0.404663
  7     -0.446659     -0.962642      0.061341
  8      2.321286     -1.742496     -0.151833
  1      2.485601     -0.789747     -0.010176
  1      2.553055     -1.887892     -1.070694
  8      2.547102      0.984695      0.390117
  1      1.768428      1.394744     -0.030310
  1      2.338173      1.003307      1.326253
  8      0.282741      2.186683     -0.802978
  1      0.063745      1.622864     -1.549094
  1     -0.363993      1.930097     -0.135480
 ----------------------------------------------------

Cartesian coordinates of :  IMZW3_ 8

 ----------------------------------------------------
 Atomic number (AN) and Cartesian coordinates
  AN          X              Y             Z
 ----------------------------------------------------
  6     -2.372410     -0.154088      0.281272
  6     -1.418441     -0.882513      0.931001
  6     -0.750692     -0.370795     -1.086572
  1     -3.334557      0.156131      0.651663
  1     -1.377013     -1.306858      1.917630
  1      0.525328     -1.434943      0.205840
  1     -0.108236     -0.320891     -1.949617
  7     -1.953340      0.165097     -0.989454
  7     -0.392953     -1.023594      0.037165
  8      2.411938      1.053558     -0.371150
  1      3.118016      1.516732      0.082942
  1      1.596526      1.456041     -0.018545
  8      0.043250      2.269557      0.577368
  1     -0.243013      1.736677      1.324125
  1     -0.580104      2.026828     -0.115281
  8      2.407572     -1.642577      0.319793
  1      2.495938     -0.701410      0.072058
  1      2.678342     -2.118758     -0.467076
 ----------------------------------------------------

Cartesian coordinates of :  IMZW3_ 9

 ----------------------------------------------------
 Atomic number (AN) and Cartesian coordinates
  AN          X              Y             Z
 ----------------------------------------------------
```

```
 6      2.372389     -0.154096      0.281307
 6      1.418400     -0.882518      0.931008
 6      0.750712     -0.370799     -1.086584
 1      3.334524      0.156124      0.651728
 1      1.376944     -1.306864      1.917635
 1     -0.525349     -1.434947      0.205794
 1      0.108282     -0.320895     -1.949650
 7      1.953358      0.165090     -0.989431
 7      0.392939     -1.023599      0.037140
 8     -2.407573     -1.642581      0.319783
 1     -2.495934     -0.701409      0.072070
 1     -2.678285     -2.118742     -0.467118
 8     -2.411946      1.053590     -0.371131
 1     -1.596520      1.456059     -0.018543
 1     -3.118004      1.516737      0.083021
 8     -0.043217      2.269555      0.577335
 1      0.580148      2.026795     -0.115293
 1      0.243007      1.736679      1.324110
---------------------------------------------------
```

```
Cartesian coordinates of :  IMZW3_10

 ---------------------------------------------------
 Atomic number (AN) and Cartesian coordinates
  AN          X             Y             Z
 ---------------------------------------------------
 6     -2.372407     -0.154048     -0.281235
 6     -1.418492     -0.882528     -0.930980
 6     -0.750655     -0.370800      1.086561
 1     -3.334551      0.156209     -0.651603
 1     -1.377115     -1.306896     -1.917601
 1      0.525271     -1.435037     -0.205869
 1     -0.108168     -0.320907      1.949585
 7     -1.953282      0.165143      0.989471
 7     -0.392981     -1.023636     -0.037174
 8      2.411935      1.053577      0.371155
 1      3.118015      1.516729     -0.082958
 1      1.596526      1.456048      0.018531
 8      0.043251      2.269533     -0.577399
 1     -0.243013      1.736640     -1.324147
 1     -0.580083      2.026790      0.115263
 8      2.407566     -1.642575     -0.319800
 1      2.678359     -2.118749      0.467064
 1      2.495911     -0.701407     -0.072066
 ---------------------------------------------------
```

```
Cartesian coordinates of :  IMZW3_11

 ---------------------------------------------------
 Atomic number (AN) and Cartesian coordinates
  AN          X             Y             Z
 ---------------------------------------------------
 6     -1.789230     -1.026232     -0.322924
 6     -2.810797     -0.164742     -0.062260
```

6	-0.882657	0.805425	0.330405
1	-1.845201	-2.042869	-0.671347
1	-3.878103	-0.263483	-0.130515
1	-2.685908	1.840780	0.624953
1	-0.177871	1.568808	0.612421
7	-0.582540	-0.414401	-0.074723
7	-2.214090	0.996016	0.352404
8	1.678225	0.055717	-1.896749
1	0.943315	-0.219120	-1.322160
1	2.298084	-0.674276	-1.836540
8	1.801958	-1.377410	1.260644
1	1.004884	-1.134220	0.752533
1	2.360678	-1.813375	0.613940
8	2.711926	1.257803	0.528594
1	2.428163	1.062087	-0.376922
1	2.487609	0.428785	0.978639

Cartesian coordinates of : IMZW3_12

Atomic number (AN) and Cartesian coordinates

AN	X	Y	Z
6	0.841933	-0.750437	0.022961
6	2.137532	-1.171987	0.049657
6	2.047398	1.016946	-0.067764
1	-0.053527	-1.348181	0.052186
1	2.583173	-2.147809	0.102853
1	3.895622	0.018004	-0.007261
1	2.389909	2.035577	-0.120820
7	0.791220	0.621918	-0.050742
7	2.891908	-0.031575	-0.008713
8	-2.557865	-0.930595	-1.328524
1	-2.452014	-0.974473	-0.364358
1	-1.661839	-1.019124	-1.659892
8	-2.379985	-0.492224	1.523169
1	-2.176895	0.384394	1.153122
1	-1.532304	-0.806065	1.845528
8	-1.795437	1.744056	-0.154227
1	-0.861405	1.436261	-0.142780
1	-2.207499	1.151990	-0.794860

Cartesian coordinates of : IMZW3_13

Atomic number (AN) and Cartesian coordinates

AN	X	Y	Z
6	-0.495499	1.356053	0.853261
6	0.748451	0.867128	1.128648
6	0.250722	0.534524	-0.973275
1	-1.182979	1.842855	1.523763

1	1.334558	0.846810	2.029177
1	2.118742	-0.099244	-0.200427
1	0.358039	0.206502	-1.993140
7	-0.802451	1.152608	-0.471832
7	1.212255	0.349376	-0.049772
8	-1.568309	-1.847430	1.006880
1	-2.141927	-1.428095	0.343139
1	-1.078381	-1.105695	1.373005
8	-3.095351	-0.453557	-0.971645
1	-3.865492	-0.085468	-0.534773
1	-2.413600	0.241134	-0.880707
8	3.748815	-0.902918	-0.521438
1	3.875914	-1.591976	0.133996
1	4.423212	-0.255700	-0.304986

Cartesian coordinates of : IMZW3_14

Atomic number (AN) and Cartesian coordinates

AN	X	Y	Z
6	-0.331909	-0.633605	-0.777921
6	0.965628	-1.043970	-0.691883
6	0.746639	0.958963	0.159159
1	-1.174970	-1.159294	-1.193557
1	1.460134	-1.948569	-0.994851
1	2.638353	0.009614	0.127917
1	1.018472	1.886399	0.633009
7	-0.464440	0.625600	-0.243278
7	1.640133	-0.016261	-0.091339
8	4.438918	0.126376	0.530745
1	4.918845	-0.217169	-0.225775
1	4.620666	-0.511795	1.223819
8	-3.154466	1.361499	-0.026558
1	-3.483441	1.422519	-0.924844
1	-2.186947	1.210455	-0.131340
8	-3.560395	-1.312903	0.795150
1	-2.664861	-1.655089	0.796142
1	-3.450716	-0.390543	0.500965

Cartesian coordinates of : IMZW3_15

Atomic number (AN) and Cartesian coordinates

AN	X	Y	Z
6	0.254907	-1.988369	-0.412224
6	1.521530	-1.491292	-0.501932
6	0.186412	-0.002801	0.381095
1	-0.104246	-2.957449	-0.712998
1	2.437919	-1.911314	-0.874006
1	2.238028	0.435178	0.092761

```
  1     -0.137858      0.924687      0.821672
  7     -0.580454     -1.050289      0.143993
  7      1.460809     -0.223566      0.009006
  8      3.672707      1.593390      0.223574
  1      3.401017      2.422230     -0.176271
  1      3.776211      1.811585      1.152176
  8     -2.638189      2.039394     -0.734369
  1     -3.410895      2.255868     -1.257610
  1     -2.902695      1.238981     -0.245375
  8     -3.240011     -0.334523      0.662645
  1     -3.259634     -0.103692      1.592826
  1     -2.333483     -0.690400      0.519396
 ---------------------------------------------------

Cartesian coordinates of :  IMZW3_16

 ---------------------------------------------------
 Atomic number (AN) and Cartesian coordinates
  AN         X             Y             Z
 ---------------------------------------------------
  6     -2.238099     -0.286126      1.009753
  6     -0.898188     -0.225524      0.759103
  6     -2.041223      0.234758     -1.048617
  1     -2.726923     -0.520309      1.939890
  1     -0.034458     -0.383968      1.379841
  1      0.086181      0.234868     -1.063623
  1     -2.234804      0.494895     -2.074964
  7     -2.954829      0.003254     -0.126904
  7     -0.789259      0.109729     -0.563264
  8      2.278600     -1.899301      0.313927
  1      2.346292     -1.400531      1.132083
  1      1.373829     -2.221618      0.323260
  8      1.976055      0.305769     -1.340842
  1      2.191474     -0.530664     -0.886159
  1      2.558450      0.346646     -2.100820
  8      2.060922      1.798854      1.070817
  1      1.132117      1.850056      1.303315
  1      2.056901      1.358516      0.205696
 ---------------------------------------------------

Cartesian coordinates of :  IMZW3_17

 ---------------------------------------------------
 Atomic number (AN) and Cartesian coordinates
  AN         X             Y             Z
 ---------------------------------------------------
  6      2.258724      0.007208      1.002007
  6      0.906452      0.012354      0.818465
  6      1.943083     -0.014648     -1.107038
  1      2.800596      0.014545      1.932198
  1      0.078289      0.023666      1.504936
  1     -0.190103     -0.003159     -0.993775
  1      2.078138     -0.027211     -2.174838
  7      2.908813     -0.009603     -0.209052
```

7	0.721419	-0.001917	-0.536897
8	-2.106208	-0.003907	-1.190207
1	-2.168019	-0.767131	-0.590054
1	-2.165053	0.764142	-0.596003
8	-2.045463	2.193627	0.593230
1	-2.281659	2.987884	0.109466
1	-1.097942	2.277875	0.722651
8	-2.079731	-2.198636	0.600402
1	-2.083977	-2.995909	0.066463
1	-1.180225	-2.152215	0.932601

Cartesian coordinates of : IMZW3_18

Atomic number (AN) and Cartesian coordinates

AN	X	Y	Z
6	1.108902	1.311961	0.482215
6	-0.215530	1.010160	0.604536
6	0.914009	-0.764990	0.009471
1	1.594483	2.259671	0.639873
1	-1.070317	1.601301	0.879166
1	-1.190224	-0.860371	0.288568
1	1.112927	-1.786079	-0.267672
7	1.814690	0.194118	0.108196
7	-0.322255	-0.319891	0.301212
8	-2.899459	-1.598995	0.196069
1	-3.276172	-0.746319	-0.091290
1	-3.202972	-1.699592	1.099993
8	4.508848	-0.451788	-0.472784
1	3.582696	-0.183631	-0.287593
1	4.642505	-0.202932	-1.388026
8	-3.681314	0.929897	-0.704050
1	-2.815636	1.293050	-0.905240
1	-4.093225	0.829616	-1.564841

Cartesian coordinates of : IMZW3_19

Atomic number (AN) and Cartesian coordinates

AN	X	Y	Z
6	2.319138	-1.066937	-0.040670
6	0.970433	-0.860148	-0.058227
6	2.039851	1.043654	0.061791
1	2.844514	-2.005861	-0.077859
1	0.134336	-1.534200	-0.111918
1	-0.081298	0.996213	0.017141
1	2.192092	2.107723	0.119287
7	2.990116	0.130213	0.034690
7	0.807987	0.497185	0.007845
8	-1.840718	1.771552	0.062399

1	-2.063441	1.281719	-0.740853
1	-2.075799	1.129341	0.746562
8	-2.263371	-0.763956	1.327712
1	-2.991527	-1.168631	1.803535
1	-1.477952	-1.172699	1.698009
8	-2.313491	-0.639002	-1.528977
1	-2.277840	-0.842436	-0.580404
1	-1.425704	-0.831120	-1.837678

Cartesian coordinates of : IMZW4_ 1

Atomic number (AN) and Cartesian coordinates

AN	X	Y	Z
6	-2.551716	-0.524757	0.209277
6	-1.562804	-1.052078	-0.570200
6	-1.395122	1.129304	-0.478616
1	-3.334610	-1.050778	0.728051
1	-1.314813	-2.060531	-0.847153
1	0.026180	-0.001257	-1.533058
1	-1.003335	2.113928	-0.668909
7	-2.446955	0.846117	0.262342
7	-0.840541	0.021638	-1.013692
8	0.567653	-0.695419	1.948273
1	0.982556	-1.183720	1.210408
1	-0.372890	-0.743501	1.753164
8	1.609845	1.740211	1.021885
1	2.442182	1.837358	1.487924
1	1.214105	0.934151	1.406843
8	1.972265	0.609623	-1.485935
1	1.950642	1.101891	-0.640808
1	2.791058	0.868677	-1.911215
8	1.845690	-1.899203	-0.238304
1	2.014548	-1.070482	-0.723756
1	2.711082	-2.176530	0.067845

Cartesian coordinates of : IMZW4_ 2

Atomic number (AN) and Cartesian coordinates

AN	X	Y	Z
6	1.519971	-1.848618	-0.323692
6	0.235680	-2.139032	-0.680638
6	0.262500	-0.636074	0.906208
1	2.430432	-2.261049	-0.723481
1	-0.178371	-2.812365	-1.408614
1	-1.568781	-1.251728	0.084335
1	-0.107845	0.073050	1.627711
7	1.534343	-0.908924	0.680023
7	-0.554840	-1.363389	0.122783

```
  8     -1.824965      1.991643      0.179761
  1     -1.026596      1.862511     -0.369880
  1     -1.486899      1.999949      1.077504
  8      2.543933      1.758659      0.467897
  1      2.359404      0.817447      0.653457
  1      2.177706      2.217541      1.226624
  8      0.445958      1.717355     -1.433134
  1      0.466693      0.804290     -1.729035
  1      1.182699      1.770579     -0.795286
  8     -3.258190     -0.405488      0.068154
  1     -2.817469      0.464633      0.130755
  1     -3.650286     -0.533675      0.933585
 ---------------------------------------------------

Cartesian coordinates of :  IMZW4_ 3

 ---------------------------------------------------
 Atomic number (AN) and Cartesian coordinates
  AN          X             Y             Z
 ---------------------------------------------------
  6      1.529740     -1.683685      0.317786
  6      0.263069     -1.965348      0.742572
  6      0.206164     -0.722504     -1.055610
  1      2.461763     -1.996533      0.756299
  1     -0.112531     -2.537495      1.571091
  1     -1.582811     -1.258018     -0.103648
  1     -0.205754     -0.148192     -1.868668
  7      1.491619     -0.908947     -0.817920
  7     -0.569484     -1.354909     -0.155795
  8      2.271385      1.869656     -0.557744
  1      1.617428      2.302615     -1.111652
  1      2.175475      0.928094     -0.788315
  8     -3.232707     -0.294666     -0.270163
  1     -2.732355      0.538508     -0.172515
  1     -3.730783     -0.367206      0.545789
  8     -1.594756      1.971960     -0.090806
  1     -1.174125      1.964113     -0.953541
  1     -0.879355      1.702349      0.516611
  8      0.541947      1.330663      1.633264
  1      1.204714      1.551171      0.953308
  1      0.622598      0.375906      1.726343
 ---------------------------------------------------

Cartesian coordinates of :  IMZW4_ 4

 ---------------------------------------------------
 Atomic number (AN) and Cartesian coordinates
  AN          X             Y             Z
 ---------------------------------------------------
  0      0.000000      0.000000      0.000000
  0      0.000000      0.000000      0.000000
  0      0.000000      0.000000      0.000000
  0      0.000000      0.000000      0.000000
  0      0.000000      0.000000      0.000000
```

```
  0      0.000000      0.000000      0.000000
  0      0.000000      0.000000      0.000000
  0      0.000000      0.000000      0.000000
  0      0.000000      0.000000      0.000000
  0      0.000000      0.000000      0.000000
  0      0.000000      0.000000      0.000000
  0      0.000000      0.000000      0.000000
  0      0.000000      0.000000      0.000000
  0      0.000000      0.000000      0.000000
  0      0.000000      0.000000      0.000000
  0      0.000000      0.000000      0.000000
  0      0.000000      0.000000      0.000000
  0      0.000000      0.000000      0.000000
  0      0.000000      0.000000      0.000000
  0      0.000000      0.000000      0.000000
  0      0.000000      0.000000      0.000000
 ---------------------------------------------------

Cartesian coordinates of :  IMZW4_ 5

 ---------------------------------------------------
 Atomic number (AN) and Cartesian coordinates
  AN          X             Y             Z
 ---------------------------------------------------
  0      0.000000      0.000000      0.000000
  0      0.000000      0.000000      0.000000
  0      0.000000      0.000000      0.000000
  0      0.000000      0.000000      0.000000
  0      0.000000      0.000000      0.000000
  0      0.000000      0.000000      0.000000
  0      0.000000      0.000000      0.000000
  0      0.000000      0.000000      0.000000
  0      0.000000      0.000000      0.000000
  0      0.000000      0.000000      0.000000
  0      0.000000      0.000000      0.000000
  0      0.000000      0.000000      0.000000
  0      0.000000      0.000000      0.000000
  0      0.000000      0.000000      0.000000
  0      0.000000      0.000000      0.000000
  0      0.000000      0.000000      0.000000
  0      0.000000      0.000000      0.000000
  0      0.000000      0.000000      0.000000
  0      0.000000      0.000000      0.000000
  0      0.000000      0.000000      0.000000
  0      0.000000      0.000000      0.000000
 ---------------------------------------------------

Cartesian coordinates of :  IMZW4_ 6

 ---------------------------------------------------
 Atomic number (AN) and Cartesian coordinates
  AN          X             Y             Z
 ---------------------------------------------------
  6     -2.478828      0.756872     -0.105645
```

6	-1.376292	1.041266	0.648774
6	-1.581170	-1.117953	0.366621
1	-3.189349	1.448971	-0.524073
1	-0.952766	1.966717	0.994775
1	0.083956	-0.325031	1.398462
1	-1.349926	-2.166691	0.442597
7	-2.608378	-0.601577	-0.277846
7	-0.820696	-0.171408	0.954701
8	0.556044	0.592893	-1.805781
1	-0.350245	0.822518	-1.569996
1	0.646594	0.866143	-2.721231
8	2.000937	-0.620920	1.600781
1	1.945542	-1.176011	0.803467
1	2.141999	0.265964	1.224921
8	2.093648	1.845301	0.220025
1	2.977236	1.992812	-0.121851
1	1.604807	1.474954	-0.535231
8	1.538683	-1.906078	-0.885780
1	2.368096	-2.039911	-1.347951
1	1.180827	-1.090217	-1.274338

Cartesian coordinates of : IMZW4_ 7

Atomic number (AN) and Cartesian coordinates

AN	X	Y	Z
6	2.467287	-0.660781	-0.132873
6	1.377806	-0.779124	-0.947670
6	1.439070	1.203384	-0.026233
1	3.226893	-1.397266	0.065852
1	1.012095	-1.585606	-1.557318
1	-0.170650	0.665595	-1.273064
1	1.139687	2.210972	0.208257
7	2.506717	0.588235	0.442184
7	0.738608	0.427990	-0.879670
8	-1.664375	1.318461	1.446297
1	-0.996357	1.995722	1.571254
1	-1.179314	0.481953	1.582011
8	-0.490244	-1.219144	1.739684
1	0.435468	-1.216715	1.479135
1	-0.954693	-1.522749	0.936826
8	-1.959771	1.401960	-1.337402
1	-2.580117	0.717203	-1.594060
1	-1.957729	1.368088	-0.360944
8	-1.999892	-1.894735	-0.518853
1	-1.872078	-2.823883	-0.722410
1	-1.611216	-1.440093	-1.270284

Cartesian coordinates of : IMZW4_ 8

Atomic number (AN) and Cartesian coordinates

AN	X	Y	Z
6	-2.000632	-0.524167	0.992750
6	-2.700533	-0.431627	-0.174508
6	-0.620356	-0.998943	-0.574138
1	-2.354681	-0.359284	1.995591
1	-3.724147	-0.186625	-0.389175
1	-1.994734	-0.765906	-2.148730
1	0.260544	-1.265779	-1.133014
7	-0.699316	-0.886129	0.736585
7	-1.805359	-0.742999	-1.161768
8	1.922754	-0.399297	1.544362
1	2.044942	-0.175856	2.468179
1	0.970786	-0.624693	1.449379
8	3.152555	-1.072090	-0.952345
1	2.793125	-0.232579	-1.258792
1	2.883563	-1.071062	-0.023854
8	-0.953577	2.540812	-0.009946
1	-1.485715	1.798551	0.291182
1	-0.078681	2.150110	-0.179954
8	1.643400	1.508677	-0.511614
1	2.232184	2.260161	-0.416285
1	1.793613	0.980466	0.293475

Cartesian coordinates of : IMZW4_ 9

Atomic number (AN) and Cartesian coordinates

AN	X	Y	Z
6	-2.519729	-0.549258	0.040385
6	-2.114335	0.706430	0.396613
6	-0.805779	-0.116608	-1.149816
1	-3.356797	-1.106537	0.424866
1	-2.495778	1.417829	1.106341
1	-0.401432	1.777733	-0.338319
1	0.003016	-0.160079	-1.859667
7	-1.699536	-1.062724	-0.936321
7	-1.024746	0.972620	-0.385777
8	0.137360	-1.055045	2.078951
1	-0.721921	-0.750138	1.764006
1	0.292087	-1.852397	1.566319
8	2.226737	0.335331	0.724241
1	2.328047	-0.347051	0.038602
1	1.487643	-0.011830	1.251798
8	1.276782	2.689427	-0.382925
1	1.316903	3.350387	0.310183
1	1.678040	1.892883	0.020573
8	2.276878	-1.861967	-1.073138
1	1.390568	-2.178150	-0.880997
1	2.846593	-2.507269	-0.649141

Cartesian coordinates of : IMZW4_10

Atomic number (AN) and Cartesian coordinates

AN	X	Y	Z
6	-2.499659	0.067157	0.486115
6	-1.511724	-0.642250	1.104215
6	-1.220514	-0.653706	-1.062946
1	-3.330371	0.579881	0.939779
1	-1.310820	-0.856029	2.138247
1	0.184561	-1.588962	0.196610
1	-0.763798	-0.869978	-2.014105
7	-2.317013	0.059175	-0.878764
7	-0.709711	-1.107104	0.098959
8	0.084863	2.114383	-0.352723
1	0.060857	3.028266	-0.642995
1	-0.793767	1.769840	-0.548505
8	2.189318	0.438564	-1.312195
1	2.960755	0.870446	-0.939312
1	1.450249	1.004502	-1.030957
8	1.534256	0.996877	1.848806
1	2.023884	0.363019	1.319324
1	0.978686	1.442547	1.192326
8	2.012472	-2.040713	-0.022638
1	2.398064	-1.931915	0.848849
1	2.152886	-1.176213	-0.454935

Cartesian coordinates of : IMZW4_11

Atomic number (AN) and Cartesian coordinates

AN	X	Y	Z
6	2.022374	-0.095504	-1.179231
6	2.708489	-0.889801	-0.308831
6	1.575885	0.637848	0.783109
1	1.997241	-0.151322	-2.253763
1	3.362365	-1.729602	-0.453782
1	2.757752	-0.767557	1.809230
1	1.185131	1.197362	1.615760
7	1.319978	0.865333	-0.489585
7	2.415713	-0.404692	0.936299
8	-3.502960	-0.962343	-0.589785
1	-4.071622	-1.686135	-0.321522
1	-2.611691	-1.347052	-0.581360
8	-0.789866	-1.671804	-0.041733
1	-0.945100	-0.926678	0.562669
1	-0.114167	-1.344905	-0.643330
8	-1.193920	2.101857	-0.713937
1	-1.096813	3.024416	-0.471918
1	-0.275192	1.755325	-0.728343

8	-1.981343	0.488514	1.443901
1	-1.705484	1.136694	0.768692
1	-2.728023	0.049910	1.012822

Cartesian coordinates of : IMZW4_12

Atomic number (AN) and Cartesian coordinates

AN	X	Y	Z
6	2.336455	1.087362	0.653383
6	1.074255	0.611071	0.447597
6	2.561512	-0.788233	-0.335944
1	2.626819	2.009210	1.127599
1	0.100729	0.998859	0.692133
1	0.472118	-1.191943	-0.491324
1	2.960129	-1.667647	-0.811720
7	3.270080	0.207003	0.160239
7	1.236232	-0.591313	-0.184308
8	-1.929633	-1.034811	1.766364
1	-2.756686	-1.430976	2.046009
1	-2.185660	-0.149757	1.440539
8	-2.571229	1.427070	0.626446
1	-2.001599	2.085167	1.029568
1	-2.195202	1.299860	-0.262997
8	-1.315122	-1.805447	-0.882994
1	-1.487074	-0.963927	-1.332005
1	-1.587153	-1.629577	0.035864
8	-1.519309	0.849636	-1.910311
1	-2.007668	1.174598	-2.669654
1	-0.633938	1.203511	-2.021782

Cartesian coordinates of : IMZW4_13

Atomic number (AN) and Cartesian coordinates

AN	X	Y	Z
6	0.137012	-0.486824	1.171117
6	-1.120529	0.031650	1.284448
6	-0.903768	-1.025171	-0.618127
1	0.954155	-0.420961	1.870015
1	-1.602071	0.599721	2.059156
1	-2.737525	-0.095556	-0.112892
1	-1.164374	-1.423052	-1.583770
7	0.266727	-1.152751	-0.023506
7	-1.771435	-0.323268	0.133980
8	2.980846	-1.579369	-0.550481
1	3.309832	-2.062353	0.209557
1	2.007469	-1.540547	-0.411318
8	0.836943	2.353017	-0.375174
1	0.262792	1.844173	0.204477

1	1.702537	1.918751	-0.263217
8	3.343742	1.129291	0.006373
1	3.916868	1.473733	-0.680677
1	3.263059	0.177875	-0.204020
8	-4.467918	0.282758	-0.622245
1	-4.557674	1.237683	-0.648772
1	-5.047310	0.019158	0.095738

Cartesian coordinates of : IMZW4_14

Atomic number (AN) and Cartesian coordinates

AN	X	Y	Z
6	-1.961151	-0.372867	0.758918
6	-0.835238	0.285187	1.161492
6	-1.635458	1.184615	-0.663755
1	-2.437355	-1.218381	1.225616
1	-0.168717	0.144259	1.993671
1	0.163880	1.901265	0.216907
1	-1.714321	1.855485	-1.502014
7	-2.464821	0.194410	-0.388317
7	-0.648334	1.286198	0.249545
8	0.165364	-1.412736	-1.707499
1	0.158821	-1.982092	-0.920765
1	-0.651084	-0.908607	-1.634297
8	2.274928	-0.305408	-0.129869
1	3.100283	-0.587593	-0.530092
1	1.601178	-0.503146	-0.804266
8	2.002370	2.449162	0.280430
1	2.227635	1.519460	0.092257
1	2.191322	2.906329	-0.540905
8	0.848646	-2.696956	0.773473
1	0.147529	-2.419910	1.368085
1	1.433534	-1.925422	0.734995

Cartesian coordinates of : IMZW4_15

Atomic number (AN) and Cartesian coordinates

AN	X	Y	Z
0	0.000000	0.000000	0.000000
0	0.000000	0.000000	0.000000
0	0.000000	0.000000	0.000000
0	0.000000	0.000000	0.000000
0	0.000000	0.000000	0.000000
0	0.000000	0.000000	0.000000
0	0.000000	0.000000	0.000000
0	0.000000	0.000000	0.000000
0	0.000000	0.000000	0.000000
0	0.000000	0.000000	0.000000

0	0.000000	0.000000	0.000000
0	0.000000	0.000000	0.000000
0	0.000000	0.000000	0.000000
0	0.000000	0.000000	0.000000
0	0.000000	0.000000	0.000000
0	0.000000	0.000000	0.000000
0	0.000000	0.000000	0.000000
0	0.000000	0.000000	0.000000
0	0.000000	0.000000	0.000000
0	0.000000	0.000000	0.000000
0	0.000000	0.000000	0.000000

Cartesian coordinates of : IMZW4_16

Atomic number (AN) and Cartesian coordinates

AN	X	Y	Z
6	2.310266	-1.307382	-0.092555
6	1.470679	-1.104332	-1.149403
6	0.435682	-0.720434	0.739167
1	3.341861	-1.614293	-0.117211
1	1.607985	-1.188260	-2.212072
1	-0.571571	-0.433282	-1.077939
1	-0.360982	-0.448509	1.411245
7	1.658316	-1.068644	1.095215
7	0.272962	-0.740483	-0.599073
8	-2.249773	0.557504	-1.223548
1	-1.831728	1.247223	-0.677316
1	-2.645342	-0.046589	-0.572357
8	-3.171246	-1.302471	0.713711
1	-3.783737	-1.898212	0.277192
1	-2.374602	-1.828519	0.814469
8	-0.892135	2.450336	0.384107
1	-1.087592	2.133495	1.268298
1	0.065624	2.294143	0.288605
8	1.905512	2.073497	0.139865
1	2.036280	1.338841	0.748625
1	2.046226	1.669825	-0.720865

Cartesian coordinates of : IMZW4_17

Atomic number (AN) and Cartesian coordinates

AN	X	Y	Z
6	-1.405076	-1.191943	0.960478
6	-0.113822	-1.635105	0.990908
6	-0.465792	-0.440888	-0.807366
1	-2.186859	-1.368853	1.679150
1	0.427056	-2.239713	1.695781
1	1.450932	-1.224634	-0.419238

```
  1     -0.262895      0.058808     -1.739703
  7     -1.622948     -0.447319     -0.173303
  7      0.466820     -1.157327     -0.152613
  8      2.298379      1.746815     -0.406210
  1      2.998068      2.314654     -0.078776
  1      1.684942      1.663723      0.347603
  8     -3.924902      0.925936     -1.092485
  1     -3.802575      1.834606     -0.815422
  1     -3.125884      0.466178     -0.755400
  8      0.481142      1.586159      1.744065
  1     -0.300567      1.995825      1.366165
  1      0.212264      0.670169      1.874911
  8      3.225277     -0.824039     -0.887741
  1      2.994572      0.117192     -0.758994
  1      3.272800     -0.926778     -1.839818
 ---------------------------------------------------

Cartesian coordinates of :  IMZW4_18

 ---------------------------------------------------
 Atomic number (AN) and Cartesian coordinates
  AN          X             Y             Z
 ---------------------------------------------------
  6     -1.221938      0.053947      1.315679
  6      0.075215     -0.372734      1.322638
  6     -0.917723     -0.995238     -0.523446
  1     -1.745171      0.610983      2.073869
  1      0.869550     -0.265863      2.039340
  1      1.137528     -1.429419     -0.186336
  1     -1.050738     -1.444195     -1.492836
  7     -1.841843     -0.342404      0.154651
  7      0.248905     -1.048202      0.146010
  8     -4.505924      0.134061     -0.684821
  1     -3.575391     -0.008558     -0.406816
  1     -4.463711      0.910118     -1.244582
  8      3.213575      1.033105     -0.056866
  1      2.353564      1.468456     -0.206186
  1      3.797769      1.439493     -0.699421
  8      2.931424     -1.666751     -0.647106
  1      3.108500     -0.726351     -0.446499
  1      3.339390     -2.143054      0.078101
  8      0.707550      2.277627     -0.421708
  1      0.808520      3.162832     -0.065047
  1      0.184432      1.819609      0.246569
 ---------------------------------------------------

Cartesian coordinates of :  IMZW4_19

 ---------------------------------------------------
 Atomic number (AN) and Cartesian coordinates
  AN          X             Y             Z
 ---------------------------------------------------
  6      0.017923     -0.003289      0.771514
  6     -1.286006     -0.007685      1.170375
```

```
  6     -1.163049     -0.005645     -1.013687
  1      0.905746     -0.001254      1.381645
  1     -1.745008     -0.010014      2.141860
  1     -3.046154     -0.011912     -0.047289
  1     -1.487318     -0.005915     -2.039914
  7      0.089638     -0.001849     -0.601652
  7     -2.025799     -0.009325      0.019598
  8      2.812364      0.008574     -1.056798
  1      1.827007      0.005343     -1.097767
  1      3.105039      0.013239     -1.969623
  8     -4.882041     -0.035337     -0.228100
  1     -5.235842     -0.508762      0.527829
  1     -5.190758      0.863774     -0.097815
  8      2.870572     -2.345192      0.516483
  1      1.965176     -2.350439      0.831739
  1      2.903461     -1.564031     -0.060973
  8      2.860017      2.350241      0.534876
  1      2.896114      1.573219     -0.047973
  1      1.955175      2.348392      0.851758
 ---------------------------------------------------

Cartesian coordinates of :  IMZW4_20

 ---------------------------------------------------
 Atomic number (AN) and Cartesian coordinates
  AN          X             Y             Z
 ---------------------------------------------------
  6      1.992016     -1.662401     -0.030116
  6      0.721785     -2.158367     -0.042956
  6      0.682594      0.024264      0.098305
  1      2.919831     -2.205886     -0.079605
  1      0.336786     -3.159787     -0.102673
  1     -1.122520     -1.077242      0.049252
  1      0.288595      1.024389      0.168599
  7      1.962840     -0.291963      0.059225
  7     -0.102095     -1.069495      0.040273
  8     -2.306917      1.543541      1.301065
  1     -2.054169      1.642332      0.367333
  1     -1.481650      1.335030      1.743943
  8     -2.056411      1.348386     -1.530858
  1     -1.160542      1.190710     -1.837285
  1     -2.406629      0.462802     -1.377184
  8     -2.999205     -0.888009      0.056526
  1     -2.972349     -0.172877      0.713537
  1     -3.500565     -1.594888      0.466377
  8      3.712052      1.935528      0.139218
  1      3.147283      1.135126      0.084620
  1      3.806189      2.213957     -0.772410
 ---------------------------------------------------

Cartesian coordinates of :  IMZW4_21

 ---------------------------------------------------
 Atomic number (AN) and Cartesian coordinates
```

AN	X	Y	Z
0	0.000000	0.000000	0.000000
0	0.000000	0.000000	0.000000
0	0.000000	0.000000	0.000000
0	0.000000	0.000000	0.000000
0	0.000000	0.000000	0.000000
0	0.000000	0.000000	0.000000
0	0.000000	0.000000	0.000000
0	0.000000	0.000000	0.000000
0	0.000000	0.000000	0.000000
0	0.000000	0.000000	0.000000
0	0.000000	0.000000	0.000000
0	0.000000	0.000000	0.000000
0	0.000000	0.000000	0.000000
0	0.000000	0.000000	0.000000
0	0.000000	0.000000	0.000000
0	0.000000	0.000000	0.000000
0	0.000000	0.000000	0.000000
0	0.000000	0.000000	0.000000
0	0.000000	0.000000	0.000000
0	0.000000	0.000000	0.000000
0	0.000000	0.000000	0.000000

Cartesian coordinates of : IMZW4_22

Atomic number (AN) and Cartesian coordinates

AN	X	Y	Z
6	0.415521	0.729013	1.236293
6	-0.652130	-0.106074	1.390066
6	-0.231306	0.196157	-0.732993
1	1.006942	1.203837	2.000403
1	-1.154355	-0.484004	2.261675
1	-1.829444	-1.052763	-0.127395
1	-0.323985	0.101272	-1.801682
7	0.675822	0.923104	-0.101798
7	-1.054201	-0.433257	0.123659
8	3.200478	0.034277	-1.088716
1	3.928885	0.428588	-0.605573
1	2.415308	0.528896	-0.785695
8	-1.411846	3.301996	-0.118803
1	-1.536867	3.684089	-0.989125
1	-0.671603	2.698174	-0.241766
8	2.181637	-2.118603	0.498890
1	2.587264	-1.419202	-0.041054
1	1.573457	-1.636741	1.065876
8	-3.206077	-2.193899	-0.543657
1	-2.844432	-2.873702	-1.116350
1	-3.808571	-1.712111	-1.114245

Cartesian coordinates of : IMZW4_23

Atomic number (AN) and Cartesian coordinates

AN	X	Y	Z
6	-3.720094	-0.832301	0.249791
6	-3.127689	0.258707	0.816319
6	-1.968505	-0.441302	-0.899061
1	-4.626772	-1.325734	0.555714
1	-3.390203	0.871044	1.659679
1	-1.314433	1.233441	0.212769
1	-1.183293	-0.473541	-1.635174
7	-2.990822	-1.271401	-0.829406
7	-2.008274	0.497821	0.068242
8	3.737361	-1.458745	1.216256
1	2.878020	-1.054350	1.025817
1	4.037036	-1.003993	2.005793
8	1.630245	0.064368	-0.067818
1	1.052799	-0.485292	-0.602061
1	2.428381	0.189000	-0.612512
8	0.155851	2.381627	0.389799
1	0.173172	2.933531	-0.393881
1	0.757209	1.643296	0.177778
8	4.264852	0.223779	-1.030428
1	4.356757	-0.417710	-0.306943
1	4.416258	-0.293485	-1.823589

Cartesian coordinates of : IMZW5_ 1

Atomic number (AN) and Cartesian coordinates

AN	X	Y	Z
6	2.109219	1.466920	-0.110079
6	2.504158	0.200677	0.213133
6	0.741084	0.142515	-1.079989
1	2.554533	2.399005	0.192764
1	3.311102	-0.174273	0.815633
1	1.539796	-1.642704	-0.342430
1	-0.068442	-0.270604	-1.657938
7	1.006212	1.427658	-0.931134
7	1.626534	-0.631530	-0.426377
8	0.433950	-3.227962	-0.287778
1	-0.278847	-2.703156	0.130379
1	0.715848	-3.832866	0.400136
8	-1.509608	-1.597958	0.843790
1	-1.045777	-0.883066	1.314088
1	-1.984300	-1.126800	0.135696
8	-0.340777	0.659368	2.133758
1	-0.780481	1.277137	1.525552
1	0.589357	0.734210	1.897313
8	-1.540252	2.169642	0.044990

1	-0.680585	2.160595	-0.418850
1	-1.829715	3.083764	0.034758
8	-2.838623	-0.032396	-1.116267
1	-2.475724	0.797522	-0.758649
1	-3.760271	-0.017894	-0.852199

Cartesian coordinates of : IMZW5_ 2

Atomic number (AN) and Cartesian coordinates

AN	X	Y	Z
6	-1.976054	-1.189950	0.488835
6	-2.803433	-0.416406	-0.271508
6	-1.394799	0.839089	0.846954
1	-1.962983	-2.262647	0.577045
1	-3.609418	-0.655049	-0.940380
1	-2.824170	1.700586	-0.433729
1	-0.907908	1.729736	1.204897
7	-1.100120	-0.397266	1.193369
7	-2.421655	0.874594	-0.025524
8	0.840062	1.856861	-1.449789
1	1.322288	1.795811	-0.600223
1	1.520882	2.057533	-2.094469
8	2.121434	1.697035	1.005092
1	3.066160	1.762873	0.855194
1	1.991901	0.781876	1.327937
8	1.612988	-0.920752	1.819817
1	1.906518	-1.286355	0.965220
1	0.648330	-0.803794	1.693803
8	2.398684	-1.706926	-0.758411
1	1.584775	-1.453750	-1.236833
1	2.429498	-2.662338	-0.831104
8	0.133840	-0.756141	-2.073402
1	-0.645498	-0.978444	-1.553524
1	0.321706	0.175660	-1.840894

Cartesian coordinates of : IMZW5_ 3

Atomic number (AN) and Cartesian coordinates

AN	X	Y	Z
6	-0.696827	-2.134798	0.723066
6	0.667990	-2.095137	0.733949
6	-0.114360	-0.820751	-0.861956
1	-1.352658	-2.689706	1.371619
1	1.400106	-2.576300	1.356282
1	1.965184	-0.948645	-0.529950
1	-0.119031	-0.134691	-1.693030
7	-1.183090	-1.339828	-0.286624
7	1.022226	-1.263765	-0.292844

8	3.521833	0.041018	-0.843306
1	3.604452	0.122104	-1.795137
1	3.010253	0.830512	-0.578411
8	-3.399880	0.206403	-1.003971
1	-4.114336	0.097793	-0.373900
1	-2.724762	-0.451933	-0.723744
8	-0.041472	1.037674	1.874697
1	-0.111046	0.078316	1.835996
1	-0.738348	1.358222	1.269028
8	-1.752198	2.223061	0.019029
1	-2.360007	1.561486	-0.365584
1	-1.078451	2.338775	-0.654607
8	1.809471	2.057135	-0.014597
1	2.258820	2.769055	0.444416
1	1.243012	1.651937	0.668124

Cartesian coordinates of : IMZW5_ 4

Atomic number (AN) and Cartesian coordinates

AN	X	Y	Z
6	2.920675	-0.769779	-0.202543
6	2.115804	-0.184432	-1.138032
6	1.894240	0.806560	0.802148
1	3.594734	-1.598219	-0.336653
1	1.944354	-0.386650	-2.179702
1	0.739691	1.420640	-0.841059
1	1.530245	1.506701	1.534750
7	2.783036	-0.142234	1.014438
7	1.477358	0.830795	-0.481213
8	-1.104389	2.148859	-0.432072
1	-1.849067	2.744653	-0.529390
1	-1.266900	1.670273	0.400758
8	-0.254071	-1.933855	0.296589
1	0.684379	-1.814954	0.115549
1	-0.478802	-1.202060	0.894998
8	-3.599513	-0.630083	0.182005
1	-3.720676	-1.562330	0.372729
1	-2.996838	-0.621197	-0.584884
8	-1.542870	-0.381173	-1.747147
1	-1.390646	0.520663	-1.420201
1	-1.001796	-0.931688	-1.155102
8	-1.420229	0.287649	1.622508
1	-1.550455	0.437005	2.560699
1	-2.276725	-0.048040	1.290431

Cartesian coordinates of : IMZW5_ 5

Atomic number (AN) and Cartesian coordinates

AN X Y Z

6	-2.998992	-0.663029	0.180892
6	-2.162722	-0.166739	1.140485
6	-1.876955	0.889066	-0.757117
1	-3.722860	-1.453431	0.281100
1	-2.008554	-0.420398	2.173586
1	-0.694461	1.366797	0.910187
1	-1.468136	1.594571	-1.460323
7	-2.820693	0.005102	-1.008631
7	-1.461748	0.833515	0.525867
8	0.076317	-1.887193	-0.344296
1	-0.850483	-1.850210	-0.080815
1	0.233267	-2.809598	-0.556350
8	1.594003	-0.495046	1.634407
1	1.051440	-1.019243	1.022367
1	1.423039	0.420024	1.359344
8	3.776299	-0.424034	-0.160800
1	3.123590	-0.545240	0.553172
1	4.094604	-1.309993	-0.342784
8	1.143087	2.087617	0.390526
1	1.964543	2.567636	0.509617
1	1.279857	1.550452	-0.415012
8	1.479655	0.237732	-1.682990
1	2.343167	-0.025000	-1.318173
1	0.885200	-0.445082	-1.336907

Cartesian coordinates of : IMZW5_ 6

Atomic number (AN) and Cartesian coordinates

AN	X	Y	Z
6	1.458199	2.018635	-0.150907
6	0.166954	2.375298	0.115949
6	0.219127	0.530114	-1.054191
1	2.361724	2.527471	0.138121
1	-0.257520	3.204146	0.652296
1	-1.624901	1.308528	-0.418733
1	-0.139737	-0.333561	-1.588636
7	1.486586	0.860723	-0.891968
7	-0.610575	1.418277	-0.476764
8	2.497509	-1.794343	-0.982651
1	2.354992	-0.827258	-0.976739
1	3.301030	-1.918346	-0.474514
8	-3.304805	0.484108	-0.327321
1	-2.906942	-0.408838	-0.299505
1	-3.632770	0.623587	0.562727
8	0.375066	-2.186391	0.876908
1	0.445805	-1.387765	1.429110
1	1.102197	-2.093993	0.235809
8	-2.075899	-2.012040	-0.410662
1	-1.878413	-2.076331	-1.347044
1	-1.194962	-2.037014	0.017846

```
  8      0.557815      0.104945      2.528531
  1      0.827480      0.836199      1.961028
  1     -0.353222      0.315657      2.745813
 ---------------------------------------------------

Cartesian coordinates of :  IMZW5_ 7

 ---------------------------------------------------
 Atomic number (AN) and Cartesian coordinates
  AN         X             Y             Z
 ---------------------------------------------------
  6     -2.867743     -0.086610      0.258019
  6     -1.883046     -0.297867      1.180882
  6     -1.294357     -1.094148     -0.770267
  1     -3.820350      0.392414      0.406270
  1     -1.801187     -0.057200      2.225518
  1      0.017159     -1.206516      0.893787
  1     -0.688823     -1.578939     -1.516245
  7     -2.496737     -0.591029     -0.966123
  7     -0.887984     -0.953742      0.508894
  8      1.914714      1.612285      1.077693
  1      1.022275      1.959605      1.192612
  1      1.938674      1.455226      0.118257
  8      1.337722      0.974312     -1.627754
  1      1.847996      1.322789     -2.360544
  1      1.615139      0.040145     -1.542120
  8      1.789685     -1.100297      1.598803
  1      2.323538     -1.394882      2.337877
  1      1.895265     -0.128605      1.560191
  8      2.124079     -1.642130     -1.111136
  1      3.048561     -1.719070     -1.353928
  1      2.136643     -1.542839     -0.141769
  8     -0.678920      2.455529     -0.191127
  1     -1.342562      1.848831      0.154275
  1     -0.126629      1.896613     -0.757203
 ---------------------------------------------------

Cartesian coordinates of :  IMZW5_ 8

 ---------------------------------------------------
 Atomic number (AN) and Cartesian coordinates
  AN         X             Y             Z
 ---------------------------------------------------
  6     -0.665642     -2.186413      0.784231
  6      0.696694     -2.101839      0.736356
  6     -0.196591     -0.875837     -0.838675
  1     -1.273450     -2.755094      1.466848
  1      1.470521     -2.550376      1.332169
  1      1.900479     -0.934004     -0.597345
  1     -0.258828     -0.203219     -1.678484
  7     -1.222433     -1.421398     -0.212274
  7      0.978678     -1.272726     -0.314140
  8     -3.449222      0.156970     -1.081282
  1     -2.808204     -0.511667     -0.775164
```

1	-3.037576	0.976343	-0.774691
8	-0.016378	0.999115	1.915533
1	-0.718031	1.329549	1.327501
1	-0.091120	0.040473	1.864058
8	-1.729774	2.184673	0.007861
1	-1.963697	3.077444	0.271720
1	-1.052477	2.308153	-0.662095
8	3.436849	0.058822	-0.985771
1	3.462734	0.172740	-1.937360
1	2.953146	0.843645	-0.661258
8	1.824000	2.063334	0.040026
1	2.328636	2.712020	0.534025
1	1.263590	1.634087	0.712566

Cartesian coordinates of : IMZW5_ 9

Atomic number (AN) and Cartesian coordinates

AN	X	Y	Z
6	1.529890	-1.795365	-0.483001
6	0.175137	-1.930723	-0.385023
6	0.935217	-0.672092	1.234556
1	2.187974	-2.218915	-1.222187
1	-0.544088	-2.465876	-0.977719
1	-1.135248	-1.007383	1.031130
1	0.927988	-0.046019	2.111028
7	2.006517	-1.011161	0.541377
7	-0.185305	-1.219181	0.727678
8	-2.813390	-0.301614	-1.551812
1	-2.080192	0.303212	-1.689815
1	-3.562579	0.159078	-1.935430
8	2.968846	1.558612	-0.348228
1	2.833184	0.680366	0.052001
1	2.871070	2.165721	0.388255
8	0.376910	1.351400	-1.448871
1	0.299235	0.398801	-1.560097
1	1.287983	1.470035	-1.119238
8	-0.959112	2.145598	0.902445
1	-0.308960	1.990727	1.591026
1	-0.516771	1.827793	0.091576
8	-2.781122	0.006098	1.269535
1	-2.962629	-0.141549	0.326206
1	-2.223976	0.804743	1.246122

Cartesian coordinates of : IMZW5_10

Atomic number (AN) and Cartesian coordinates

AN	X	Y	Z
6	2.085856	-1.750212	-0.018105

```
  6      0.918024     -2.219379     -0.543750
  6      0.526280     -0.535548      0.795817
  1      3.085526     -2.109008     -0.193457
  1      0.701447     -3.019197     -1.227899
  1     -1.062719     -1.465033     -0.234182
  1     -0.022896      0.217735      1.334611
  7      1.836084     -0.696980      0.829198
  7     -0.069048     -1.438597     -0.005794
  8      2.599231      2.050108      0.679235
  1      2.486408      1.097406      0.866234
  1      2.036721      2.478831      1.327395
  8     -2.875448     -1.062838     -0.759498
  1     -2.540023     -0.191322     -1.026597
  1     -3.136349     -0.892889      0.154078
  8     -1.677880      1.502299     -0.790831
  1     -0.738242      1.569209     -1.057493
  1     -2.097881      2.276244     -1.169556
  8     -2.671832      0.541709      1.708524
  1     -3.480385      1.026198      1.883447
  1     -2.293045      0.989748      0.934661
  8      0.975267      1.646428     -1.604268
  1      1.194786      0.732994     -1.802999
  1      1.521730      1.847317     -0.821140
 ---------------------------------------------------

Cartesian coordinates of :  IMZW5_11

 ---------------------------------------------------
 Atomic number (AN) and Cartesian coordinates
  AN          X             Y             Z
 ---------------------------------------------------
  6     -1.467800      0.798245      1.071899
  6     -1.068198     -0.446267      1.465866
  6     -0.535893     -0.105536     -0.627382
  1     -1.976296      1.548655      1.652639
  1     -1.148224     -0.969564      2.401026
  1      0.018683     -1.896681      0.305095
  1     -0.131090     -0.297712     -1.607368
  7     -1.138008      1.009107     -0.248000
  7     -0.484984     -1.011763      0.364662
  8      1.441870     -3.019377     -0.295550
  1      1.997788     -2.223627     -0.404852
  1      1.825006     -3.474084      0.456365
  8     -3.869295     -0.534181     -0.915808
  1     -3.105808      0.048066     -0.985192
  1     -3.840246     -0.816789      0.000835
  8      1.951557      1.264476      1.258823
  1      1.651148      1.878785      0.564059
  1      1.134003      0.968057      1.670830
  8      1.022618      2.818649     -0.934433
  1      1.462409      2.312746     -1.621964
  1      0.169551      2.361919     -0.829505
  8      2.810204     -0.607452     -0.669786
  1      2.496122     -0.007675      0.033439
```

```
  1      2.383613     -0.269077     -1.460305
 ---------------------------------------------------

Cartesian coordinates of :  IMZW5_12

 ---------------------------------------------------
 Atomic number (AN) and Cartesian coordinates
  AN          X             Y             Z
 ---------------------------------------------------
  6     -2.734945     -0.902747     -0.454683
  6     -2.164565      0.256219     -0.895715
  6     -1.692509     -0.076463      1.213236
  1     -3.339245     -1.597477     -1.012598
  1     -2.163579      0.747772     -1.851998
  1     -0.926602      1.614567      0.181137
  1     -1.277690      0.102729      2.190403
  7     -2.439655     -1.109860      0.871936
  7     -1.512921      0.779781      0.186125
  8      0.410640      2.933847     -0.121328
  1      0.084829      3.540279     -0.788788
  1      0.886386      2.251993     -0.626449
  8      1.850713      0.288101      1.662039
  1      2.383459      1.008005      2.006947
  1      1.545967      0.619875      0.808472
  8      1.536016      0.682043     -1.352757
  1      2.462524      0.452421     -1.231485
  1      1.076683     -0.157665     -1.168226
  8      3.348360     -1.481420     -0.054920
  1      2.999251     -0.892213      0.631647
  1      2.540686     -1.822237     -0.455197
  8      0.493887     -1.850827     -0.657360
  1     -0.221761     -2.065066     -1.261682
  1      0.052308     -1.728430      0.188966
 ---------------------------------------------------

Cartesian coordinates of :  IMZW5_13

 ---------------------------------------------------
 Atomic number (AN) and Cartesian coordinates
  AN          X             Y             Z
 ---------------------------------------------------
  6     -0.903727     -2.432419      0.159726
  6     -0.649616     -1.574520      1.192471
  6     -0.124035     -0.655673     -0.722368
  1     -1.303810     -3.430463      0.214456
  1     -0.769622     -1.663327      2.256948
  1      0.136099      0.425545      1.057805
  1      0.224023      0.091001     -1.416196
  7     -0.570701     -1.855071     -1.042178
  7     -0.146167     -0.444303      0.609529
  8     -1.652386      2.454818     -0.534957
  1     -2.206924      3.180223     -0.242528
  1     -2.188249      1.660145     -0.359081
  8      2.736207      1.453569     -0.602354
```

```
  1      3.532709      1.895362     -0.302982
  1      2.885811      0.517106     -0.376860
  8      3.201344     -1.257128      0.043525
  1      2.450821     -1.789184     -0.231391
  1      3.906799     -1.539274     -0.542561
  8     -3.281330      0.164916     -0.147003
  1     -2.831073     -0.464169      0.426209
  1     -3.208321     -0.235864     -1.016550
  8      0.646498      2.298000      1.089721
  1      1.374411      2.075049      0.481706
  1     -0.123000      2.435740      0.509131
 ---------------------------------------------------

Cartesian coordinates of :  IMZW5_14

 ---------------------------------------------------
 Atomic number (AN) and Cartesian coordinates
  AN          X             Y             Z
 ---------------------------------------------------
  6     -2.057254     -1.666980      0.644344
  6     -1.701019     -0.477748      1.212150
  6     -2.148185     -0.210957     -0.910694
  1     -2.128749     -2.631122      1.118132
  1     -1.416914     -0.208049      2.213199
  1     -1.509787      1.430978      0.254959
  1     -2.269847      0.289919     -1.856278
  7     -2.342200     -1.497675     -0.690669
  7     -1.776666      0.446293      0.205961
  8      0.894284     -1.218790     -0.946340
  1      0.114301     -1.718202     -1.206154
  1      0.738228     -1.011192     -0.016820
  8      1.472152      1.639070     -0.774005
  1      1.096728      0.869648     -1.220317
  1      1.684800      1.268925      0.100121
  8      3.646015     -1.231279     -0.254120
  1      2.806472     -1.391791     -0.709594
  1      4.011928     -0.472749     -0.713985
  8      1.871271      0.045157      1.519890
  1      2.176561      0.314968      2.387522
  1      2.634971     -0.393745      1.105478
  8     -0.709884      3.128183      0.102996
  1      0.075793      2.692802     -0.282036
  1     -1.134381      3.554669     -0.643442
 ---------------------------------------------------

Cartesian coordinates of :  IMZW5_15

 ---------------------------------------------------
 Atomic number (AN) and Cartesian coordinates
  AN          X             Y             Z
 ---------------------------------------------------
  6     -0.921636      0.145144      0.641618
  6      0.290605     -0.423189      0.909915
  6     -0.215283     -0.677492     -1.203150
```

1	-1.586200	0.656579	1.317479
1	0.869117	-0.504406	1.812448
1	1.636024	-1.383638	-0.428789
1	-0.116910	-0.976686	-2.232457
7	-1.234768	-0.018652	-0.687198
7	0.721624	-0.951108	-0.276225
8	1.641825	2.322875	-0.160693
1	0.913325	1.843910	0.250828
1	1.683903	3.142800	0.336041
8	-3.885661	0.851923	-0.961635
1	-2.954824	0.543579	-1.024169
1	-4.331551	0.407336	-1.684427
8	3.799709	0.725248	0.701160
1	3.073604	1.296538	0.388200
1	4.589267	1.125535	0.332818
8	-4.347054	-0.759085	1.322479
1	-4.218049	-0.203606	0.532877
1	-3.466445	-1.088328	1.510237
8	3.453579	-1.766605	-0.477850
1	3.627640	-2.420931	0.200888
1	3.651807	-0.911990	-0.046014

Cartesian coordinates of : IMZW5_16

Atomic number (AN) and Cartesian coordinates

AN	X	Y	Z
6	-3.465937	-0.000935	0.151169
6	-2.704917	-0.830333	0.921567
6	-1.707021	-0.422859	-0.981599
1	-4.425736	0.426148	0.386265
1	-2.849577	-1.245397	1.902196
1	-0.756937	-1.611486	0.462575
1	-0.949557	-0.458660	-1.746478
7	-2.839048	0.254258	-1.047649
7	-1.588523	-1.098016	0.178190
8	3.364855	-0.429693	-1.510725
1	2.548119	0.052645	-1.317725
1	3.823090	0.129784	-2.140663
8	1.157528	-1.883095	0.659514
1	1.472842	-2.353777	-0.114415
1	1.257613	-0.943602	0.418934
8	-0.973280	2.393939	0.407643
1	-1.627959	1.932722	-0.130516
1	-0.835982	3.221763	-0.057799
8	1.319640	0.840607	-0.036801
1	2.026924	1.113067	0.562488
1	0.553581	1.414718	0.151357
8	3.939799	0.549055	1.129821
1	3.909181	0.137001	0.250420
1	3.646299	-0.150365	1.717142

Cartesian coordinates of : IMZW5_17

Atomic number (AN) and Cartesian coordinates

AN	X	Y	Z
6	3.181797	-1.134390	-0.343040
6	1.885561	-1.364577	-0.708016
6	1.973628	-0.376271	1.240170
1	4.075614	-1.382623	-0.889512
1	1.445851	-1.823132	-1.575077
1	0.108099	-0.816926	0.355807
1	1.624581	0.078620	2.151760
7	3.235555	-0.517834	0.884080
7	1.125416	-0.885682	0.324048
8	1.893421	1.958725	-1.229129
1	2.059703	1.023813	-1.393707
1	2.603534	2.208556	-0.633124
8	-0.334725	2.234014	0.479858
1	0.418162	2.118730	-0.129384
1	-0.848277	2.942282	0.087644
8	-3.522095	-0.648205	-1.656156
1	-4.076268	-0.609110	-0.859956
1	-3.495541	-1.581525	-1.874898
8	-1.675973	-0.188260	0.485875
1	-2.084953	-0.331622	-0.382978
1	-1.307845	0.716769	0.443135
8	-4.474520	-0.335334	1.032460
1	-3.519398	-0.172447	1.095758
1	-4.864846	0.537138	1.109681

Cartesian coordinates of : IMZW6_ 1

Atomic number (AN) and Cartesian coordinates

AN	X	Y	Z
6	-2.587421	-1.098855	0.272085
6	-1.684796	-1.852389	-0.423193
6	-0.673359	-0.688961	1.126720
1	-3.653152	-1.042497	0.130704
1	-1.794209	-2.540738	-1.241280
1	0.450692	-1.894360	-0.174400
1	0.127637	-0.302576	1.733637
7	-1.949544	-0.372528	1.250622
7	-0.468873	-1.586394	0.144724
8	-1.199576	2.385820	0.849912
1	-1.732138	3.144577	1.094812
1	-1.661168	1.611568	1.212186
8	-1.349221	1.220163	-1.745513
1	-1.813100	0.406175	-1.518970
1	-1.306579	1.699376	-0.899627

8	2.790925	-0.291454	1.671248
1	2.361987	0.501799	1.293585
1	3.721755	-0.063468	1.700982
8	2.283093	-1.761692	-0.688859
1	2.540992	-1.308736	0.134788
1	2.033818	-1.028914	-1.273728
8	1.584449	1.863192	0.379589
1	0.662177	2.054852	0.607182
1	1.528186	1.435144	-0.491926
8	1.286171	0.485447	-2.093148
1	1.634579	0.955819	-2.852228
1	0.334176	0.713863	-2.062647

Cartesian coordinates of : IMZW6_ 2

Atomic number (AN) and Cartesian coordinates

AN	X	Y	Z
6	-0.768840	2.551916	0.304237
6	0.596146	2.536498	0.278048
6	-0.202463	0.902245	-0.935693
1	-1.418180	3.237098	0.821464
1	1.334792	3.164572	0.741548
1	1.883526	1.136787	-0.708593
1	-0.217131	0.039719	-1.580814
7	-1.265626	1.528625	-0.466158
7	0.939685	1.487171	-0.529439
8	-3.480756	-0.165117	-0.662184
1	-3.530303	-0.431159	-1.582060
1	-2.786082	0.532670	-0.646809
8	0.018615	-0.241939	2.228047
1	-0.075609	0.683806	1.983376
1	-0.656543	-0.702693	1.696732
8	0.253132	-2.745035	-1.128186
1	-0.495581	-2.503657	-0.551060
1	0.304929	-3.700049	-1.061478
8	3.446322	0.128272	-0.850522
1	3.036721	-0.520672	-0.241523
1	4.115143	0.563331	-0.318952
8	-1.687174	-1.811157	0.637358
1	-2.146093	-2.473409	1.157392
1	-2.393581	-1.310669	0.177977
8	2.036561	-1.620328	0.756432
1	1.410244	-1.077853	1.266805
1	1.472675	-2.049923	0.088055

Cartesian coordinates of : IMZW6_ 3

Atomic number (AN) and Cartesian coordinates

AN X Y Z

6	0.768821	2.551918	0.304231
6	-0.596164	2.536496	0.278043
6	0.202449	0.902241	-0.935691
1	1.418161	3.237106	0.821452
1	-1.334813	3.164565	0.741547
1	-1.883542	1.136773	-0.708581
1	0.217120	0.039711	-1.580806
7	1.265610	1.528628	-0.466162
7	-0.939701	1.487162	-0.529437
8	-3.446321	0.128245	-0.850522
1	-3.036718	-0.520707	-0.241532
1	-4.115116	0.563324	-0.318933
8	-0.018643	-0.241933	2.228039
1	0.075598	0.683814	1.983384
1	0.656532	-0.702687	1.696744
8	1.687192	-1.811126	0.637373
1	2.146110	-2.473410	1.157366
1	2.393600	-1.310633	0.177995
8	-0.253088	-2.745036	-1.128181
1	0.495613	-2.503634	-0.551049
1	-0.304840	-3.700053	-1.061491
8	-2.036553	-1.620357	0.756415
1	-1.472662	-2.049942	0.088035
1	-1.410239	-1.077871	1.266780
8	3.480761	-0.165091	-0.662170
1	2.786078	0.532687	-0.646812
1	3.530336	-0.431137	-1.582043

Cartesian coordinates of : IMZW6_ 4

Atomic number (AN) and Cartesian coordinates

AN	X	Y	Z
6	2.226706	-1.587608	-0.276046
6	1.202828	-2.117620	0.456351
6	0.420912	-0.802785	-1.103712
1	3.283780	-1.753663	-0.157046
1	1.185540	-2.795207	1.290493
1	-0.905240	-1.748222	0.227957
1	-0.295962	-0.269538	-1.705026
7	1.731619	-0.764924	-1.261173
7	0.056589	-1.613179	-0.093051
8	-1.120551	2.192457	-0.320443
1	-0.204011	2.173055	-0.634648
1	-1.080282	1.750246	0.547024
8	-2.940572	0.384156	-1.375718
1	-2.242095	1.011909	-1.101301
1	-2.591435	-0.027931	-2.168219
8	1.801544	1.086335	1.650244
1	2.053680	0.180434	1.440628
1	1.792341	1.524313	0.781688

8	-2.745060	-1.627047	0.548194
1	-2.772422	-1.180584	1.396768
1	-2.858592	-0.904075	-0.100880
8	1.665304	2.089305	-1.011615
1	2.300803	2.692468	-1.400844
1	1.907303	1.203914	-1.333489
8	-0.921485	0.813494	2.139445
1	-1.156786	1.432466	2.833025
1	0.049808	0.895619	2.063021

Cartesian coordinates of : IMZW6_ 5

Atomic number (AN) and Cartesian coordinates

AN	X	Y	Z
6	1.381062	-2.681963	-0.246359
6	2.450047	-1.871996	0.002464
6	0.595169	-0.714571	0.055667
1	1.378305	-3.739358	-0.447285
1	3.505350	-2.066035	0.058621
1	2.434174	0.245947	0.388457
1	-0.059517	0.135139	0.160157
7	0.218448	-1.951537	-0.211849
7	1.930480	-0.621653	0.194596
8	-2.532911	-1.719313	-0.312109
1	-3.054765	-2.521138	-0.366005
1	-1.590148	-2.007184	-0.320682
8	-2.115255	0.700104	-1.764285
1	-1.917797	1.265063	-1.003051
1	-2.342861	-0.149631	-1.358719
8	-1.491970	2.257079	0.646733
1	-2.234636	2.862023	0.680889
1	-1.781728	1.463926	1.143069
8	2.856662	2.055471	0.648541
1	3.668379	2.241373	0.173500
1	2.148762	2.319044	0.028526
8	-2.253836	-0.071107	1.929663
1	-2.419583	-0.685991	1.190724
1	-1.431791	-0.388922	2.309713
8	0.718566	2.576712	-1.061301
1	0.694622	1.791496	-1.613230
1	-0.046996	2.466206	-0.472484

Cartesian coordinates of : IMZW6_ 6

Atomic number (AN) and Cartesian coordinates

AN	X	Y	Z
6	-2.324175	1.395305	-0.245180
6	-1.290659	2.227288	0.079097

```
  6     -0.584388      0.598127     -1.196147
  1     -3.357153      1.473175      0.047899
  1     -1.241636      3.121277      0.673319
  1      0.776508      2.033056     -0.510763
  1      0.098268     -0.004259     -1.771307
  7     -1.878397      0.375240     -1.053866
  7     -0.190621      1.706798     -0.543640
  8      2.547346     -0.775921     -0.138901
  1      1.925234     -1.367528     -0.599383
  1      2.138139     -0.614664      0.731751
  8      2.640847      1.936581     -0.761169
  1      2.651166      0.971943     -0.599553
  1      3.067143      2.306087      0.014101
  8      0.841625     -2.586512     -1.470119
  1     -0.012417     -2.565474     -1.001843
  1      1.212881     -3.441458     -1.245600
  8     -1.241793     -0.619425      2.144317
  1     -1.578732      0.188337      1.741246
  1     -1.372818     -1.288043      1.450559
  8     -1.601039     -2.281969     -0.127074
  1     -1.921682     -1.478359     -0.581070
  1     -2.306996     -2.925124     -0.215524
  8      1.470869     -0.087162      2.343101
  1      0.519115     -0.306631      2.294486
  1      1.818608     -0.685651      3.006356
 ---------------------------------------------------

Cartesian coordinates of :  IMZW6_ 7

 ---------------------------------------------------
 Atomic number (AN) and Cartesian coordinates
  AN          X             Y             Z
 ---------------------------------------------------
  6     -2.253313     -1.755144      0.452610
  6     -2.664570     -0.505660      0.815311
  6     -1.408262     -0.403802     -0.971603
  1     -2.475810     -2.693374      0.931297
  1     -3.281150     -0.151953      1.621302
  1     -2.158358      1.363466     -0.111734
  1     -0.866103      0.024964     -1.797947
  7     -1.472114     -1.689700     -0.676978
  7     -2.126631      0.343306     -0.113085
  8      1.273133     -2.244598     -0.494384
  1      1.582988     -3.140911     -0.355558
  1      0.315290     -2.314641     -0.675657
  8      0.795938     -0.338110      1.535682
  1     -0.083442     -0.521069      1.878153
  1      0.967340     -1.059564      0.903631
  8     -1.628650      3.182529     -0.132963
  1     -1.762216      3.505842     -1.025702
  1     -0.702378      2.877408     -0.125098
  8      0.880291      1.960788     -0.061193
  1      1.594320      2.479284      0.316801
  1      0.787774      1.201423      0.548013
```

8	2.449929	0.091594	-1.589971
1	2.043072	-0.742914	-1.307986
1	1.857344	0.769493	-1.228798
8	3.626861	0.276320	1.058880
1	3.358425	0.214987	0.128266
1	2.790968	0.171777	1.525153

Cartesian coordinates of : IMZW6_ 8

Atomic number (AN) and Cartesian coordinates

AN	X	Y	Z
6	2.604756	-1.665816	0.473339
6	3.003821	-0.388491	0.737223
6	1.424846	-0.393218	-0.774991
1	2.968985	-2.580070	0.909359
1	3.740094	0.015331	1.407597
1	2.224648	1.430093	-0.107404
1	0.712353	-0.014546	-1.488917
7	1.616905	-1.666441	-0.482463
7	2.244445	0.410341	-0.073125
8	-1.097563	-0.295396	1.512920
1	-1.038744	-1.031044	0.881662
1	-2.052978	-0.112300	1.530629
8	-1.099125	-2.372912	-0.528002
1	-0.120742	-2.367991	-0.551875
1	-1.347475	-3.237742	-0.198298
8	1.584368	3.199489	-0.325396
1	0.673063	2.867469	-0.220282
1	1.763554	3.660351	0.495997
8	-2.480431	-0.082590	-1.523672
1	-2.014455	-0.884609	-1.240559
1	-1.894027	0.635904	-1.240107
8	-3.811132	0.312268	0.945073
1	-3.497604	0.124769	0.041077
1	-4.391050	-0.422690	1.152126
8	-0.906513	1.948745	-0.172058
1	-1.617099	2.502457	0.160079
1	-0.855342	1.215641	0.473683

Cartesian coordinates of : IMZW6_ 9

Atomic number (AN) and Cartesian coordinates

AN	X	Y	Z
6	-1.101545	-2.055733	0.862067
6	0.261196	-2.119265	0.803511
6	-0.520192	-1.029893	-0.924129
1	-1.756779	-2.460761	1.613978
1	0.992941	-2.560219	1.455680

```
  1      1.563900    -1.306494    -0.688056
  1     -0.525625    -0.478591    -1.849918
  7     -1.588205    -1.375733    -0.229558
  7      0.613634    -1.471575    -0.347329
  8      3.228959    -0.791642    -1.369475
  1      3.818120    -0.873747    -0.616737
  1      2.931907     0.136517    -1.331815
  8      2.213566     1.050105     1.656397
  1      2.529289     1.781967     2.189370
  1      1.241464     1.071631     1.769013
  8      2.046232     1.740818    -1.040886
  1      2.117619     1.594406    -0.076956
  1      1.150137     1.461592    -1.242808
  8     -3.644134     0.430508    -0.843916
  1     -4.233446     0.529904    -0.093981
  1     -3.034574    -0.294467    -0.580598
  8     -1.632468     2.324163    -0.449637
  1     -2.365399     1.703783    -0.634870
  1     -1.067301     2.256016    -1.221347
  8     -0.548515     1.109879     1.816636
  1     -0.888571     1.506172     0.989880
  1     -0.797567     0.182141     1.755720
 ---------------------------------------------------

Cartesian coordinates of :  IMZW6_10

 ---------------------------------------------------
 Atomic number (AN) and Cartesian coordinates
  AN          X             Y             Z
 ---------------------------------------------------
  6     -1.904294    -1.760166    -0.340675
  6     -0.582731    -1.990635    -0.595215
  6     -0.808656    -0.777325     1.210500
  1     -2.758820    -2.094619    -0.903669
  1     -0.079485    -2.539448    -1.370625
  1      1.116540    -1.319292     0.519299
  1     -0.533854    -0.206040     2.080827
  7     -2.042883    -1.004375     0.800050
  7      0.100973    -1.365771     0.411456
  8      2.084408     1.306176     1.930106
  1      2.807847     1.923480     2.051710
  1      1.671178     1.586518     1.093526
  8     -1.457903     1.315631    -1.559915
  1     -2.104908     1.468201    -0.845125
  1     -1.427316     0.356144    -1.635639
  8      1.051987     1.878807    -0.603790
  1      0.128213     1.715964    -0.892650
  1      1.258057     2.758831    -0.924462
  8     -3.234193     1.561652     0.612259
  1     -3.017868     0.626003     0.795004
  1     -4.137177     1.539308     0.290299
  8      2.440061    -0.128469    -1.976992
  1      1.758598    -0.768878    -2.192597
  1      1.946327     0.605560    -1.564795
```

```
  8      2.987047     -1.046813      0.659413
  1      2.807296     -0.226096      1.152226
  1      2.961575     -0.761738     -0.270187
 ---------------------------------------------------

Cartesian coordinates of :  IMZW6_11

 ---------------------------------------------------
 Atomic number (AN) and Cartesian coordinates
  AN         X             Y             Z
 ---------------------------------------------------
  6     -0.121793     -2.377729      0.575019
  6      1.216126     -2.175112      0.398239
  6      0.071572     -0.804403     -0.864704
  1     -0.609682     -3.086775      1.221521
  1      2.081352     -2.642988      0.831139
  1      2.174381     -0.712805     -0.847651
  1     -0.131958     -0.025484     -1.580500
  7     -0.837563     -1.521294     -0.227222
  7      1.319914     -1.178823     -0.534873
  8      1.570809      2.248657     -0.145563
  1      1.946690      3.066277      0.185236
  1      1.350764      1.741307      0.661221
  8      3.447429      0.579069     -1.317528
  1      4.194306      0.486454     -0.723652
  1      2.882722      1.254023     -0.890905
  8     -2.129989      2.073205     -0.659973
  1     -2.587708      1.267925     -0.970635
  1     -2.831091      2.720549     -0.564850
  8     -3.267176     -0.413258     -1.230100
  1     -2.512317     -0.909438     -0.852684
  1     -3.236642     -0.618238     -2.166404
  8      0.909269      0.777288      2.132978
  1      1.208679     -0.122997      1.978645
  1     -0.061922      0.718769      2.032480
  8     -1.846492      0.599640      1.733395
  1     -1.767415     -0.241697      1.266532
  1     -1.942841      1.232588      0.998164
 ---------------------------------------------------

Cartesian coordinates of :  IMZW6_12

 ---------------------------------------------------
 Atomic number (AN) and Cartesian coordinates
  AN         X             Y             Z
 ---------------------------------------------------
  6      3.354814     -0.553258     -0.252328
  6      2.754974     -1.776916     -0.194060
  6      1.357203     -0.212964      0.431808
  1      4.361439     -0.319903     -0.553546
  1      3.105566     -2.766587     -0.421817
  1      0.715656     -2.191908      0.365343
  1      0.445336      0.237845      0.786537
  7      2.474791      0.425913      0.143405
```

7	1.483085	-1.539955	0.250014
8	-3.859285	-0.220500	-0.399256
1	-4.120884	-1.128898	-0.235012
1	-3.078015	-0.304648	-0.975705
8	-2.039578	0.329752	1.640461
1	-2.797435	0.171451	1.044451
1	-2.431582	0.618210	2.466840
8	1.517849	3.069455	0.006856
1	1.561337	3.440734	0.889704
1	1.996265	2.215925	0.079055
8	-1.327395	-2.242357	0.453479
1	-1.492027	-1.552734	1.111113
1	-1.355336	-1.741001	-0.374254
8	-1.362790	-0.310133	-1.699651
1	-1.359432	-0.214644	-2.653396
1	-1.153065	0.578969	-1.347220
8	-1.098389	2.113895	-0.385023
1	-0.191377	2.432650	-0.216669
1	-1.356816	1.660773	0.433196

Cartesian coordinates of : IMZW6_13

Atomic number (AN) and Cartesian coordinates

AN	X	Y	Z
6	-3.041038	-0.622344	-0.081023
6	-2.471946	-1.706263	0.521036
6	-0.926078	-0.322405	-0.173186
1	-4.087311	-0.415122	-0.225495
1	-2.891951	-2.581986	0.980327
1	-0.363039	-2.081529	0.790375
1	0.057355	0.076735	-0.361242
7	-2.066939	0.244079	-0.516838
7	-1.122021	-1.496982	0.452617
8	1.739874	-1.724237	-1.855412
1	2.533240	-2.111488	-2.229472
1	1.995785	-0.803891	-1.652456
8	1.828609	0.490959	1.712899
1	1.191973	1.232642	1.645830
1	2.531798	0.814362	2.278576
8	2.444446	0.849396	-1.018799
1	2.305927	0.719401	-0.062251
1	1.758015	1.474354	-1.263956
8	0.071370	2.586194	1.343330
1	0.556791	3.396288	1.509394
1	-0.210117	2.659162	0.410770
8	-0.966121	2.728371	-1.265133
1	-1.493667	1.915368	-1.116847
1	-0.311209	2.464612	-1.914785
8	1.626098	-2.253159	0.936986
1	1.716717	-2.160470	-0.027721
1	1.712570	-1.342250	1.256566

Cartesian coordinates of : IMZW6_14

Atomic number (AN) and Cartesian coordinates

AN	X	Y	Z
6	2.979589	1.053199	-0.110549
6	3.128563	-0.285579	0.104573
6	1.010325	0.218124	-0.103084
1	3.746065	1.805711	-0.179116
1	3.996172	-0.901649	0.253019
1	1.569255	-1.769178	0.231201
1	-0.058359	0.083121	-0.152328
7	1.648033	1.365568	-0.240917
7	1.862001	-0.801800	0.106281
8	-1.564489	1.528184	1.810885
1	-1.291050	2.026353	1.020028
1	-2.486712	1.765795	1.926678
8	-0.689603	2.797009	-0.570265
1	0.253485	2.531447	-0.507493
1	-0.695421	3.752299	-0.641747
8	0.290746	-3.215436	0.334427
1	-0.245319	-2.819419	-0.372564
1	-0.139124	-2.881897	1.128562
8	-1.324834	-1.840108	-1.576028
1	-2.087496	-2.364244	-1.827829
1	-1.663486	-0.924472	-1.536714
8	-2.438306	0.694961	-1.224908
1	-1.821831	1.439935	-1.127469
1	-2.774842	0.571215	-0.332867
8	-1.791269	-1.270010	1.439175
1	-1.768658	-1.426143	0.487550
1	-1.671735	-0.306506	1.521614

Cartesian coordinates of : IMZW6_15

Atomic number (AN) and Cartesian coordinates

AN	X	Y	Z
6	-1.181053	2.542518	-0.038200
6	0.154917	2.779927	0.101058
6	-0.171467	0.820228	-0.812739
1	-2.004666	3.177848	0.238724
1	0.702401	3.613475	0.501195
1	1.788866	1.488218	-0.430077
1	0.053709	-0.135293	-1.256805
7	-1.382909	1.312245	-0.618975
7	0.783179	1.675732	-0.405265
8	3.497505	0.768944	-0.351587
1	3.139747	-0.039345	0.066800

1	3.725773	0.493439	-1.241093
8	-3.132536	-0.867140	-0.971251
1	-2.650380	-0.021633	-0.840594
1	-3.901055	-0.793762	-0.402613
8	0.946106	-2.798440	-1.094618
1	0.124775	-2.692246	-0.570793
1	1.169053	-3.726111	-1.000781
8	-1.026515	-0.074546	2.256590
1	-0.179077	0.367326	2.352205
1	-1.452895	0.420108	1.548038
8	-1.250688	-2.342367	0.504252
1	-1.931205	-1.891343	-0.030625
1	-0.998791	-1.663457	1.149732
8	2.268252	-1.375979	0.914246
1	1.513562	-0.953738	1.331487
1	1.866919	-1.899147	0.193098

Cartesian coordinates of : IMZW6_16

Atomic number (AN) and Cartesian coordinates

AN	X	Y	Z
6	0.961008	-2.032627	0.882418
6	-0.103948	-2.588289	0.234026
6	-0.658972	-0.654966	1.097595
1	1.955356	-2.431435	0.988225
1	-0.225307	-3.511266	-0.302570
1	-2.048766	-1.708742	-0.041131
1	-1.270325	0.194123	1.351599
7	0.607404	-0.821858	1.430988
7	-1.132228	-1.697528	0.391929
8	-1.322086	1.431924	-1.333461
1	-1.215528	1.863653	-0.468816
1	-0.518809	0.893357	-1.445980
8	0.919302	-0.199896	-1.936042
1	0.686714	-1.071052	-1.600265
1	1.805706	-0.046484	-1.560165
8	-1.076749	2.758964	1.209725
1	-1.116285	3.692096	0.991363
1	-0.126753	2.559864	1.222874
8	3.473518	0.170454	-0.747099
1	3.137786	0.851718	-0.147527
1	4.048795	0.648122	-1.347886
8	-3.410577	-0.396292	-0.864503
1	-3.847415	-0.079860	-0.071758
1	-2.702002	0.258974	-1.018202
8	1.585128	1.793090	0.735487
1	1.241736	1.808383	-0.163609
1	1.332057	0.903591	1.056347

Cartesian coordinates of : IMZW6_17

```
 ---------------------------------------------------
 Atomic number (AN) and Cartesian coordinates
  AN          X             Y             Z
 ---------------------------------------------------
  6      -3.431787     -0.321621      0.037977
  6      -3.362940      1.034282      0.158186
  6      -1.368388      0.197553     -0.197791
  1      -4.302701     -0.950800      0.098716
  1      -4.109675      1.786095      0.334151
  1      -1.632482      2.266747      0.034287
  1      -0.303640      0.160512     -0.355150
  7      -2.179431     -0.841548     -0.185963
  7      -2.039115      1.347531      0.004304
  8       1.253444      2.038643     -1.393653
  1       1.350414      1.104428     -1.636677
  1       1.242784      1.998115     -0.427910
  8      -0.031647     -2.580511     -0.484879
  1      -0.935908     -2.208840     -0.421539
  1      -0.138246     -3.532645     -0.499705
  8       0.953215     -1.319777      1.829545
  1       1.874352     -1.571608      1.922430
  1       0.653982     -1.800654      1.035858
  8       1.810411     -0.779471     -1.705037
  1       1.191983     -1.413231     -1.310076
  1       2.444817     -0.577372     -0.995899
  8       3.631037      0.220560      0.240733
  1       4.069472      0.844996     -0.340991
  1       2.933233      0.747574      0.667397
  8       1.329766      1.404403      1.423825
  1       1.509070      1.797693      2.279500
  1       1.091252      0.475056      1.612712
 ---------------------------------------------------

Cartesian coordinates of :  IMZW6_18

 ---------------------------------------------------
 Atomic number (AN) and Cartesian coordinates
  AN          X             Y             Z
 ---------------------------------------------------
  6       2.970922      0.412474     -0.840611
  6       2.388190     -0.718490     -1.333390
  6       1.971131     -0.516647      0.803277
  1       3.560798      1.141347     -1.369667
  1       2.366471     -1.152165     -2.316395
  1       1.156462     -2.126582     -0.273372
  1       1.566833     -0.744667      1.775270
  7       2.715962      0.532165      0.505680
  7       1.764348     -1.306137     -0.266734
  8      -0.327712     -3.249908      0.034195
  1      -0.811662     -2.478824      0.396035
  1      -0.121995     -3.782355      0.804435
  8      -0.503304      1.282040     -0.473678
  1       0.088007      1.012135     -1.180628
```

```
  1      0.034046      1.882279      0.080321
  8     -4.180183     -0.555326      0.161774
  1     -3.909235      0.284800     -0.258729
  1     -4.669398     -0.280850      0.939256
  8      1.186234      2.861022      1.097183
  1      1.840272      2.136317      1.053447
  1      0.861890      2.831347      1.999362
  8     -3.193674      1.768861     -0.994133
  1     -3.323205      1.728003     -1.943118
  1     -2.234530      1.643312     -0.876715
  8     -1.507470     -0.939659      0.984681
  1     -1.090881     -0.206877      0.499498
  1     -2.448627     -0.869678      0.742545
 ---------------------------------------------------

Cartesian coordinates of :  IMZW7_ 1

 ---------------------------------------------------
 Atomic number (AN) and Cartesian coordinates
  AN          X             Y             Z
 ---------------------------------------------------
  6     -1.776051      2.189773      0.123064
  6     -0.456420      2.522550      0.010116
  6     -0.668190      0.605207      1.040411
  1     -2.634307      2.742289     -0.218606
  1      0.040896      3.369957     -0.425475
  1      1.251389      1.407374      0.653250
  1     -0.391371     -0.302820      1.549426
  7     -1.903952      0.988373      0.777478
  7      0.234691      1.507506      0.612427
  8      3.083253      0.984241      0.511409
  1      2.946596      0.238023      1.121555
  1      2.887514      0.613567     -0.362267
  8     -1.393798     -2.097460     -0.839874
  1     -2.125180     -1.931369     -0.210413
  1     -1.692166     -2.825748     -1.387560
  8     -3.405658     -1.373812      0.921467
  1     -3.208143     -1.743348      1.783960
  1     -3.010701     -0.474333      0.947453
  8      1.358027     -2.081942     -0.224918
  1      1.659857     -1.376184     -0.821262
  1      0.396265     -2.108381     -0.361965
  8      2.061817      0.070979     -1.975374
  1      2.452867     -0.302553     -2.766684
  1      1.105499      0.149912     -2.173584
  8     -0.669485      0.141076     -2.370485
  1     -0.971681     -0.600459     -1.812859
  1     -0.999163      0.927505     -1.923581
  8      2.274928     -1.131497      2.201329
  1      1.907412     -1.547596      1.395518
  1      3.000544     -1.704854      2.453775
 ---------------------------------------------------

Cartesian coordinates of :  IMZW7_ 2
```

Atomic number (AN) and Cartesian coordinates

AN	X	Y	Z
6	-3.055036	-0.820003	0.028047
6	-3.155569	0.502011	0.344210
6	-1.049326	-0.083245	0.173669
1	-3.849201	-1.529208	-0.127832
1	-3.998832	1.146398	0.510355
1	-1.563022	1.891952	0.638079
1	0.025679	0.005558	0.172700
7	-1.733252	-1.181798	-0.077813
7	-1.866903	0.953852	0.435415
8	1.574661	-1.490372	1.735308
1	1.206006	-2.067111	1.039278
1	2.258617	-2.012682	2.157840
8	2.746384	0.439607	0.003833
1	2.134454	1.190967	0.067643
1	2.401512	-0.198888	0.652420
8	0.814862	1.165421	2.623484
1	1.008893	0.256479	2.356784
1	0.759396	1.640069	1.783211
8	0.755801	2.538577	0.055196
1	1.192952	3.384521	-0.056711
1	0.495072	2.262852	-0.847849
8	0.003805	1.498976	-2.407908
1	0.467713	0.639570	-2.369974
1	0.436349	1.967744	-3.123982
8	0.441978	-2.842907	-0.427791
1	-0.460131	-2.461010	-0.339986
1	0.318865	-3.791827	-0.482345
8	1.513416	-0.847343	-2.162997
1	2.020777	-0.418140	-1.447880
1	1.098321	-1.599880	-1.713525

Cartesian coordinates of : IMZW7_ 3

Atomic number (AN) and Cartesian coordinates

AN	X	Y	Z
6	-2.238699	1.660667	-0.777728
6	-1.343575	2.512090	-0.197139
6	-0.316729	0.740131	-0.963974
1	-3.304106	1.779824	-0.876601
1	-1.460345	3.467455	0.280677
1	0.786304	2.260523	-0.014032
1	0.492748	0.068717	-1.197974
7	-1.588388	0.552650	-1.268315
7	-0.122324	1.911948	-0.330954
8	2.065120	-1.734653	-1.889272
1	2.746435	-2.397509	-2.014409

1	1.430570	-2.153404	-1.272500
8	0.340420	-2.713230	0.039852
1	-0.591454	-2.553680	-0.185432
1	0.510150	-2.087743	0.763800
8	-2.335501	-1.988771	-0.419608
1	-2.192303	-1.154209	-0.916411
1	-3.075153	-2.425266	-0.844937
8	0.696069	-0.756411	2.117997
1	-0.268039	-0.587845	2.090495
1	0.841035	-1.242323	2.931525
8	-2.041653	-0.395452	1.917732
1	-2.176924	0.465493	1.509167
1	-2.259350	-1.020337	1.205944
8	2.564308	2.508709	0.445130
1	2.762917	1.554878	0.351142
1	2.985623	2.915923	-0.313599
8	2.880617	-0.233588	0.389333
1	2.663893	-0.690830	-0.441906
1	2.121960	-0.422009	0.963675

Cartesian coordinates of : IMZW7_ 4

Atomic number (AN) and Cartesian coordinates

AN	X	Y	Z
6	1.438446	-2.287851	0.325829
6	2.473901	-1.411531	0.471684
6	0.999819	-0.613186	-0.934421
1	1.300696	-3.243272	0.802233
1	3.370340	-1.443807	1.063280
1	2.673537	0.530994	-0.443192
1	0.537558	0.065246	-1.631864
7	0.518386	-1.787073	-0.565663
7	2.182584	-0.356483	-0.350327
8	-2.024007	-1.656102	-1.840878
1	-1.178754	-1.888976	-1.407177
1	-2.688438	-1.895355	-1.190684
8	-1.651571	-1.262772	1.538080
1	-1.161013	-1.462529	0.730756
1	-2.210418	-0.498477	1.299112
8	0.047056	2.379320	0.189931
1	0.146484	1.732692	0.913728
1	-0.540266	1.938524	-0.442190
8	0.335064	0.575169	2.341837
1	1.179119	0.127801	2.240273
1	-0.320921	-0.115308	2.129414
8	2.669516	2.446468	-0.752524
1	1.726826	2.477566	-0.492767
1	2.653175	2.525947	-1.707844
8	-1.871632	1.097100	-1.518768
1	-1.935096	0.135149	-1.692870
1	-2.362510	1.516862	-2.227212

8	-3.051218	1.106523	1.001215
1	-2.494490	1.688374	1.523706
1	-2.701276	1.203221	0.095532

Cartesian coordinates of : IMZW7_ 5

Atomic number (AN) and Cartesian coordinates

AN	X	Y	Z
6	1.787164	2.154424	0.193748
6	0.468171	2.491717	0.083424
6	0.673508	0.542445	1.053761
1	2.647619	2.717788	-0.123878
1	-0.026157	3.354017	-0.325555
1	-1.244504	1.358509	0.677841
1	0.395635	-0.384580	1.527295
7	1.910704	0.932828	0.810309
7	-0.227229	1.457289	0.647513
8	-2.303417	-1.144406	2.225879
1	-1.616507	-0.681565	2.709524
1	-1.842503	-1.514746	1.445566
8	-2.087351	-0.028470	-1.951628
1	-1.157337	0.130931	-2.196257
1	-2.497959	-0.385612	-2.740649
8	3.357861	-1.480757	0.913141
1	3.085406	-1.873021	1.744642
1	2.986518	-0.573305	0.944866
8	-1.329328	-2.119686	-0.161792
1	-1.652636	-1.437375	-0.774259
1	-0.372622	-2.155951	-0.346529
8	0.658767	0.267413	-2.360846
1	0.975234	0.372026	-3.261011
1	0.961950	1.063132	-1.907465
8	-3.086191	0.970106	0.509697
1	-2.891843	0.590599	-0.360594
1	-2.955220	0.227586	1.125346
8	1.359919	-2.141471	-0.992473
1	2.040465	-1.952087	-0.322037
1	1.245001	-1.300519	-1.463040

Cartesian coordinates of : IMZW7_ 6

Atomic number (AN) and Cartesian coordinates

AN	X	Y	Z
6	-1.975114	-2.083922	-0.307953
6	-2.760630	-1.022129	-0.656052
6	-1.415391	-0.470909	0.978449
1	-1.968383	-3.073163	-0.732268
1	-3.529043	-0.903112	-1.397799

1	-2.694829	0.968349	0.162455
1	-0.945217	0.138008	1.732448
7	-1.136887	-1.735801	0.724470
7	-2.398276	-0.005901	0.185536
8	1.611150	-1.994369	1.415519
1	1.988779	-1.889465	0.526511
1	0.650894	-2.047195	1.234671
8	2.362354	-1.250504	-1.194449
1	2.806499	-0.391741	-1.042255
1	2.915257	-1.712742	-1.827013
8	0.058750	0.043344	-2.122357
1	-0.701736	-0.508537	-1.911881
1	0.825657	-0.498373	-1.860068
8	0.281317	2.146048	-0.259413
1	0.688330	1.723178	0.512250
1	0.166955	1.409919	-0.890390
8	-2.336564	2.847028	0.398900
1	-1.404980	2.672337	0.158161
1	-2.683239	3.340317	-0.346339
8	1.791558	0.756243	1.752027
1	2.036257	0.988284	2.649283
1	1.720086	-0.221123	1.743832
8	3.269282	1.289408	-0.554301
1	2.899386	1.165765	0.338884
1	2.579500	1.795385	-0.994597

Cartesian coordinates of : IMZW7_ 7

Atomic number (AN) and Cartesian coordinates

AN	X	Y	Z
6	1.231923	-2.513713	-0.073946
6	2.321961	-1.715165	-0.275220
6	0.498828	-0.686969	-0.912043
1	1.202816	-3.511193	0.329566
1	3.370288	-1.863540	-0.091176
1	2.350600	0.294415	-1.041419
1	-0.130388	0.094809	-1.304937
7	0.090427	-1.867254	-0.483999
7	1.836217	-0.559920	-0.822306
8	2.765614	2.129384	-1.184688
1	2.070574	2.285260	-0.512571
1	2.370902	2.427737	-2.005930
8	-2.676566	-1.510920	-0.070066
1	-2.571944	-0.927497	0.697065
1	-1.755461	-1.807416	-0.225600
8	-2.615910	0.312915	-2.174442
1	-3.514735	0.585815	-2.366522
1	-2.714657	-0.358770	-1.469286
8	0.936330	-0.136801	2.310707
1	1.281970	-0.921188	1.869133
1	1.329522	-0.160512	3.185597

8	0.855566	2.316166	0.803839
1	0.951866	1.485742	1.295279
1	-0.026291	2.242401	0.395712
8	-1.829647	0.213539	2.029195
1	-0.885611	0.054228	2.208492
1	-2.214081	0.435185	2.878792
8	-1.805741	2.068687	-0.080560
1	-1.918068	1.447596	0.659917
1	-2.037257	1.538468	-0.862596

Cartesian coordinates of : IMZW7_ 8

Atomic number (AN) and Cartesian coordinates

AN	X	Y	Z
6	-1.327572	2.313243	0.381118
6	0.011648	2.292744	0.109840
6	-1.031557	0.792521	-1.091438
1	-1.856954	2.951161	1.067891
1	0.834529	2.872760	0.486750
1	1.056149	0.994110	-1.248516
1	-1.178904	-0.000616	-1.805094
7	-1.979869	1.371980	-0.380323
7	0.179692	1.321755	-0.839408
8	-3.802074	-0.829268	-0.422149
1	-3.334927	0.028404	-0.389899
1	-3.885898	-1.009559	-1.360502
8	1.971686	-1.361676	1.347631
1	1.180265	-0.967429	1.749718
1	1.654477	-1.684033	0.488089
8	-1.433147	-2.123574	0.492955
1	-1.135882	-1.464374	1.140689
1	-2.247983	-1.736141	0.126505
8	2.494235	-0.045493	-1.938704
1	2.988965	0.203893	-1.139505
1	2.008022	-0.841944	-1.676354
8	0.787301	-2.200662	-1.148061
1	1.002393	-3.129961	-1.244240
1	-0.054751	-2.187203	-0.645991
8	-0.402539	-0.185939	2.309680
1	-0.509173	0.714340	1.980233
1	-0.671699	-0.144903	3.229513
8	3.503254	0.809234	0.583327
1	2.954388	0.075564	0.929166
1	2.933386	1.577762	0.655108

Cartesian coordinates of : IMZW7_ 9

Atomic number (AN) and Cartesian coordinates

AN X Y Z

6	1.392263	2.302408	0.404100
6	0.052119	2.304692	0.137047
6	1.066756	0.793528	-1.074157
1	1.934074	2.928417	1.092145
1	-0.760687	2.893580	0.521466
1	-1.017478	1.028954	-1.225216
1	1.199179	0.001676	-1.792170
7	2.026930	1.354307	-0.363810
7	-0.134974	1.340801	-0.815502
8	-1.980492	-1.312006	1.329598
1	-1.663363	-1.635863	0.470757
1	-1.189044	-0.918909	1.732735
8	-2.460015	0.030298	-1.938932
1	-1.994206	-0.781356	-1.686816
1	-2.977691	0.262658	-1.148075
8	-3.551127	0.820070	0.542779
1	-3.048183	0.064541	0.909764
1	-4.470096	0.578029	0.668485
8	3.810364	-0.876325	-0.423928
1	3.890807	-1.051346	-1.363566
1	3.357728	-0.011055	-0.385394
8	-0.799209	-2.166002	-1.165420
1	-1.026491	-3.092153	-1.263863
1	0.041539	-2.165219	-0.660995
8	0.415610	-0.195007	2.310887
1	0.544333	0.708661	1.999270
1	0.679337	-0.177572	3.233019
8	1.417615	-2.134844	0.482580
1	2.238644	-1.758310	0.118732
1	1.129114	-1.473725	1.132463

Cartesian coordinates of : IMZW7_10

Atomic number (AN) and Cartesian coordinates

AN	X	Y	Z
6	1.057922	-2.424541	-0.537321
6	2.118350	-1.693570	-0.992622
6	1.381462	-0.867923	0.893724
1	0.601847	-3.288965	-0.988535
1	2.737418	-1.785595	-1.866003
1	2.985423	0.059011	-0.084263
1	1.311581	-0.213459	1.746776
7	0.603784	-1.907141	0.652477
7	2.316341	-0.712049	-0.060899
8	-1.144049	1.362133	0.896710
1	-1.328503	0.508547	1.323478
1	-1.996634	1.609822	0.492575
8	-0.642694	0.212535	-1.733383
1	0.003451	-0.501720	-1.705243
1	-0.633386	0.579235	-0.833357

8	3.688501	1.755907	0.325624
1	2.805915	2.162941	0.228710
1	4.186254	2.094579	-0.420451
8	-1.864080	-1.239701	1.740930
1	-1.021155	-1.656117	1.452598
1	-2.064327	-1.618125	2.598371
8	-3.056768	-1.019796	-0.821818
1	-2.779788	-1.188979	0.093015
1	-2.235803	-0.714578	-1.244262
8	1.114419	2.783231	0.024100
1	0.975691	2.762410	-0.924886
1	0.386461	2.242134	0.374553
8	-3.578665	1.718857	-0.451542
1	-4.292258	1.861983	0.172662
1	-3.562777	0.752088	-0.584436

Cartesian coordinates of : IMZW7_11

Atomic number (AN) and Cartesian coordinates

AN	X	Y	Z
6	-1.993144	2.055871	-0.079882
6	-0.669635	2.351076	0.081337
6	-0.914306	0.713449	-1.345189
1	-2.840825	2.522282	0.392343
1	-0.159119	3.081288	0.682570
1	1.018659	1.407711	-0.854807
1	-0.648922	-0.057072	-2.048757
7	-2.144603	1.029241	-0.983146
7	0.004057	1.488093	-0.738679
8	2.807028	0.950767	-1.066073
1	2.544964	0.057051	-1.353179
1	2.929852	0.879388	-0.102468
8	-1.526090	-0.354154	2.143809
1	-1.531176	0.548039	1.805507
1	-1.996226	-0.855753	1.455083
8	-2.753947	-1.599637	-0.094502
1	-2.825995	-0.722902	-0.513848
1	-3.636155	-1.974208	-0.114688
8	2.857087	0.915493	1.742204
1	2.245678	0.159140	1.825601
1	3.686167	0.589194	2.095887
8	1.058500	-1.206089	1.775448
1	1.279260	-1.811914	2.485826
1	0.162658	-0.874782	1.999612
8	1.818508	-1.459213	-2.093766
1	2.493836	-2.139757	-2.102999
1	1.104974	-1.840092	-1.546513
8	-0.095121	-2.600919	-0.404911
1	0.302900	-2.195331	0.384013
1	-1.011914	-2.285981	-0.391657

Cartesian coordinates of : IMZW7_12

Atomic number (AN) and Cartesian coordinates

AN	X	Y	Z
6	-1.318986	1.881139	-1.106380
6	-0.417827	2.631951	-0.405355
6	0.500262	0.762422	-1.070861
1	-2.345223	2.116396	-1.332708
1	-0.490447	3.593785	0.068212
1	1.627431	2.104692	0.057542
1	1.255859	0.008419	-1.212886
7	-0.736699	0.709618	-1.529118
7	0.738499	1.900347	-0.395100
8	1.672151	-2.528921	-1.461125
1	1.867989	-3.454331	-1.302778
1	0.722817	-2.451819	-1.264058
8	-1.090028	-2.054392	-0.748574
1	-0.745609	-1.863059	0.144850
1	-1.063311	-1.180985	-1.180886
8	-3.575065	-0.891048	0.205407
1	-2.836861	-1.410827	-0.152099
1	-3.796833	-0.291509	-0.510782
8	-0.055350	-1.279626	1.756030
1	-0.698368	-0.554051	1.899949
1	-0.213869	-1.897827	2.471695
8	-1.998410	0.665720	1.927531
1	-2.600900	0.179036	1.331509
1	-1.668571	1.382645	1.375756
8	3.384499	1.511619	0.598291
1	3.580024	1.791920	1.493758
1	3.135532	0.570142	0.691507
8	2.604938	-1.155450	0.858885
1	2.406615	-1.607159	0.020067
1	1.728554	-1.101516	1.274895

Cartesian coordinates of : IMZW7_13

Atomic number (AN) and Cartesian coordinates

AN	X	Y	Z
6	-0.807701	2.478826	0.026428
6	-2.081867	2.004364	0.140000
6	-0.856397	0.608051	-1.015688
1	-0.397061	3.391378	0.422983
1	-2.957836	2.395558	0.623959
1	-2.846066	0.132291	-0.609003
1	-0.596119	-0.263852	-1.592225
7	-0.042211	1.602858	-0.708535
7	-2.096045	0.818250	-0.542755

8	2.358133	0.473909	-1.710741
1	1.618072	1.048351	-1.429653
1	2.435776	0.598288	-2.657974
8	1.408137	-2.043753	-0.790688
1	2.001815	-2.146689	-0.043770
1	1.662937	-1.181999	-1.164184
8	-3.404689	-1.705262	-0.815143
1	-4.114504	-1.915914	-0.206304
1	-2.594893	-1.993460	-0.346809
8	-0.774256	-0.269159	2.426913
1	-1.312609	0.476753	2.148057
1	0.131241	-0.000433	2.182537
8	-1.064950	-2.346194	0.546227
1	-1.019764	-1.626297	1.202071
1	-0.259551	-2.214307	0.015608
8	1.708584	0.711629	1.601095
1	2.482649	0.214669	1.283299
1	1.311773	1.058724	0.791309
8	3.971044	-0.500894	0.376065
1	3.593871	-0.125693	-0.435834
1	4.687825	0.095222	0.600697

Cartesian coordinates of : IMZW7_14

Atomic number (AN) and Cartesian coordinates

AN	X	Y	Z
6	-1.504887	2.268721	0.151716
6	-0.153686	2.375915	-0.015469
6	-0.926898	0.692072	-1.173237
1	-2.154570	2.887994	0.746074
1	0.570925	3.062681	0.382827
1	1.143670	1.152571	-1.200762
1	-0.934194	-0.157611	-1.834670
7	-1.987353	1.210897	-0.582900
7	0.197487	1.366677	-0.868566
8	2.162140	0.539351	2.048402
1	2.581189	0.317335	2.881647
1	1.222738	0.302319	2.180304
8	-0.541759	-0.042140	2.334189
1	-0.879400	0.752178	1.906658
1	-0.818968	-0.764792	1.739683
8	3.029338	-1.287753	0.114551
1	2.224491	-1.706054	-0.243329
1	2.707613	-0.709370	0.830961
8	0.777362	-2.356219	-1.151103
1	0.009714	-2.325374	-0.549780
1	0.946815	-3.292292	-1.270780
8	2.816905	0.708222	-1.806089
1	3.399869	1.432089	-1.570334
1	3.006639	0.017722	-1.139105
8	-1.345802	-2.114241	0.631133

1	-1.554288	-2.907628	1.128149
1	-2.198132	-1.824316	0.244178
8	-3.676385	-1.044987	-0.423047
1	-3.237714	-0.171647	-0.505201
1	-3.768903	-1.346935	-1.328614

Cartesian coordinates of : IMZW7_15

Atomic number (AN) and Cartesian coordinates

AN	X	Y	Z
6	3.058532	-0.947056	0.704765
6	3.016368	0.403467	0.897151
6	1.927960	-0.139585	-0.919850
1	3.538160	-1.691484	1.316835
1	3.425709	1.038833	1.661095
1	1.994917	1.868518	-0.292017
1	1.337964	-0.020733	-1.813275
7	2.381928	-1.284946	-0.443792
7	2.302835	0.904198	-0.157412
8	-3.663239	1.557086	0.653845
1	-3.609413	1.745178	1.592589
1	-3.700735	0.581411	0.607066
8	-3.525491	-1.209140	0.345254
1	-2.927462	-1.179839	-0.427128
1	-4.352487	-1.541909	-0.007799
8	-1.644794	-0.958807	-1.720711
1	-1.047398	-1.609654	-1.316188
1	-1.362316	-0.112390	-1.333834
8	-0.259405	-0.581867	1.612136
1	0.668789	-0.483600	1.846771
1	-0.271388	-1.379546	1.057421
8	0.896156	3.388749	-0.500961
1	0.924129	3.638709	-1.426118
1	0.130187	2.785831	-0.437205
8	-0.020281	-2.730414	-0.255567
1	-0.022813	-3.685873	-0.179985
1	0.912057	-2.473023	-0.397141
8	-1.014597	1.400196	-0.238100
1	-1.926515	1.575416	0.064036
1	-0.668669	0.782008	0.433732

Cartesian coordinates of : IMZW7_16

Atomic number (AN) and Cartesian coordinates

AN	X	Y	Z
6	-1.171539	-2.590520	-0.063776
6	-2.060940	-1.883358	-0.821670
6	-1.452629	-0.716852	0.922968

1	-0.789043	-3.581421	-0.239494
1	-2.576283	-2.115309	-1.735764
1	-2.785962	0.114081	-0.475969
1	-1.397854	0.109094	1.612387
7	-0.795667	-1.856077	1.036822
7	-2.233835	-0.691834	-0.172678
8	3.462496	1.849651	-0.732413
1	3.603570	0.895182	-0.603296
1	3.990387	2.261366	-0.045884
8	0.870607	1.381212	0.228263
1	1.736929	1.712406	-0.074687
1	1.100145	0.739914	0.926174
8	1.697916	-0.728916	1.962614
1	2.345915	-0.939455	1.271617
1	0.920040	-1.264895	1.722775
8	0.947035	-0.641527	-1.745988
1	0.754441	0.062499	-1.099457
1	0.306013	-1.330858	-1.539823
8	-3.454521	1.833074	-0.725699
1	-2.718160	2.258318	-0.242341
1	-3.300111	2.066087	-1.642742
8	-1.375558	2.921261	0.767208
1	-0.569367	2.394342	0.608706
1	-1.615953	2.711887	1.671666
8	3.330232	-0.930304	-0.351722
1	2.546504	-0.992989	-0.932179
1	3.940308	-1.596095	-0.673932

Cartesian coordinates of : IMZW7_17

Atomic number (AN) and Cartesian coordinates

AN	X	Y	Z
6	-3.309341	-0.792456	-0.781956
6	-3.252021	0.558823	-0.960240
6	-1.933826	-0.048121	0.675541
1	-3.892142	-1.511889	-1.330963
1	-3.743163	1.218585	-1.651573
1	-2.039281	1.968903	0.129270
1	-1.227096	0.038807	1.484183
7	-2.485183	-1.170098	0.251137
7	-2.373661	1.017642	-0.017613
8	3.681399	1.632083	-0.780236
1	3.752613	0.663211	-0.705813
1	4.226223	1.963822	-0.064287
8	1.065824	1.332867	0.208627
1	1.964367	1.545011	-0.106507
1	1.208037	0.638781	0.877879
8	-0.813053	3.288279	0.797620
1	-0.071909	2.679900	0.614813
1	-0.713180	3.982464	0.144189
8	3.379926	-1.121544	-0.484349

```
  1      2.534535     -1.185488     -0.970116
  1      3.936598     -1.809628     -0.853301
  8      0.760495     -1.010551     -1.467978
  1      0.705956     -0.170723     -0.982756
  1      0.424958     -1.664581     -0.833059
  8     -0.213117     -2.756958      0.596568
  1     -1.129399     -2.421589      0.510790
  1     -0.279184     -3.712787      0.581189
  8      1.637690     -0.958811      1.787383
  1      0.962297     -1.588347      1.487434
  1      2.369490     -1.119650      1.172807
 ---------------------------------------------------

Cartesian coordinates of :  IMZW7_18

 ---------------------------------------------------
 Atomic number (AN) and Cartesian coordinates
  AN          X             Y             Z
 ---------------------------------------------------
  6      3.410699     -0.753587      0.632873
  6      3.299030      0.590061      0.839445
  6      1.917154     -0.054293     -0.727126
  1      4.060014     -1.451856      1.132370
  1      3.798111      1.262282      1.512940
  1      1.955578      1.960347     -0.133622
  1      1.156026      0.007246     -1.487186
  7      2.545468     -1.155131     -0.357002
  7      2.345182      1.021187     -0.041406
  8     -1.801358     -1.211844     -1.635331
  1     -1.094676     -1.785342     -1.298799
  1     -1.512893     -0.319652     -1.382625
  8      0.729652      3.373863     -0.390355
  1      0.824715      3.662999     -1.299465
  1      0.003800      2.720254     -0.421315
  8     -0.590356     -0.786020      1.476684
  1     -1.547035     -0.938684      1.480031
  1     -0.267238     -1.484692      0.883783
  8     -3.330957     -1.125064      0.707456
  1     -2.914239     -1.285032     -0.162090
  1     -3.976940     -1.825628      0.813380
  8     -3.817184      1.592079      0.344577
  1     -3.804006      0.638583      0.545592
  1     -3.836914      2.012983      1.205980
  8      0.250563     -2.776626     -0.442314
  1      0.317489     -3.685769     -0.146811
  1      1.165088     -2.429330     -0.446039
  8     -1.122312      1.306496     -0.391094
  1     -2.040178      1.518009     -0.134664
  1     -0.826940      0.684731      0.299252
 ---------------------------------------------------

Cartesian coordinates of :  IMZW7_19

 ---------------------------------------------------
```

Atomic number (AN) and Cartesian coordinates

AN	X	Y	Z
6	2.972819	-0.731110	0.759253
6	2.341818	-1.849051	0.295856
6	1.940298	-0.142690	-1.020394
1	3.593043	-0.632614	1.633310
1	2.295724	-2.859866	0.657247
1	1.104037	-2.028572	-1.421402
1	1.543337	0.416332	-1.850177
7	2.722030	0.335337	-0.073058
7	1.696428	-1.455776	-0.844707
8	1.733068	3.008313	-0.007662
1	2.236390	2.170186	0.009584
1	1.953169	3.396670	-0.856387
8	-3.480224	-0.633381	-0.671966
1	-4.398780	-0.776279	-0.904545
1	-3.486450	0.046385	0.030860
8	-1.535943	-2.253911	0.661956
1	-1.039620	-1.542210	1.100028
1	-2.247942	-1.788997	0.198990
8	-1.261877	-0.081584	-2.237648
1	-2.119202	-0.274556	-1.816089
1	-1.487846	0.454815	-2.999708
8	-0.075701	-0.163199	1.950512
1	0.825660	-0.477599	1.822792
1	-0.212019	0.472538	1.222298
8	-0.716151	1.657837	-0.093543
1	-0.776501	1.120472	-0.904226
1	0.108762	2.175009	-0.160655
8	-3.076898	1.233493	1.333333
1	-2.239623	1.506754	0.921531
1	-2.801138	0.661175	2.052763

Cartesian coordinates of : IMZW7_20

Atomic number (AN) and Cartesian coordinates

AN	X	Y	Z
6	-3.346597	-0.842473	-0.207458
6	-3.379522	0.499503	-0.450720
6	-1.340957	-0.137160	0.024702
1	-4.163980	-1.542379	-0.229345
1	-4.179455	1.167572	-0.711450
1	-1.710946	1.869501	-0.385593
1	-0.280376	-0.069272	0.205753
7	-2.064936	-1.238636	0.090959
7	-2.090883	0.931578	-0.298060
8	1.831353	-0.645824	1.681244
1	1.282862	-1.391033	1.395318
1	2.415919	-0.457883	0.925353
8	0.431495	1.693704	2.253193

1	0.913430	0.869256	2.042280
1	1.057741	2.207453	2.765966
8	3.566546	0.015942	-0.455976
1	4.254699	0.589460	-0.114306
1	2.973827	0.601394	-0.955504
8	1.382192	-1.714721	-1.516231
1	0.931628	-2.120368	-0.753426
1	2.279658	-1.573662	-1.198926
8	-0.037163	2.954755	-0.222153
1	0.424523	2.351055	-0.826490
1	0.152044	2.573448	0.654827
8	1.348372	1.073183	-1.823613
1	1.438257	1.253626	-2.761334
1	1.131652	0.123528	-1.772336
8	0.134414	-2.778759	0.779788
1	-0.775798	-2.472775	0.580486
1	0.089822	-3.734987	0.819273

Cartesian coordinates of : IMZW7_21

Atomic number (AN) and Cartesian coordinates

AN	X	Y	Z
6	3.341331	-0.740832	0.813843
6	3.247376	0.607564	0.998495
6	1.985498	-0.020421	-0.673542
1	3.927958	-1.449289	1.372971
1	3.705530	1.274706	1.705225
1	2.028840	1.993856	-0.115508
1	1.292548	0.053856	-1.495483
7	2.552806	-1.131569	-0.242013
7	2.382068	1.051028	0.036571
8	-3.699242	1.630270	0.737169
1	-4.299426	1.884129	0.033788
1	-2.818938	1.629655	0.314694
8	-3.353021	-1.121648	0.725135
1	-3.643085	-0.190811	0.782915
1	-4.085268	-1.642463	1.058398
8	-0.606580	-0.954605	1.403004
1	-0.266599	-1.550215	0.716802
1	-1.567897	-1.075854	1.333915
8	-1.855573	-1.112453	-1.693990
1	-1.099336	-1.662842	-1.439807
1	-2.461224	-1.209236	-0.941147
8	-1.142604	1.262100	-0.327927
1	-1.354694	0.600354	-1.011177
1	-0.807513	0.689846	0.384792
8	0.758369	3.234985	-0.859703
1	0.046882	2.586122	-0.702721
1	0.570409	3.942554	-0.240996
8	0.313445	-2.735065	-0.753778
1	0.310400	-3.627671	-0.405044

1	1.213714	-2.389439	-0.585585

Cartesian coordinates of : IMZW7_22

Atomic number (AN) and Cartesian coordinates

AN	X	Y	Z
6	2.951104	-0.985089	0.482429
6	1.783097	-1.686903	0.523201
6	1.862721	-0.448561	-1.277577
1	3.756765	-0.991303	1.196481
1	1.382652	-2.390474	1.230535
1	0.146077	-1.621564	-0.822661
1	1.553776	-0.003040	-2.207315
7	3.003376	-0.211720	-0.655065
7	1.106017	-1.343818	-0.615780
8	-3.376425	-0.057819	-1.187026
1	-4.081118	0.069353	-1.824220
1	-2.611407	0.443318	-1.537939
8	-1.023597	1.205120	-1.787721
1	-0.652899	1.274181	-0.885789
1	-1.167909	2.115901	-2.051344
8	-1.641740	-2.164434	-0.418377
1	-1.538769	-1.838662	0.492838
1	-2.302762	-1.562208	-0.793600
8	-0.029468	1.487611	0.814331
1	0.040444	0.644905	1.278430
1	0.885274	1.813083	0.705316
8	2.519967	2.494746	0.279444
1	2.878257	1.662365	-0.073701
1	3.005256	2.631168	1.095452
8	-1.201501	-0.882887	2.055919
1	-1.898602	-0.209379	1.940905
1	-1.381763	-1.286791	2.906685
8	-2.885057	1.217364	1.290840
1	-3.199643	0.818204	0.462295
1	-2.058339	1.645429	1.035955

Cartesian coordinates of : IMZW8_ 1

Atomic number (AN) and Cartesian coordinates

AN	X	Y	Z
6	-3.075675	-0.879484	0.370667
6	-3.217412	0.408483	-0.054385
6	-1.105167	-0.044736	0.308459
1	-3.843368	-1.617297	0.527188
1	-4.078391	0.990992	-0.325543
1	-1.664769	1.866179	-0.320934
1	-0.038150	0.100810	0.368053

7	-1.749974	-1.158272	0.599314
7	-1.951284	0.924866	-0.086409
8	1.524208	-2.194137	-1.070858
1	2.041471	-1.395785	-0.868802
1	1.152024	-2.460159	-0.213655
8	2.817478	0.283493	-0.555959
1	3.697493	0.333515	-0.931969
1	2.231103	0.749229	-1.190560
8	0.622306	1.952064	2.353022
1	1.254956	2.501432	2.819057
1	1.074913	1.089831	2.259784
8	0.989097	1.471457	-2.219937
1	0.559513	1.987290	-1.514720
1	0.418871	0.695194	-2.365584
8	-0.020846	3.021991	-0.113611
1	0.280565	3.930035	-0.164800
1	0.279449	2.697180	0.759957
8	1.984879	-0.458723	2.033986
1	1.419410	-1.239242	1.911860
1	2.316301	-0.261716	1.143784
8	-0.367463	-0.966682	-2.710503
1	0.257471	-1.457818	-2.139786
1	-0.087661	-1.179721	-3.602595
8	0.368156	-2.703525	1.453962
1	0.197413	-3.523300	1.919403
1	-0.512801	-2.325886	1.230267

Cartesian coordinates of : IMZW8_ 2

Atomic number (AN) and Cartesian coordinates

AN	X	Y	Z
6	-1.685086	2.395663	-1.128973
6	-2.605469	1.734728	-0.368876
6	-0.753111	0.576691	-0.499157
1	-1.789762	3.346638	-1.622221
1	-3.613472	1.973237	-0.083178
1	-2.373674	-0.190965	0.573565
1	-0.051273	-0.224255	-0.328453
7	-0.524757	1.664095	-1.212031
7	-1.994265	0.575693	0.022506
8	2.783843	1.067100	0.730123
1	2.059014	1.432560	1.276465
1	3.592315	1.391575	1.129912
8	0.039478	-0.886876	2.506238
1	0.720058	-1.219860	1.882956
1	0.297046	-1.237636	3.361116
8	-1.230318	-2.719370	-1.275572
1	-1.872900	-2.222984	-1.786543
1	-0.379332	-2.322586	-1.529350
8	2.016246	-1.635874	0.731127
1	1.730876	-1.765659	-0.189559

```
  1      2.395212     -0.738997      0.726037
  8     -2.228215     -2.023025      1.286758
  1     -1.460097     -1.714490      1.797251
  1     -1.840493     -2.310898      0.441495
  8      1.274643     -1.685041     -2.008873
  1      1.869300     -2.200394     -2.556098
  1      1.581299     -0.756337     -2.090845
  8      0.631766      1.844210      2.307004
  1      0.338586      0.918627      2.407982
  1     -0.038482      2.246640      1.750180
  8      2.094402      0.953195     -2.008280
  1      1.209295      1.351693     -1.887298
  1      2.466882      1.008530     -1.112901
 ----------------------------------------------------

Cartesian coordinates of :  IMZW8_ 3

 ----------------------------------------------------
 Atomic number (AN) and Cartesian coordinates
  AN          X             Y             Z
 ----------------------------------------------------
  6     -2.915216     -0.161579     -1.204779
  6     -2.707917      1.183829     -1.125156
  6     -0.905512      0.099661     -0.519609
  1     -3.806804     -0.680504     -1.511940
  1     -3.338884      2.027510     -1.335780
  1     -0.927478      2.191554     -0.470016
  1      0.099036     -0.066721     -0.163982
  7     -1.779936     -0.839353     -0.829521
  7     -1.418521      1.331731     -0.691440
  8     -0.709786     -2.655793      0.978236
  1     -1.101801     -3.521527      1.108194
  1     -1.239299     -2.236116      0.266847
  8      2.383072      1.403298     -1.016704
  1      2.580006      0.789306     -0.288205
  1      1.763336      2.035653     -0.615711
  8      2.633603     -0.510687      1.032078
  1      2.344853     -1.278270      0.493523
  1      3.456985     -0.778773      1.442773
  8      1.652998     -2.486616     -0.605358
  1      1.443329     -1.887118     -1.344969
  1      0.813588     -2.617036     -0.133986
  8      0.559172      3.032072      0.418075
  1      0.816870      3.891476      0.754349
  1      0.614016      2.422089      1.184766
  8      0.758992      1.105426      2.369779
  1      1.391566      0.500253      1.946778
  1     -0.059956      0.582114      2.440799
  8      1.342487     -0.586177     -2.683564
  1      2.068615     -0.782310     -3.278482
  1      1.669767      0.162393     -2.146736
  8     -1.559273     -0.494943      2.651119
  1     -2.231263     -0.098551      2.091135
  1     -1.285541     -1.286178      2.155335
```

Cartesian coordinates of : IMZW8_ 4

Atomic number (AN) and Cartesian coordinates

AN	X	Y	Z
6	-3.660388	0.714637	0.008807
6	-3.656461	-0.649370	0.009909
6	-1.606332	0.115822	-0.015117
1	-4.507956	1.377897	0.018529
1	-4.448847	-1.374854	0.020395
1	-1.929752	-1.943334	-0.007958
1	-0.528133	0.109169	-0.027777
7	-2.371773	1.190959	-0.006980
7	-2.338333	-1.012727	-0.005822
8	3.452591	0.983579	0.039190
1	3.350927	0.008768	0.033157
1	4.395939	1.151175	0.038029
8	0.753639	-1.267853	2.045895
1	1.103889	-1.479377	2.912864
1	0.842342	-0.296021	1.966203
8	-0.276698	-3.012569	-0.007069
1	0.079112	-2.519733	-0.761126
1	0.065490	-2.519599	0.752876
8	1.255979	1.445012	1.749373
1	2.080237	1.379222	1.240521
1	0.685954	2.005105	1.195963
8	0.785649	-1.284281	-2.051591
1	1.146243	-1.505278	-2.911897
1	0.889213	-0.313559	-1.973958
8	1.318877	1.417407	-1.749193
1	2.124832	1.354069	-1.210778
1	0.731591	1.984941	-1.222851
8	2.728438	-1.658377	0.017942
1	2.136271	-1.608052	-0.751664
1	2.116269	-1.601411	0.771698
8	-0.292813	2.995519	-0.038532
1	-0.451136	3.940324	-0.033298
1	-1.177954	2.568898	-0.029031

Cartesian coordinates of : IMZW8_ 5

Atomic number (AN) and Cartesian coordinates

AN	X	Y	Z
6	-2.876018	-1.233938	0.915892
6	-3.211532	0.088728	0.974615
6	-1.945781	-0.297826	-0.767560
1	-3.159297	-2.022950	1.590881
1	-3.805736	0.653467	1.669263

1	-2.570400	1.652305	-0.361498
1	-1.383274	-0.103727	-1.665660
7	-2.087614	-1.473296	-0.184874
7	-2.619251	0.666655	-0.114383
8	1.671951	-1.735251	1.102682
1	2.428302	-1.301541	0.672739
1	1.205402	-2.167682	0.367590
8	1.501500	-0.347195	-1.996838
1	2.346536	-0.415879	-1.525160
1	1.043944	-1.173394	-1.773034
8	0.650783	1.709931	-0.273213
1	0.274122	1.233332	0.492126
1	0.881397	0.995514	-0.899728
8	3.731907	-0.359475	-0.267283
1	4.657608	-0.606231	-0.251652
1	3.664452	0.464693	0.254295
8	-1.580765	3.135267	-1.185720
1	-1.692602	2.932413	-2.116353
1	-0.757631	2.670529	-0.940307
8	3.064289	1.907406	1.157193
1	2.806732	1.537835	2.004655
1	2.227071	1.937689	0.656354
8	0.216824	-2.724591	-1.104245
1	0.162817	-3.631031	-1.409895
1	-0.680692	-2.491336	-0.781940
8	-0.023090	0.255484	2.022738
1	0.546178	-0.482717	1.721242
1	-0.914096	-0.109197	2.010688

Cartesian coordinates of : IMZW8_ 6

Atomic number (AN) and Cartesian coordinates

AN	X	Y	Z
6	-3.166407	0.301808	0.282271
6	-2.948927	-0.950638	0.778083
6	-1.047821	0.098632	0.495933
1	-4.106739	0.768505	0.044390
1	-3.615444	-1.749702	1.045733
1	-1.054555	-1.841667	1.264564
1	0.017004	0.270887	0.496095
7	-1.971998	0.959262	0.111721
7	-1.592681	-1.059839	0.913204
8	0.879184	2.390755	2.035367
1	0.595882	2.630108	1.130658
1	1.538855	3.049829	2.258170
8	0.792486	-2.357900	1.746819
1	1.294786	-3.053069	2.174192
1	1.312929	-1.539328	1.887220
8	-0.141359	3.033998	-0.495506
1	-0.933146	2.488777	-0.320916
1	0.301755	2.523244	-1.193205

8	2.661986	-0.161080	-0.898829
1	2.205404	-1.025098	-1.003930
1	3.543352	-0.296610	-1.251812
8	1.122505	-2.424587	-1.063180
1	0.347308	-2.013929	-1.486556
1	0.916268	-2.456990	-0.112706
8	-0.837158	-1.091394	-2.597104
1	-0.342951	-0.251345	-2.567225
1	-1.651464	-0.903236	-2.125362
8	0.804120	1.195212	-2.424011
1	1.150261	1.476498	-3.272913
1	1.558115	0.768578	-1.970815
8	2.287229	-0.032387	1.892541
1	1.771776	0.792281	1.972355
1	2.530340	-0.053442	0.951097

Cartesian coordinates of : IMZW8_ 7

Atomic number (AN) and Cartesian coordinates

AN	X	Y	Z
6	-0.571250	2.738378	-0.572636
6	0.784908	2.585608	-0.526408
6	-0.156287	0.759389	-1.276516
1	-1.155360	3.600089	-0.299239
1	1.576674	3.244534	-0.219679
1	1.940516	0.849901	-0.999323
1	-0.250171	-0.242902	-1.659546
7	-1.156883	1.591452	-1.051954
7	1.032464	1.322913	-0.991148
8	-2.475108	-0.515241	2.030539
1	-2.878561	-0.309399	1.168395
1	-2.156810	-1.415084	1.925525
8	-3.392747	0.049657	-0.553852
1	-2.721047	0.723589	-0.810852
1	-4.232412	0.361925	-0.893424
8	2.687810	-0.047622	2.064695
1	1.750364	0.205306	2.096821
1	2.690421	-0.954911	2.376133
8	1.774853	-2.248286	-1.562573
1	1.244941	-2.163328	-0.745452
1	2.192905	-3.107582	-1.483576
8	0.218729	-1.931787	0.732983
1	-0.616803	-2.020152	0.233122
1	0.230466	-1.008925	1.017908
8	-0.023718	0.779496	2.128158
1	-0.916901	0.379447	2.108321
1	-0.088132	1.546287	1.548986
8	3.455096	-0.183232	-0.649301
1	3.233367	-0.184281	0.299731
1	2.995600	-0.969523	-0.994014
8	-2.017017	-2.348848	-0.879443

```
  1     -2.554617     -1.536354     -0.870304
  1     -1.580920     -2.332538     -1.734095
 ---------------------------------------------------

Cartesian coordinates of :  IMZW8_ 8

 ---------------------------------------------------
 Atomic number (AN) and Cartesian coordinates
  AN          X             Y             Z
 ---------------------------------------------------
  6      2.232733     -2.187131     -0.020230
  6      2.922276     -1.016707     -0.159977
  6      1.067348     -0.674635      0.945881
  1      2.504609     -3.166642     -0.373850
  1      3.857465     -0.781350     -0.634424
  1      2.352222      0.940407      0.474192
  1      0.298054     -0.153146      1.488441
  7      1.072419     -1.968128      0.681825
  7      2.171756     -0.064982      0.475240
  8     -2.588128      0.211493      1.527480
  1     -2.785249      0.522741      0.630108
  1     -2.262941     -0.695214      1.405839
  8     -2.801946      1.248373     -1.100649
  1     -3.416602      1.816606     -1.567175
  1     -1.933771      1.686258     -1.163292
  8     -0.118942      2.064693     -0.921338
  1      0.155146      1.191600     -1.261191
  1     -0.233237      1.957630      0.042736
  8     -2.020748     -1.420250     -1.570513
  1     -1.969377     -1.798235     -0.676468
  1     -2.408939     -0.539560     -1.441005
  8      0.439426     -0.341104     -2.272910
  1      1.151272     -0.872836     -1.902486
  1     -0.377649     -0.810722     -2.009132
  8     -1.647313     -2.417034      1.046839
  1     -1.953908     -3.268078      1.362436
  1     -0.671311     -2.488300      0.972757
  8      2.457212      2.799259      0.088736
  1      1.631109      2.717464     -0.415651
  1      3.133567      2.902583     -0.583472
  8     -0.569790      2.099078      1.850754
  1     -1.017816      2.942929      1.935283
  1     -1.294181      1.442402      1.835659
 ---------------------------------------------------

Cartesian coordinates of :  IMZW8_ 9

 ---------------------------------------------------
 Atomic number (AN) and Cartesian coordinates
  AN          X             Y             Z
 ---------------------------------------------------
  6     -1.553928     -2.692908     -0.055744
  6     -2.643369     -1.946331      0.284264
  6     -1.153133     -0.694249     -0.711653
```

1	-1.384271	-3.742193	0.114444
1	-3.564039	-2.196207	0.778711
1	-2.952369	0.160441	-0.055438
1	-0.693748	0.183247	-1.137605
7	-0.620166	-1.904514	-0.688364
7	-2.372020	-0.676651	-0.146274
8	3.565805	-0.727142	0.274768
1	3.091441	-0.912823	-0.562969
1	4.447660	-1.085176	0.163812
8	1.101533	1.683940	-1.782742
1	1.646248	1.794280	-0.973345
1	1.489737	2.280951	-2.425357
8	-0.870432	2.694892	0.080903
1	-0.568834	2.096987	0.782988
1	-0.330411	2.432442	-0.680133
8	2.416056	1.812481	0.625854
1	1.659210	1.583992	1.190817
1	2.929271	0.988974	0.576784
8	-3.533856	1.929792	-0.035581
1	-3.891120	2.109423	-0.906843
1	-2.615337	2.263791	-0.075938
8	0.111648	0.931220	2.092175
1	0.212311	1.152523	3.019385
1	0.476170	0.026250	2.001390
8	1.897216	-1.061380	-1.878786
1	1.605147	-0.136678	-1.951663
1	1.128866	-1.517395	-1.491869
8	1.247349	-1.550164	1.649986
1	0.750324	-1.791895	0.856101
1	2.129078	-1.330948	1.305382

Cartesian coordinates of : IMZW8_10

Atomic number (AN) and Cartesian coordinates

AN	X	Y	Z
6	2.945393	-1.395204	-0.543105
6	3.298783	-0.092560	-0.753249
6	1.888143	-0.240041	0.911540
1	3.276531	-2.268269	-1.078652
1	3.955563	0.372690	-1.465282
1	2.562296	1.641718	0.284276
1	1.252944	0.073894	1.723004
7	2.064365	-1.484275	0.508749
7	2.624202	0.628720	0.193560
8	-1.556657	-1.948905	-1.119980
1	-1.165564	-2.290243	-0.286993
1	-1.707820	-2.724029	-1.664213
8	-3.220622	2.131247	-0.583439
1	-3.346558	2.283239	-1.521636
1	-3.465981	1.196712	-0.455932
8	-1.613830	-0.052698	1.914356

1	-1.136406	0.531737	1.298463
1	-2.432063	-0.246141	1.430698
8	-3.658903	-0.552247	0.005927
1	-3.040777	-1.132345	-0.478274
1	-4.496029	-1.018530	0.018018
8	1.644884	3.325920	0.556428
1	0.807431	2.859822	0.372471
1	1.639903	3.448823	1.507298
8	-0.523554	1.695198	-0.043842
1	-1.432342	1.942340	-0.302995
1	-0.212558	1.109681	-0.760622
8	-0.460323	-2.557571	1.339147
1	-0.808949	-1.696763	1.640064
1	0.460325	-2.353230	1.087261
8	0.246951	-0.064952	-2.095437
1	-0.328348	-0.796505	-1.802210
1	1.140949	-0.386827	-1.937308

Cartesian coordinates of : IMZW8_11

Atomic number (AN) and Cartesian coordinates

AN	X	Y	Z
6	-0.162485	-2.944271	0.721657
6	1.183810	-2.770946	0.592656
6	0.161225	-0.843773	0.454875
1	-0.706594	-3.860850	0.871179
1	2.005599	-3.462963	0.609045
1	2.262150	-0.928532	0.343310
1	0.018777	0.217936	0.335218
7	-0.802259	-1.730607	0.636844
7	1.371932	-1.426171	0.422062
8	-0.790388	1.970779	2.031043
1	-1.481287	1.279342	2.079945
1	-1.174369	2.736987	2.460963
8	3.673680	0.285990	0.392425
1	3.072326	1.004935	0.663337
1	4.172619	0.080736	1.185103
8	-0.137063	-0.842658	-2.967435
1	0.309285	-0.028437	-2.671252
1	-0.266637	-0.712056	-3.908453
8	-2.484436	-0.421868	-1.466653
1	-2.210438	-0.949790	-0.703516
1	-1.744624	-0.563146	-2.086985
8	-2.733579	-0.011620	1.895311
1	-2.191213	-0.712847	1.486152
1	-3.285075	0.301266	1.174542
8	1.758221	2.282589	0.749662
1	0.940415	2.104863	1.238424
1	1.517226	2.096396	-0.173156
8	-1.511244	2.140562	-0.766411
1	-1.906135	1.259991	-0.912531

1	-1.261223	2.145137	0.172419
8	0.928708	1.528259	-1.877825
1	1.174667	2.174375	-2.543020
1	0.040330	1.811783	-1.569128

Cartesian coordinates of : IMZW8_12

Atomic number (AN) and Cartesian coordinates

AN	X	Y	Z
6	-0.928898	-2.645710	-0.315196
6	-2.072056	-2.046042	-0.759041
6	-1.207863	-0.898281	0.889583
1	-0.464772	-3.547956	-0.674960
1	-2.767293	-2.303099	-1.536998
1	-2.985292	-0.239181	-0.045226
1	-1.098147	-0.122655	1.628885
7	-0.393988	-1.924419	0.725031
7	-2.238039	-0.936880	0.024569
8	2.231131	-1.724345	1.545733
1	2.504891	-2.375421	2.192803
1	1.284898	-1.919877	1.338227
8	1.934689	1.035256	1.798496
1	2.682086	1.376362	1.297354
1	2.080048	0.071906	1.806292
8	0.346947	1.799319	-0.393194
1	0.278894	1.048557	-1.014051
1	0.752826	1.416669	0.407489
8	3.159774	2.081640	-0.715908
1	2.200789	2.193554	-0.819933
1	3.544810	2.488276	-1.494146
8	0.506940	-0.315312	-2.227955
1	-0.094061	-1.029072	-1.990785
1	1.356619	-0.572840	-1.816553
8	-2.024085	2.792644	0.652474
1	-1.215791	2.435730	0.233871
1	-2.123612	3.665805	0.269528
8	2.970447	-0.740756	-1.001708
1	2.817553	-1.110010	-0.117668
1	3.109869	0.210307	-0.851196
8	-4.131473	1.189602	-0.125941
1	-3.432913	1.814983	0.155603
1	-4.709276	1.122866	0.636210

Cartesian coordinates of : IMZW8_13

Atomic number (AN) and Cartesian coordinates

AN	X	Y	Z
6	2.629440	-1.727174	0.051522

```
  6      1.465895     -2.441574      0.074033
  6      1.166428     -0.538318     -0.962118
  1      3.587176     -2.000268      0.459887
  1      1.215577     -3.403528      0.482744
  1     -0.447691     -1.877960     -0.695436
  1      0.666088      0.253454     -1.494667
  7      2.437696     -0.536363     -0.606373
  7      0.547646     -1.674629     -0.588921
  8      0.788106     -0.096249      2.389358
  1      0.904111      0.717309      1.861491
  1      1.344458     -0.748608      1.950925
  8     -1.355270      2.147929     -0.744638
  1     -2.088529      1.870462     -0.166821
  1     -0.570261      2.160540     -0.170112
  8      0.918853      2.252399      0.903444
  1      0.914034      3.031850      1.462146
  1      1.748343      2.314038      0.385514
  8     -2.349074     -1.911125     -0.641945
  1     -2.271171     -1.503908      0.236160
  1     -2.328887     -1.161017     -1.264456
  8      3.257134      2.143761     -0.568932
  1      3.109110      1.176122     -0.659393
  1      3.085303      2.494529     -1.444579
  8     -1.958354      0.130882     -2.534210
  1     -1.171753     -0.238145     -2.941100
  1     -1.618389      0.834276     -1.945838
  8     -3.531468      1.129755      0.723442
  1     -3.926205      0.608149      0.020976
  1     -2.977170      0.487210      1.203119
  8     -1.842198     -0.745228      1.891802
  1     -2.161233     -1.179977      2.684256
  1     -0.902724     -0.532183      2.075061
 ---------------------------------------------------

Cartesian coordinates of :  IMZW8_14

 ---------------------------------------------------
 Atomic number (AN) and Cartesian coordinates
  AN          X             Y             Z
 ---------------------------------------------------
  6     -1.246414      2.486165      0.121904
  6      0.114696      2.420698      0.028985
  6     -0.800652      0.809449     -1.131984
  1     -1.844964      3.197919      0.664082
  1      0.897326      3.029045      0.445015
  1      1.308799      1.033959     -1.076422
  1     -0.879693     -0.053372     -1.772365
  7     -1.816297      1.475493     -0.614908
  7      0.380564      1.351440     -0.780484
  8     -2.349837     -1.744943      0.704796
  1     -3.002146     -1.279718      0.141926
  1     -2.860532     -2.378691      1.211416
  8      1.547665     -1.861949     -2.255448
  1      2.106613     -2.614745     -2.454569
```

1	1.058021	-2.130709	-1.450950
8	2.981513	0.425170	-1.584820
1	3.258881	0.431161	-0.652317
1	2.599530	-0.457886	-1.743562
8	0.255059	-2.600100	0.059152
1	0.757214	-2.116122	0.736499
1	-0.660774	-2.304326	0.190686
8	3.507520	0.838954	1.157964
1	2.820060	0.236747	1.501354
1	3.086311	1.700789	1.171377
8	1.580033	-0.985511	1.963076
1	1.838667	-1.451751	2.760369
1	0.712771	-0.578896	2.178161
8	-4.053244	-0.190899	-0.823595
1	-4.039610	-0.502423	-1.730389
1	-3.359582	0.506355	-0.795962
8	-0.936001	0.032877	2.358587
1	-0.950073	0.886280	1.912740
1	-1.464136	-0.548808	1.779534

Cartesian coordinates of : IMZW8_15

Atomic number (AN) and Cartesian coordinates

AN	X	Y	Z
6	1.878896	2.375051	-0.596701
6	0.811009	2.918634	0.054307
6	0.358581	0.866698	-0.555677
1	2.831742	2.827837	-0.810611
1	0.648254	3.885506	0.493722
1	-1.095712	1.992984	0.449014
1	-0.199149	-0.047235	-0.682858
7	1.589543	1.087260	-0.984521
7	-0.148089	1.943932	0.069595
8	-1.784112	-0.819671	2.052207
1	-0.883364	-1.201911	1.965583
1	-2.239705	-1.387609	2.675461
8	-2.871120	1.488816	0.843777
1	-2.566179	0.680989	1.286543
1	-3.015494	1.205653	-0.076678
8	2.856297	-1.397405	-1.381166
1	3.219184	-1.583503	-0.512213
1	2.506527	-0.482725	-1.298370
8	-2.219220	-1.812811	-0.606119
1	-1.314433	-2.083068	-0.828843
1	-2.149276	-1.492222	0.308785
8	0.422869	-2.679855	-1.168376
1	1.300033	-2.271831	-1.324728
1	0.577994	-3.625377	-1.198697
8	2.622467	0.065148	1.823636
1	2.658709	0.340447	0.901081
1	2.159902	0.790893	2.250378

```
  8     -2.958869      0.558809     -1.831694
  1     -2.697303     -0.320010     -1.490130
  1     -3.865384      0.438868     -2.119937
  8      0.658520     -1.952353      1.605552
  1      1.341504     -1.258359      1.635515
  1      0.586399     -2.195394      0.667369
 ---------------------------------------------------

Cartesian coordinates of :  IMZW8_16

 ---------------------------------------------------
 Atomic number (AN) and Cartesian coordinates
  AN          X             Y             Z
 ---------------------------------------------------
  6     -0.363758      2.631601     -0.180070
  6      0.994246      2.588356     -0.309678
  6      0.114741      0.700051     -0.977782
  1     -0.976481      3.436474      0.188245
  1      1.763526      3.306120     -0.091313
  1      2.207765      0.956436     -0.991468
  1      0.052742     -0.297820     -1.377867
  7     -0.912687      1.446420     -0.609380
  7      1.278662      1.356187     -0.833319
  8     -3.096447      0.738819      1.133761
  1     -3.725036      0.212465      0.634664
  1     -2.430603      0.998127      0.468646
  8      1.032764     -2.667342     -0.933357
  1      0.063401     -2.579762     -0.835052
  1      1.200504     -3.602895     -0.807567
  8      2.456854     -1.346465      1.083376
  1      1.923632     -0.636093      1.480233
  1      1.859495     -1.791190      0.453526
  8     -1.694993     -2.339906     -0.510777
  1     -2.077148     -1.618688     -1.041773
  1     -1.572057     -1.950659      0.371343
  8      3.756012     -0.069132     -1.005248
  1      4.473202      0.477986     -0.680180
  1      3.423488     -0.532898     -0.209954
  8     -1.235839     -1.099944      1.998750
  1     -1.487740     -1.605708      2.774110
  1     -1.978515     -0.480962      1.844838
  8     -2.850131     -0.238568     -2.044704
  1     -2.445389     -0.325963     -2.910353
  1     -2.340192      0.471703     -1.622545
  8      0.905045      0.640128      2.415085
  1      0.736364      1.399750      1.848647
  1      0.109722      0.084555      2.312807
 ---------------------------------------------------

Cartesian coordinates of :  IMZW8_17

 ---------------------------------------------------
 Atomic number (AN) and Cartesian coordinates
  AN          X             Y             Z
```

6	-3.244194	0.309122	-0.885286
6	-2.816550	-0.982337	-0.789667
6	-2.194530	0.157330	0.970529
1	-3.805708	0.766467	-1.681719
1	-2.918451	-1.830190	-1.442491
1	-1.622435	-1.860594	0.751095
1	-1.731313	0.372020	1.919048
7	-2.858665	1.022377	0.225883
7	-2.161326	-1.065643	0.408504
8	1.130325	-0.411756	1.447386
1	0.679915	-0.192835	0.614700
1	1.960837	-0.801016	1.127577
8	0.813550	-2.263947	-1.364995
1	1.708358	-2.073146	-1.039437
1	0.406068	-1.386766	-1.375369
8	1.673812	2.396725	1.291384
1	0.849249	2.650738	0.848973
1	1.517449	1.469158	1.534179
8	3.058906	1.313783	-0.871907
1	2.705689	1.840603	-0.128368
1	3.556667	1.926900	-1.414779
8	-0.783076	2.952995	-0.072993
1	-0.955311	3.620419	-0.739279
1	-1.630444	2.483859	0.047620
8	-0.132363	-3.009707	1.199400
1	0.288942	-2.237163	1.603512
1	0.168214	-2.923825	0.276359
8	0.347641	0.575734	-1.244408
1	1.289776	0.809987	-1.283564
1	-0.074079	1.385190	-0.910225
8	3.239068	-1.360189	-0.182814
1	3.380482	-0.444263	-0.479814
1	4.114773	-1.726482	-0.050608

Cartesian coordinates of : IMZW8_18

Atomic number (AN) and Cartesian coordinates

AN	X	Y	Z
6	0.571994	-2.903387	-0.370068
6	-0.782698	-2.940335	-0.231658
6	-0.076611	-0.872374	-0.129176
1	1.254307	-3.723787	-0.510696
1	-1.485711	-3.752863	-0.227399
1	-2.137979	-1.303841	0.053418
1	-0.102556	0.200453	-0.031144
7	1.012475	-1.602056	-0.305965
7	-1.179025	-1.638886	-0.078721
8	2.915637	-0.556301	1.655484
1	2.566711	-0.829373	2.506621
1	2.335104	-0.999317	1.012225

8	-1.410292	1.286478	-2.241427
1	-1.774612	1.985662	-2.787247
1	-0.495949	1.575619	-2.061279
8	-2.425048	1.848902	0.343432
1	-2.001881	1.662338	-0.513818
1	-1.782570	1.565552	1.019324
8	1.636800	1.947649	1.351595
1	1.445568	2.042467	0.403100
1	2.136134	1.113457	1.416230
8	-3.800188	-0.561803	0.373562
1	-3.426253	0.342463	0.358970
1	-4.297439	-0.624081	-0.444015
8	2.968087	0.038060	-1.596993
1	2.385198	-0.609086	-1.150711
1	3.602645	0.281042	-0.919295
8	1.127876	2.085933	-1.441630
1	1.472028	2.879782	-1.855421
1	1.811852	1.401207	-1.597772
8	-0.732829	1.010983	2.449160
1	0.108937	1.368237	2.098747
1	-0.964138	1.608033	3.162919

Cartesian coordinates of : IMZW8_19

Atomic number (AN) and Cartesian coordinates

AN	X	Y	Z
6	-2.464502	-2.000396	0.153866
6	-2.853402	-0.693541	0.228687
6	-1.149497	-0.901918	-1.124912
1	-2.890444	-2.851110	0.657645
1	-3.637497	-0.202538	0.775618
1	-1.986270	1.001260	-0.766259
1	-0.371624	-0.623315	-1.815495
7	-1.397645	-2.129314	-0.705064
7	-2.008529	-0.008183	-0.600237
8	1.247444	-2.668627	0.131128
1	1.593129	-3.563314	0.143439
1	0.349074	-2.743427	-0.246590
8	0.947189	1.586355	-1.644672
1	0.896734	1.400485	-0.685523
1	1.649897	2.236084	-1.720408
8	0.985971	1.037848	1.086888
1	0.605266	0.231346	1.484591
1	1.949947	0.884817	1.080450
8	3.682700	0.496072	0.637748
1	3.402842	-0.021687	-0.142142
1	3.990259	-0.164829	1.260896
8	-1.589685	2.809624	-1.105232
1	-1.369152	3.007217	-0.176167
1	-0.733651	2.540692	-1.473715
8	-0.820130	3.085693	1.588567

1	-0.090856	2.446322	1.497828
1	-0.382652	3.926532	1.732524
8	2.567431	-0.828393	-1.574069
1	1.950679	-0.091943	-1.707665
1	2.069683	-1.471846	-1.040112
8	-0.009191	-1.289364	2.267802
1	0.477978	-1.878230	1.667000
1	-0.919553	-1.358588	1.960079

Cartesian coordinates of : IMZW8_20

Atomic number (AN) and Cartesian coordinates

AN	X	Y	Z
6	-0.970539	2.620789	-0.135646
6	0.338606	2.307236	-0.367129
6	-0.968341	0.777979	-1.221901
1	-1.370052	3.477735	0.379021
1	1.256075	2.805479	-0.111987
1	1.141946	0.610670	-1.389437
1	-1.266982	-0.115650	-1.743240
7	-1.788405	1.658528	-0.679039
7	0.320738	1.133718	-1.068926
8	-2.123722	-1.448825	1.057112
1	-2.880347	-0.971049	0.656007
1	-2.517212	-2.033891	1.707613
8	-0.960693	-2.830995	-1.064484
1	-1.137412	-3.764914	-0.938673
1	-1.410011	-2.398345	-0.313845
8	1.712500	-2.443643	-0.293910
1	1.682873	-1.854932	0.480060
1	0.779721	-2.578625	-0.542101
8	1.908046	-0.561751	1.789595
1	1.039976	-0.174809	2.032721
1	2.245045	-0.942323	2.602713
8	2.693860	-0.309146	-1.821597
1	3.173893	0.161650	-1.117409
1	2.385527	-1.120563	-1.374905
8	3.718828	1.066951	0.427487
1	3.124882	0.534566	0.989038
1	4.595725	0.741637	0.638310
8	-4.072359	0.087076	-0.168460
1	-4.250269	-0.337417	-1.010094
1	-3.391326	0.760875	-0.381090
8	-0.573017	0.479892	2.363104
1	-0.605444	1.264103	1.804362
1	-1.148840	-0.162423	1.905970

Cartesian coordinates of : IMZW8_21

Atomic number (AN) and Cartesian coordinates

AN	X	Y	Z
6	1.605956	-2.312916	0.927875
6	2.415066	-1.262967	1.256535
6	1.687123	-1.046994	-0.793666
1	1.326497	-3.154607	1.538095
1	2.953917	-1.018810	2.153678
1	2.916312	0.442620	0.046784
1	1.545019	-0.618994	-1.771683
7	1.156189	-2.177114	-0.363726
7	2.462603	-0.470036	0.141743
8	-2.828861	-1.499258	0.846337
1	-2.440245	-1.862339	0.032479
1	-3.223762	-0.657524	0.563144
8	-1.168043	0.424240	-1.380056
1	-1.268215	-0.498393	-1.664436
1	-0.865731	0.329010	-0.460769
8	0.667270	2.644371	-1.034823
1	0.311709	1.845426	-1.443760
1	0.058459	2.789617	-0.290338
8	-3.563977	1.136338	-0.065332
1	-3.104971	1.742361	0.530068
1	-2.864995	0.929928	-0.709470
8	3.292104	2.228344	-0.252315
1	3.822342	2.293491	-1.048294
1	2.380472	2.417720	-0.552351
8	-1.415808	-2.389137	-1.447629
1	-1.561517	-3.124678	-2.044410
1	-0.498190	-2.494711	-1.111307
8	-1.306997	2.725705	1.021404
1	-1.187273	3.309425	1.772720
1	-1.025065	1.851168	1.339447
8	-0.633261	0.074720	1.468897
1	0.155495	-0.430412	1.692308
1	-1.350088	-0.585570	1.385642

Cartesian coordinates of : IMZW8_22

Atomic number (AN) and Cartesian coordinates

AN	X	Y	Z
6	-1.284390	-2.655553	0.052697
6	-2.353731	-1.922588	-0.377991
6	-1.078315	-0.708690	0.918217
1	-1.045323	-3.681161	-0.170225
1	-3.185844	-2.165318	-1.013181
1	-2.822592	0.125757	0.073744
1	-0.728161	0.142911	1.476673
7	-0.490068	-1.889606	0.871848
7	-2.209591	-0.686699	0.189325
8	2.775552	-0.967212	-0.984884

1	1.903834	-1.026654	-1.408324
1	2.617034	-1.235188	-0.063362
8	0.282287	1.734391	-1.367787
1	0.191918	0.807448	-1.640869
1	1.228192	1.830326	-1.167815
8	-1.217207	2.818656	0.687908
1	-0.672025	2.406277	-0.012528
1	-1.178198	3.756710	0.493742
8	3.011272	1.742711	-0.669384
1	3.757726	2.133555	-1.125467
1	3.060502	0.779927	-0.848711
8	-3.668118	1.761280	-0.033657
1	-4.252682	1.811661	0.724537
1	-2.857536	2.227959	0.254159
8	1.877601	1.267300	1.911387
1	2.331017	1.540222	1.101865
1	1.988436	0.303990	1.919772
8	0.232929	-0.962255	-2.263726
1	-0.422986	-1.563709	-1.891167
1	0.174955	-1.094789	-3.212108
8	2.132329	-1.554427	1.692852
1	1.208961	-1.827335	1.481734
1	2.485832	-2.241021	2.260117

Cartesian coordinates of : IMZW8_23

Atomic number (AN) and Cartesian coordinates

AN	X	Y	Z
6	0.615441	-2.680576	-0.432856
6	1.755795	-2.043617	-0.831796
6	1.103174	-1.284774	1.113283
1	0.066936	-3.450350	-0.948348
1	2.369411	-2.137680	-1.709224
1	2.815071	-0.476267	0.146240
1	1.098343	-0.696895	2.015345
7	0.214330	-2.207314	0.793235
7	2.059314	-1.164968	0.172975
8	-0.495699	0.313209	-1.493719
1	-0.005865	-0.517849	-1.483928
1	-0.634850	0.528769	-0.550174
8	-1.298905	1.113540	1.094081
1	-1.663691	0.250790	1.364106
1	-2.055598	1.564564	0.674597
8	1.194842	2.259168	1.543351
1	0.303360	1.873677	1.473708
1	1.042895	3.146004	1.874835
8	-3.498584	2.043141	-0.368434
1	-3.567860	1.138906	-0.720715
1	-4.255398	2.127857	0.214503
8	3.997122	0.889999	-0.330274
1	4.189296	1.361166	0.482941

1	3.256655	1.392547	-0.720619
8	1.687874	2.229282	-1.218943
1	1.490754	2.356923	-0.272167
1	1.027913	1.571626	-1.485954
8	-2.447144	-1.455932	1.426008
1	-1.563534	-1.844495	1.260588
1	-2.799261	-1.313709	0.533770
8	-3.148756	-0.615053	-1.217843
1	-2.246522	-0.389803	-1.506494
1	-3.556014	-1.044831	-1.972075

Cartesian coordinates of : IMZW8_24

Atomic number (AN) and Cartesian coordinates

AN	X	Y	Z
6	0.699812	2.733593	-0.337567
6	1.829627	2.130433	-0.810449
6	1.136771	1.124428	1.004081
1	0.175113	3.580001	-0.746444
1	2.454933	2.331237	-1.661123
1	2.829669	0.403191	-0.039172
1	1.109083	0.420492	1.818225
7	0.273767	2.104599	0.806880
7	2.100713	1.113243	0.065396
8	-2.298744	1.386273	1.565079
1	-1.415698	1.792390	1.405547
1	-2.670995	1.848275	2.317493
8	-3.031970	0.853059	-1.123730
1	-2.888609	1.119181	-0.200717
1	-3.348123	-0.063468	-1.046005
8	1.032499	-2.332129	1.860062
1	0.843987	-3.233249	2.128552
1	0.154107	-1.932222	1.727147
8	-1.330986	-1.168235	0.936329
1	-0.883164	-0.819089	0.149700
1	-1.712017	-0.369640	1.342129
8	1.338914	-2.270995	-0.958538
1	1.208975	-2.349353	0.003670
1	0.726892	-1.568653	-1.220342
8	-3.487965	-1.935491	-0.736319
1	-3.059740	-2.283668	-1.521182
1	-2.753805	-1.810308	-0.109219
8	-0.483515	-0.133024	-1.709543
1	-1.368173	0.262683	-1.568172
1	0.109527	0.625749	-1.713657
8	3.825669	-1.133238	-0.385849
1	3.018486	-1.576066	-0.711548
1	4.339713	-0.962862	-1.177132

Cartesian coordinates of : IMZW8_25

```
 ---------------------------------------------------
 Atomic number (AN) and Cartesian coordinates
  AN          X             Y             Z
 ---------------------------------------------------
  6       2.894601     -0.700275     -0.934201
  6       2.007086     -1.733066     -1.040357
  6       1.661823     -0.625373      0.810826
  1       3.673493     -0.418344     -1.621772
  1       1.866847     -2.490594     -1.789702
  1       0.468052     -2.313887      0.333034
  1       1.223725     -0.353600      1.756053
  7       2.676906     -0.009532      0.233734
  7       1.227961     -1.671507      0.082994
  8      -2.892026      2.188820     -0.332332
  1      -1.980786      2.390050     -0.066750
  1      -3.132876      2.909390     -0.917594
  8      -1.341898      0.440810      2.112604
  1      -2.018788      1.021191      2.466967
  1      -0.900063      0.986824      1.433907
  8      -2.784351     -1.448363      0.643354
  1      -2.313352     -0.791748      1.191277
  1      -2.659695     -1.103207     -0.256055
  8      -0.148293      2.145263      0.228475
  1       0.691439      2.395023      0.657751
  1       0.117430      1.729805     -0.613713
  8      -0.897073     -3.453060      0.735030
  1      -0.795871     -3.697624      1.656489
  1      -1.612539     -2.782576      0.738858
  8       2.369820      2.553803      1.393985
  1       2.824581      3.254856      0.923922
  1       2.663045      1.731309      0.949788
  8      -2.177644     -0.083491     -1.756327
  1      -2.499302      0.743983     -1.342282
  1      -2.739089     -0.208677     -2.523858
  8       0.358148      0.951914     -2.262833
  1       1.013259      0.261600     -2.111785
  1      -0.498116      0.490213     -2.194882
 ---------------------------------------------------

Cartesian coordinates of :  IMZW8_26

 ---------------------------------------------------
 Atomic number (AN) and Cartesian coordinates
  AN          X             Y             Z
 ---------------------------------------------------
  6      -3.795972     -0.698634     -0.315935
  6      -3.720336      0.659830     -0.219008
  6      -1.741980     -0.228579      0.047498
  1      -4.666237     -1.305859     -0.495947
  1      -4.463964      1.431857     -0.294462
  1      -1.976905      1.868688      0.104716
  1      -0.681608     -0.299375      0.217644
  7      -2.550555     -1.253469     -0.147099
```

```
  7     -2.402349      0.943513      0.015468
  8      1.224515     -1.927374      1.685680
  1      2.038641     -1.727362      1.195891
  1      0.645234     -2.325576      1.013678
  8      3.369405     -1.033510      0.018664
  1      4.051623     -1.672481     -0.196861
  1      2.704659     -1.123082     -0.696198
  8      1.035461      1.562736     -0.500383
  1      1.989081      1.709489     -0.644048
  1      0.976559      1.281789      0.434748
  8      1.242434     -0.997197     -1.746368
  1      1.007580     -0.137012     -1.355952
  1      0.641950     -1.637085     -1.329307
  8     -0.932023      3.432665      0.136361
  1     -1.208241      3.963047     -0.613136
  1     -0.160561      2.935953     -0.191561
  8      3.818144      1.594893     -0.871180
  1      3.825922      0.691775     -0.506361
  1      4.215930      2.131789     -0.183500
  8     -0.362520     -2.953227     -0.411481
  1     -0.455677     -3.870718     -0.671563
  1     -1.272495     -2.594831     -0.357906
  8      0.694441      0.769283      2.153347
  1      1.455748      1.059085      2.659610
  1      0.833963     -0.192258      2.039487
 ---------------------------------------------------

Cartesian coordinates of :  IMZW9_ 1

 ---------------------------------------------------
 Atomic number (AN) and Cartesian coordinates
  AN         X             Y             Z
 ---------------------------------------------------
  6      3.135175      1.367702     -0.396404
  6      3.436547      0.040660     -0.303140
  6      1.268066      0.320404     -0.404233
  1      3.811410      2.204632     -0.423744
  1      4.370652     -0.486162     -0.238276
  1      2.059597     -1.614601     -0.290458
  1      0.218039      0.073096     -0.419337
  7      1.774076      1.538273     -0.464077
  7      2.234654     -0.611544     -0.312837
  8     -2.203441     -2.005825      0.263380
  1     -2.652185     -1.144075      0.324902
  1     -1.732492     -1.968834     -0.584765
  8     -0.724611     -1.736751     -2.211512
  1     -1.059721     -0.816255     -2.211051
  1     -1.257421     -2.191094     -2.866478
  8      0.583026      0.561162      2.828519
  1      1.431971      0.760694      2.427660
  1      0.381246     -0.343876      2.522268
  8      0.977745     -3.218337     -0.412700
  1      0.627437     -2.982783      0.460729
  1      0.372856     -2.775295     -1.026736
```

```
  8     -0.504795      2.997681     -0.954988
  1      0.413290      2.665124     -0.823947
  1     -0.414984      3.904959     -1.250224
  8     -1.403283      2.113905      1.579064
  1     -1.047794      2.491958      0.757468
  1     -0.675752      1.581993      1.951882
  8     -1.876458      0.777035     -2.050117
  1     -1.343769      1.552223     -1.805857
  1     -2.467651      0.671478     -1.285857
  8     -3.289104      0.541761      0.387626
  1     -2.673758      1.112167      0.897312
  1     -4.171979      0.836475      0.614667
  8     -0.071794     -2.012830      1.965986
  1     -0.283848     -2.559140      2.725293
  1     -0.903267     -1.974798      1.443542
 ---------------------------------------------------

Cartesian coordinates of :  IMZW9_ 2

 ---------------------------------------------------
 Atomic number (AN) and Cartesian coordinates
  AN          X             Y             Z
 ---------------------------------------------------
  6     -1.481847     -2.591945     -0.967928
  6     -0.215063     -2.849772     -1.401780
  6     -0.338605     -0.817387     -0.598733
  1     -2.340343     -3.240898     -0.989594
  1      0.232555     -3.716512     -1.851905
  1      1.481808     -1.521444     -1.346764
  1     -0.023412      0.168146     -0.291707
  7     -1.556706     -1.314055     -0.467368
  7      0.496414     -1.705874     -1.164425
  8      0.325022     -1.865042      2.473460
  1     -0.409349     -1.221380      2.435095
  1      0.096822     -2.510680      1.800418
  8     -0.711254      2.354263      1.483388
  1      0.258055      2.269431      1.344568
  1     -0.815925      3.093740      2.084717
  8      3.079640     -0.442860     -1.269408
  1      2.575132      0.346373     -1.515974
  1      3.009049     -0.472567     -0.301819
  8     -1.846542     -0.090169      2.243759
  1     -2.026533     -0.486570      1.379113
  1     -1.452238      0.772947      2.018761
  8     -3.386027      0.862324     -0.768460
  1     -2.849857      0.057067     -0.627091
  1     -3.496833      1.218049      0.116262
  8      1.386335      1.775035     -1.979425
  1      0.445548      1.991906     -1.814657
  1      1.742857      2.548741     -2.419232
  8     -1.178816      2.569322     -1.296759
  1     -1.932412      1.953690     -1.307224
  1     -1.010481      2.657701     -0.343217
  8      1.921377      2.066941      0.828832
```

1	2.158792	1.164403	1.109059
1	1.809983	1.994109	-0.134005
8	2.558166	-0.566003	1.513205
1	1.773494	-1.038502	1.862560
1	3.225197	-0.644109	2.197103

Cartesian coordinates of : IMZW9_ 3

Atomic number (AN) and Cartesian coordinates

AN	X	Y	Z
6	3.410372	-0.760843	0.142955
6	3.437900	0.603292	0.084914
6	1.601324	-0.160204	-0.831040
1	4.147932	-1.424257	0.560361
1	4.156694	1.327412	0.421648
1	1.936019	1.898238	-0.740577
1	0.645147	-0.153196	-1.329398
7	2.260690	-1.234730	-0.440898
7	2.282609	0.966048	-0.549626
8	-1.792787	1.265497	-1.740963
1	-2.240835	1.408092	-0.890447
1	-1.688659	0.298671	-1.795041
8	-2.957886	1.531342	0.839232
1	-3.212471	0.588027	0.853096
1	-3.771477	2.022644	0.961364
8	0.249902	-3.096002	-0.766976
1	1.108441	-2.627441	-0.669254
1	0.456373	-4.031518	-0.773914
8	0.368150	2.911004	-1.339475
1	0.067663	3.798988	-1.537998
1	-0.380048	2.326030	-1.581593
8	1.008034	0.097194	2.478363
1	0.618908	0.876241	2.031188
1	1.871094	-0.013329	2.068075
8	-0.794751	-1.842604	1.559777
1	-0.409443	-2.307789	0.798984
1	-0.131099	-1.176710	1.819953
8	-0.295157	2.265306	1.370343
1	-0.022363	2.548754	0.482225
1	-1.224743	2.009180	1.255246
8	-3.247946	-1.176130	0.600875
1	-2.400028	-1.414568	1.035486
1	-3.915758	-1.705256	1.040157
8	-1.856321	-1.556861	-1.852959
1	-2.455293	-1.555518	-1.090007
1	-1.106621	-2.095357	-1.552592

Cartesian coordinates of : IMZW9_ 4

Atomic number (AN) and Cartesian coordinates

AN	X	Y	Z
6	-3.410423	-0.760783	0.142826
6	-3.437939	0.603351	0.084774
6	-1.601265	-0.160155	-0.830974
1	-4.148029	-1.424192	0.560156
1	-4.156764	1.327473	0.421436
1	-1.935957	1.898289	-0.740552
1	-0.645032	-0.153160	-1.329229
7	-2.260678	-1.234678	-0.440899
7	-2.282575	0.966100	-0.549638
8	1.792797	1.265529	-1.740999
1	2.240836	1.408126	-0.890478
1	1.688688	0.298703	-1.795080
8	1.856378	-1.556865	-1.852954
1	1.106682	-2.095363	-1.552587
1	2.455333	-1.555481	-1.089988
8	0.295160	2.265124	1.370494
1	1.224758	2.009049	1.255372
1	0.022376	2.548687	0.482411
8	-0.368095	2.911093	-1.339283
1	0.380068	2.326110	-1.581477
1	-0.067568	3.799082	-1.537720
8	-0.249927	-3.095959	-0.767058
1	-1.108470	-2.627410	-0.669298
1	-0.456368	-4.031484	-0.773764
8	-1.008181	0.097011	2.478349
1	-1.871116	-0.013658	2.067838
1	-0.619016	0.876045	2.031190
8	0.794760	-1.842666	1.559729
1	0.409470	-2.307806	0.798896
1	0.131080	-1.176816	1.819951
8	3.247950	-1.176092	0.600911
1	2.400030	-1.414556	1.035508
1	3.915753	-1.705287	1.040124
8	2.957886	1.531381	0.839186
1	3.212474	0.588069	0.853095
1	3.771483	2.022703	0.961191

Cartesian coordinates of : IMZW9_ 5

Atomic number (AN) and Cartesian coordinates

AN	X	Y	Z
6	3.267883	1.054360	-0.422243
6	3.404171	-0.302592	-0.443686
6	1.285923	0.245546	-0.400729
1	4.041866	1.802303	-0.427407
1	4.266978	-0.942385	-0.470068
1	1.835868	-1.777123	-0.461008
1	0.213780	0.127943	-0.370946

7	1.937357	1.393971	-0.398942
7	2.130971	-0.801442	-0.434262
8	0.729897	0.218697	2.816367
1	1.582141	0.351747	2.395467
1	0.145949	0.886871	2.401416
8	-0.318214	-2.141447	1.846165
1	-0.527371	-2.660004	2.624776
1	0.077040	-1.312546	2.189768
8	-0.165071	3.139743	-0.680628
1	0.011987	4.035481	-0.971737
1	0.713588	2.698823	-0.614999
8	0.647652	-3.274804	-0.552084
1	0.292541	-2.987816	0.305679
1	0.095870	-2.800731	-1.193680
8	-1.004344	-1.499003	-2.144539
1	-1.445186	-1.879472	-2.906795
1	-1.626477	-1.644894	-1.398427
8	-1.828394	1.264928	-1.985046
1	-1.152795	1.871424	-1.636883
1	-1.389974	0.404259	-2.082312
8	-2.509602	-1.856332	0.124266
1	-2.855482	-0.959187	0.271579
1	-1.780644	-1.935217	0.765251
8	-3.166873	0.844762	0.383875
1	-2.782447	1.047870	-0.495755
1	-4.054557	1.206207	0.363217
8	-1.052845	2.071734	1.780878
1	-1.840656	1.632629	1.417431
1	-0.705833	2.555997	1.013784

Cartesian coordinates of : IMZW9_ 6

Atomic number (AN) and Cartesian coordinates

AN	X	Y	Z
0	0.000000	0.000000	0.000000
0	0.000000	0.000000	0.000000
0	0.000000	0.000000	0.000000
0	0.000000	0.000000	0.000000
0	0.000000	0.000000	0.000000
0	0.000000	0.000000	0.000000
0	0.000000	0.000000	0.000000
0	0.000000	0.000000	0.000000
0	0.000000	0.000000	0.000000
0	0.000000	0.000000	0.000000
0	0.000000	0.000000	0.000000
0	0.000000	0.000000	0.000000
0	0.000000	0.000000	0.000000
0	0.000000	0.000000	0.000000
0	0.000000	0.000000	0.000000
0	0.000000	0.000000	0.000000
0	0.000000	0.000000	0.000000

```
  0      0.000000      0.000000      0.000000
  0      0.000000      0.000000      0.000000
  0      0.000000      0.000000      0.000000
  0      0.000000      0.000000      0.000000
  0      0.000000      0.000000      0.000000
  0      0.000000      0.000000      0.000000
  0      0.000000      0.000000      0.000000
  0      0.000000      0.000000      0.000000
  0      0.000000      0.000000      0.000000
  0      0.000000      0.000000      0.000000
  0      0.000000      0.000000      0.000000
  0      0.000000      0.000000      0.000000
  0      0.000000      0.000000      0.000000
  0      0.000000      0.000000      0.000000
  0      0.000000      0.000000      0.000000
  0      0.000000      0.000000      0.000000
  0      0.000000      0.000000      0.000000
  0      0.000000      0.000000      0.000000
  0      0.000000      0.000000      0.000000
 ---------------------------------------------------

Cartesian coordinates of :  IMZW9_ 7

 ---------------------------------------------------
 Atomic number (AN) and Cartesian coordinates
  AN          X             Y             Z
 ---------------------------------------------------
  6      3.302447      1.135902     -0.311251
  6      3.476290     -0.216404     -0.336462
  6      1.343542      0.272646     -0.294373
  1      4.055120      1.905243     -0.312585
  1      4.356337     -0.832139     -0.363251
  1      1.948493     -1.732891     -0.365536
  1      0.275056      0.125045     -0.270214
  7      1.962819      1.438182     -0.285186
  7      2.217149     -0.750265     -0.325517
  8     -0.396924     -2.200666      1.812371
  1     -0.088965     -1.356217      2.205410
  1     -0.640789     -2.751822      2.557645
  8     -2.452666     -1.919067     -0.063531
  1     -2.837584     -1.035929      0.070841
  1     -1.779874     -1.994884      0.637018
  8     -0.151880      3.153636     -0.626791
  1      0.727941      2.720986     -0.523569
  1      0.027279      4.058834     -0.885118
  8      0.787206     -3.249582     -0.527266
  1      0.363319     -2.977518      0.303811
  1      0.274747     -2.781676     -1.204642
  8     -1.698189      1.261651     -2.044912
  1     -1.067910      1.881949     -1.640494
  1     -1.228990      0.415093     -2.118590
  8     -1.272280      2.047486      1.708153
  1     -0.861689      2.527740      0.969643
  1     -2.023484      1.597295      1.286221
```

8	-0.787188	-1.476949	-2.202044
1	-1.177472	-1.843838	-2.997835
1	-1.450792	-1.650276	-1.498608
8	-3.241738	0.751606	0.169611
1	-2.788193	0.984317	-0.669107
1	-4.139316	1.073924	0.071915
8	0.459945	0.207878	2.834240
1	-0.146368	0.862198	2.428702
1	0.239390	0.228346	3.767147

Cartesian coordinates of : IMZW9_ 8

Atomic number (AN) and Cartesian coordinates

AN	X	Y	Z
6	-3.302452	1.135890	-0.311247
6	-3.476284	-0.216417	-0.336456
6	-1.343540	0.272650	-0.294369
1	-4.055130	1.905226	-0.312590
1	-4.356326	-0.832160	-0.363249
1	-1.948470	-1.732890	-0.365522
1	-0.275054	0.125054	-0.270197
7	-1.962826	1.438182	-0.285184
7	-2.217139	-0.750267	-0.325513
8	0.396936	-2.200648	1.812392
1	0.088976	-1.356196	2.205422
1	0.640805	-2.751790	2.557677
8	-0.459926	0.207910	2.834245
1	0.146384	0.862226	2.428696
1	-0.239354	0.228381	3.767147
8	0.787164	-1.476977	-2.202044
1	1.450778	-1.650303	-1.498616
1	1.177437	-1.843866	-2.997840
8	-0.787203	-3.249587	-0.527221
1	-0.363300	-2.977513	0.303845
1	-0.274748	-2.781700	-1.204614
8	0.151870	3.153643	-0.626795
1	-0.027291	4.058837	-0.885130
1	-0.727949	2.720992	-0.523566
8	1.698155	1.261631	-2.044926
1	1.228966	0.415068	-2.118602
1	1.067875	1.881924	-1.640504
8	1.272309	2.047504	1.708132
1	0.861711	2.527753	0.969622
1	2.023506	1.597306	1.286196
8	3.241743	0.751601	0.169574
1	4.139320	1.073918	0.071870
1	2.788190	0.984312	-0.669140
8	2.452654	-1.919067	-0.063539
1	2.837578	-1.035928	0.070819
1	1.779876	-1.994877	0.637024

Cartesian coordinates of : IMZW9_ 9

Atomic number (AN) and Cartesian coordinates

AN	X	Y	Z
6	3.358600	-1.437782	0.561999
6	3.538954	-0.127282	0.901277
6	2.436657	-0.283628	-0.980277
1	3.686733	-2.312690	1.096654
1	4.023639	0.344395	1.736470
1	2.810552	1.605911	-0.136676
1	1.906224	0.029359	-1.864106
7	2.672497	-1.533930	-0.626062
7	2.955649	0.596060	-0.103215
8	-2.769738	-1.843277	0.513952
1	-2.627631	-1.223715	1.250228
1	-1.879473	-2.089592	0.231441
8	-0.162160	-2.306703	-0.728739
1	-0.416541	-1.593352	-1.349382
1	0.808616	-2.306662	-0.768173
8	-0.956476	-0.157165	-2.328672
1	-0.671194	0.504774	-1.672358
1	-1.919912	-0.173560	-2.213834
8	0.258324	-0.551298	1.513268
1	0.103386	-1.218021	0.820383
1	1.184072	-0.657889	1.756101
8	-3.629676	-0.087187	-1.416849
1	-4.489697	-0.317523	-1.771886
1	-3.420669	-0.786008	-0.762265
8	-0.281803	1.613641	-0.233522
1	-0.026903	0.922252	0.403225
1	-1.204775	1.829645	0.006208
8	1.933597	3.290541	-0.252305
1	1.079703	2.818699	-0.266575
1	2.045062	3.601815	-1.152262
8	-2.974504	1.936541	0.448887
1	-3.316756	1.310299	-0.211265
1	-2.845438	1.387168	1.237465
8	-2.232812	0.117398	2.489237
1	-2.424916	-0.052301	3.412877
1	-1.284398	-0.065676	2.382616

Cartesian coordinates of : IMZW9_10

Atomic number (AN) and Cartesian coordinates

AN	X	Y	Z
0	0.000000	0.000000	0.000000
0	0.000000	0.000000	0.000000
0	0.000000	0.000000	0.000000

```
  0      0.000000      0.000000      0.000000
  0      0.000000      0.000000      0.000000
  0      0.000000      0.000000      0.000000
  0      0.000000      0.000000      0.000000
  0      0.000000      0.000000      0.000000
  0      0.000000      0.000000      0.000000
  0      0.000000      0.000000      0.000000
  0      0.000000      0.000000      0.000000
  0      0.000000      0.000000      0.000000
  0      0.000000      0.000000      0.000000
  0      0.000000      0.000000      0.000000
  0      0.000000      0.000000      0.000000
  0      0.000000      0.000000      0.000000
  0      0.000000      0.000000      0.000000
  0      0.000000      0.000000      0.000000
  0      0.000000      0.000000      0.000000
  0      0.000000      0.000000      0.000000
  0      0.000000      0.000000      0.000000
  0      0.000000      0.000000      0.000000
  0      0.000000      0.000000      0.000000
  0      0.000000      0.000000      0.000000
  0      0.000000      0.000000      0.000000
  0      0.000000      0.000000      0.000000
  0      0.000000      0.000000      0.000000
  0      0.000000      0.000000      0.000000
  0      0.000000      0.000000      0.000000
  0      0.000000      0.000000      0.000000
  0      0.000000      0.000000      0.000000
  0      0.000000      0.000000      0.000000
  0      0.000000      0.000000      0.000000
  0      0.000000      0.000000      0.000000
  0      0.000000      0.000000      0.000000
  0      0.000000      0.000000      0.000000
 ---------------------------------------------------

Cartesian coordinates of :  IMZW9_11

 ---------------------------------------------------
 Atomic number (AN) and Cartesian coordinates
  AN          X             Y             Z
 ---------------------------------------------------
  6      0.679000      2.827581     -0.491382
  6      1.829939      2.101134     -0.370641
  6      0.337343      1.396319      1.062872
  1      0.470379      3.638781     -1.167420
  1      2.771979      2.147421     -0.886866
  1      2.253805      0.483429      0.950617
  1     -0.102177      0.825851      1.862542
  7     -0.253386      2.384119      0.415696
  7      1.597279      1.200594      0.631852
  8      3.578258     -0.813054      1.216104
  1      3.554980     -1.030927      0.264759
  1      4.403123     -0.337368      1.329605
  8     -2.295501     -2.438300     -0.725437
```

1	-1.324289	-2.477551	-0.797723
1	-2.608402	-3.328986	-0.889675
8	0.468993	-1.974989	-0.755592
1	0.489201	-1.801721	0.205233
1	0.231075	-1.124410	-1.177715
8	-2.873315	0.205499	-1.556842
1	-2.967727	0.689035	-0.718216
1	-2.819244	-0.726648	-1.295320
8	-2.891267	1.619668	0.878077
1	-2.052976	2.102455	0.714790
1	-3.538385	2.282931	1.121700
8	3.122016	-1.331687	-1.492315
1	2.224852	-1.646353	-1.276372
1	2.973628	-0.438898	-1.813538
8	0.363600	-1.697833	2.041066
1	0.366299	-2.608473	2.341939
1	-0.582972	-1.453811	2.016995
8	-0.196037	0.225083	-2.295188
1	0.234293	1.021501	-1.965662
1	-1.135096	0.331418	-2.040982
8	-2.323874	-1.036717	1.727232
1	-2.428569	-1.534432	0.899508
1	-2.521701	-0.117814	1.487045

Cartesian coordinates of : IMZW9_12

Atomic number (AN) and Cartesian coordinates

AN	X	Y	Z
6	-0.044998	2.728247	1.101800
6	-1.379315	2.611576	0.836895
6	-0.441762	0.635659	0.871001
1	0.524828	3.623638	1.281741
1	-2.162606	3.341174	0.742719
1	-2.514562	0.815469	0.523901
1	-0.344915	-0.437605	0.809323
7	0.536794	1.484011	1.129120
7	-1.615191	1.272702	0.697416
8	-2.192438	-2.123139	-0.702518
1	-1.432888	-2.378919	-0.148359
1	-1.808806	-1.497855	-1.339534
8	0.181448	-2.946999	0.586798
1	0.697867	-2.520530	-0.117389
1	0.494858	-2.522018	1.405224
8	0.648681	2.088896	-2.142770
1	0.050271	1.325299	-2.234875
1	0.350682	2.524939	-1.338352
8	-3.981359	-0.289632	0.304553
1	-3.395079	-1.026069	0.035771
1	-4.261850	-0.519247	1.192072
8	2.827864	-0.026786	1.272855
1	3.535507	0.358043	1.793740

```
  1      2.099776      0.642604      1.295719
  8      2.927960      0.525263     -1.519921
  1      2.176797      1.132440     -1.653073
  1      3.008837      0.438319     -0.555889
  8      1.196189     -1.677059      2.896159
  1      1.817677     -1.118890      2.394201
  1      0.501759     -1.066656      3.153500
  8      1.353912     -1.722363     -1.665855
  1      1.997410     -0.981080     -1.652255
  1      1.716004     -2.357908     -2.286232
  8     -0.857771     -0.287408     -2.439733
  1     -1.079472     -0.410019     -3.364971
  1     -0.062752     -0.841212     -2.297452
 ---------------------------------------------------

Cartesian coordinates of :  IMZW9_13

 ---------------------------------------------------
 Atomic number (AN) and Cartesian coordinates
  AN          X             Y             Z
 ---------------------------------------------------
  6     -0.906591     -2.772874     -1.038015
  6     -2.149830     -2.290650     -0.753609
  6     -0.671157     -0.686349     -0.611530
  1     -0.622997     -3.777870     -1.299325
  1     -3.116299     -2.759213     -0.722554
  1     -2.714940     -0.281396     -0.260096
  1     -0.257270      0.295181     -0.445916
  7      0.019441     -1.760257     -0.955551
  7     -1.981979     -0.958519     -0.488587
  8     -1.608127      2.675242     -0.087988
  1     -1.227514      2.183699      0.658436
  1     -0.987613      2.508615     -0.813233
  8      2.252579     -0.205986     -2.064278
  1      1.719922      0.606851     -2.106667
  1      1.616571     -0.876720     -1.763976
  8     -0.139894     -1.432079      2.601427
  1     -0.814215     -1.921478      2.124827
  1      0.681360     -1.628015      2.107864
  8     -3.886591      1.115824      0.072130
  1     -4.450159      1.185828     -0.700268
  1     -3.164638      1.752837     -0.093601
  8      2.100958     -2.045459      1.053878
  1      1.537047     -2.012146      0.261966
  1      2.703230     -1.293274      0.930994
  8     -0.527682      1.257534      2.116935
  1     -0.425662      0.297448      2.286506
  1     -0.594383      1.659387      2.984770
  8      0.675666      2.166545     -1.829815
  1      0.964236      2.931319     -2.331993
  1      1.086368      2.280001     -0.944386
  8      3.567042      0.240391      0.308098
  1      4.516559      0.329781      0.211440
  1      3.222993      0.093111     -0.598479
```

8	1.696409	2.285773	0.718532
1	2.399598	1.615317	0.681993
1	0.988164	1.879110	1.248221

Cartesian coordinates of : IMZW9_14

Atomic number (AN) and Cartesian coordinates

AN	X	Y	Z
6	0.115262	2.633715	-0.845877
6	1.378561	2.113884	-0.855580
6	0.383914	1.655806	1.037811
1	-0.379348	3.196863	-1.618548
1	2.161980	2.134901	-1.591383
1	2.341012	0.965773	0.684376
1	0.238593	1.259429	2.028786
7	-0.503079	2.348976	0.348530
7	1.535306	1.503178	0.358839
8	-0.699940	-0.507457	-1.516188
1	-0.864438	-0.605369	-0.557918
1	-0.274754	0.354146	-1.600115
8	-2.946786	1.330987	1.209246
1	-3.420546	1.858583	1.853897
1	-2.177820	1.875355	0.929344
8	-1.475101	-1.067899	1.150533
1	-2.150791	-1.660980	0.771542
1	-1.964038	-0.254158	1.358880
8	3.526218	-0.338575	1.386740
1	3.784165	-0.528276	0.467970
1	2.766807	-0.921750	1.539641
8	-3.505718	-2.419613	-0.231628
1	-3.613532	-1.563533	-0.688101
1	-3.050725	-2.967990	-0.873852
8	1.133434	-1.850938	1.702802
1	0.208387	-1.564710	1.593326
1	1.084710	-2.662739	2.210287
8	1.576106	-2.168881	-1.086944
1	1.489730	-2.158986	-0.117992
1	0.818238	-1.633990	-1.376040
8	3.888228	-0.686147	-1.407971
1	3.045342	-1.183866	-1.376820
1	3.624475	0.202000	-1.656667
8	-3.511851	0.182643	-1.322315
1	-3.454391	0.630506	-0.462168
1	-2.581782	0.050326	-1.562355

Cartesian coordinates of : IMZW9_15

Atomic number (AN) and Cartesian coordinates

AN X Y Z

6	0.710032	2.865315	-1.003827
6	1.922008	2.410186	-0.576124
6	0.435347	0.808063	-0.473608
1	0.451400	3.849987	-1.353159
1	2.881157	2.885728	-0.483322
1	2.447747	0.452124	0.094739
1	0.011543	-0.166202	-0.288680
7	-0.218333	1.854956	-0.942128
7	1.730842	1.096162	-0.245775
8	1.678021	-2.470086	-0.027500
1	1.303164	-2.138592	-0.864335
1	1.201730	-1.994127	0.674161
8	0.657628	-1.638876	-2.528413
1	0.710158	-2.430289	-3.067408
1	-0.290592	-1.572607	-2.293518
8	-0.278867	1.464122	2.571733
1	-0.361135	1.757563	3.481549
1	0.155508	2.192215	2.121326
8	3.781920	-0.782034	0.584600
1	4.427545	-0.739899	-0.123228
1	3.159237	-1.481224	0.302922
8	-2.848525	1.111499	-1.240195
1	-3.419674	1.717583	-1.714153
1	-1.953950	1.531112	-1.231080
8	-2.719559	0.463341	1.492802
1	-1.895698	0.874272	1.802288
1	-2.831359	0.781281	0.580522
8	-2.008262	-1.524836	-1.770894
1	-2.332676	-0.611214	-1.700002
1	-1.995501	-1.829879	-0.847581
8	-1.931317	-2.110122	0.998560
1	-2.310884	-1.229931	1.214044
1	-2.548475	-2.745677	1.365537
8	0.455995	-1.296849	2.256015
1	-0.397511	-1.648579	1.951863
1	0.300108	-0.342124	2.320529

Cartesian coordinates of : IMZW9_16

Atomic number (AN) and Cartesian coordinates

AN	X	Y	Z
6	-3.107012	-0.864768	1.063422
6	-2.096055	-1.782390	1.105496
6	-2.091701	-0.734313	-0.812476
1	-3.834752	-0.642217	1.824806
1	-1.782371	-2.478667	1.861710
1	-0.636465	-2.206777	-0.415860
1	-1.789431	-0.458758	-1.808583
7	-3.103607	-0.210294	-0.146600
7	-1.461840	-1.689418	-0.103219

8	0.999424	-0.224701	-2.381148
1	1.883901	-0.107042	-1.971217
1	1.123274	-0.008338	-3.307253
8	2.348802	-1.791262	1.073847
1	3.064445	-2.231981	1.534394
1	2.159557	-0.984529	1.601752
8	-0.765627	1.267320	1.876037
1	-0.542683	1.377693	0.932770
1	-1.339606	0.493603	1.891759
8	-2.735134	2.460986	-1.138261
1	-3.026694	1.622867	-0.730473
1	-2.911800	3.123326	-0.467436
8	0.973641	-2.801030	-1.172658
1	0.994042	-2.028542	-1.759703
1	1.498257	-2.510718	-0.409108
8	1.882788	0.556773	2.384750
1	0.924260	0.726396	2.321595
1	2.248725	1.210739	1.769616
8	3.321470	-0.003807	-0.938455
1	3.137848	0.848653	-0.508213
1	3.079096	-0.663127	-0.266320
8	2.471381	2.388304	0.299194
1	1.582129	2.427250	-0.091869
1	2.846331	3.262153	0.180294
8	-0.024606	1.844242	-0.775303
1	-0.934775	2.112960	-1.004148
1	0.206345	1.127268	-1.392459

Cartesian coordinates of : IMZW9_17

Atomic number (AN) and Cartesian coordinates

AN	X	Y	Z
6	-1.931684	-2.477916	-0.002365
6	-2.891245	-1.650011	0.505395
6	-1.663438	-0.578274	-0.952639
1	-1.749945	-3.512614	0.232568
1	-3.670166	-1.805841	1.229075
1	-3.242492	0.415494	0.040121
1	-1.308409	0.227049	-1.573411
7	-1.168127	-1.801708	-0.923308
7	-2.709560	-0.445277	-0.116478
8	1.648186	0.852893	-1.654025
1	2.570935	1.028850	-1.394209
1	1.616748	-0.110030	-1.779524
8	-3.963971	2.090471	0.288168
1	-3.143938	2.522513	-0.025723
1	-4.592831	2.230395	-0.421761
8	2.299627	-1.730192	0.871537
1	2.713082	-2.533183	1.192715
1	2.100972	-1.899315	-0.069967
8	1.457506	-1.945912	-1.738466

1	1.658786	-2.605003	-2.403849
1	0.492142	-2.030963	-1.544558
8	4.253765	1.324744	-0.685288
1	3.910839	1.246171	0.226168
1	4.449164	2.257851	-0.786861
8	3.015058	0.845332	1.769896
1	2.852261	-0.076772	1.508594
1	2.172479	1.272370	1.550402
8	-1.523804	3.065442	-0.567171
1	-1.393865	3.956722	-0.238606
1	-0.885199	2.527241	-0.059160
8	-0.016338	-0.843051	2.184490
1	0.757722	-1.250115	1.753812
1	-0.759053	-1.372520	1.875840
8	0.403976	1.532941	0.728869
1	0.806906	1.214313	-0.105708
1	0.113827	0.722158	1.186109

Cartesian coordinates of : IMZW9_18

Atomic number (AN) and Cartesian coordinates

AN	X	Y	Z
6	0.176754	2.781624	-0.484082
6	-1.032014	2.237244	-0.813740
6	0.553234	0.746703	-1.032972
1	0.390349	3.785387	-0.159259
1	-2.026323	2.644711	-0.834516
1	-1.454320	0.241515	-1.467627
1	1.025530	-0.196548	-1.251447
7	1.170104	1.842601	-0.629349
7	-0.770695	0.944345	-1.171983
8	-2.302478	-0.596210	1.694332
1	-1.605994	0.044814	1.956302
1	-2.644201	-0.942097	2.520892
8	-0.279250	1.123537	2.358510
1	0.486447	0.541277	2.170740
1	-0.274706	1.765357	1.640693
8	-2.653818	-1.118660	-1.910649
1	-2.172048	-1.716558	-1.309653
1	-3.317763	-0.699583	-1.335346
8	-4.241794	0.333059	-0.072802
1	-3.623421	0.051992	0.627651
1	-5.059418	-0.121032	0.138053
8	3.592501	0.919159	0.334160
1	4.309427	1.545865	0.442718
1	2.828553	1.437231	-0.010193
8	3.499796	-1.193978	-1.482572
1	3.070431	-0.831174	-2.260039
1	3.608480	-0.424446	-0.892317
8	1.860761	-0.583861	1.994323
1	2.539129	-0.041419	1.561468

1	1.709442	-1.302330	1.355017
8	1.592451	-2.554292	-0.010313
1	1.984914	-3.390393	0.246592
1	2.255103	-2.128919	-0.592731
8	-1.225638	-2.572382	0.019223
1	-0.259260	-2.500552	-0.032284
1	-1.494298	-1.916115	0.685684

Cartesian coordinates of : IMZW9_19

Atomic number (AN) and Cartesian coordinates

AN	X	Y	Z
6	-3.392760	-0.536231	-0.689405
6	-2.434716	-1.346157	-1.227664
6	-2.052734	-1.027326	0.901744
1	-4.235952	-0.083719	-1.182861
1	-2.282757	-1.727659	-2.220949
1	-0.731248	-2.207029	-0.254778
1	-1.565072	-1.101252	1.858704
7	-3.154251	-0.343503	0.651693
7	-1.592456	-1.657673	-0.194779
8	-1.087309	1.779391	-1.032507
1	-1.716665	2.134171	-0.375066
1	-1.534995	1.005676	-1.391289
8	1.613958	2.088274	-1.761758
1	2.127721	2.192729	-0.945563
1	0.688691	2.113240	-1.472234
8	1.427675	-1.428162	2.024987
1	1.396354	-1.523512	2.977974
1	0.998063	-0.570574	1.829200
8	-2.872965	2.481119	1.014562
1	-2.263224	2.638757	1.738575
1	-3.066991	1.525650	1.073786
8	0.992186	-2.922016	-0.315004
1	1.183310	-2.566334	0.567483
1	1.296926	-2.214700	-0.902801
8	0.471433	1.058810	1.316114
1	-0.020484	1.161285	0.486230
1	1.337918	1.462424	1.137048
8	1.777227	-0.670719	-1.899879
1	2.201815	-0.882068	-2.733260
1	1.712330	0.307877	-1.892894
8	3.595268	-0.771368	0.300841
1	3.056505	-0.797756	-0.508716
1	2.976637	-1.072615	0.985712
8	3.063906	1.918243	0.646509
1	3.693124	2.400379	1.184603
1	3.405182	1.002967	0.583729

Cartesian coordinates of : IMZW9_20

Atomic number (AN) and Cartesian coordinates

AN	X	Y	Z
6	-0.639297	-2.881409	0.579365
6	-1.812130	-2.620832	-0.070192
6	-1.088957	-0.819019	0.935474
1	-0.098538	-3.810600	0.634881
1	-2.462207	-3.239359	-0.661349
1	-2.890144	-0.760588	-0.157242
1	-1.064098	0.201931	1.278515
7	-0.192892	-1.746937	1.214465
7	-2.085988	-1.303316	0.172192
8	-0.857327	3.003491	1.597081
1	0.008422	2.603824	1.404590
1	-0.687417	3.946983	1.586571
8	-2.133666	2.348885	-0.823058
1	-1.387964	1.851170	-1.199761
1	-1.782772	2.619522	0.045159
8	2.250241	-0.778405	2.045878
1	1.384940	-1.221560	1.869375
1	2.504488	-1.033131	2.933758
8	-4.147055	0.488589	-0.647737
1	-4.665301	0.670795	0.137996
1	-3.477905	1.205302	-0.669334
8	0.836422	-1.592826	-1.981071
1	0.838511	-1.937723	-2.876497
1	0.246606	-2.180073	-1.493098
8	3.260583	-1.016768	-0.592333
1	3.008509	-0.994408	0.344598
1	2.448893	-1.285168	-1.052087
8	0.311745	1.129447	-1.493622
1	0.588923	1.205371	-0.566192
1	0.421815	0.184255	-1.696581
8	3.208416	1.691938	-1.331079
1	2.320395	1.702519	-1.711068
1	3.317572	0.751297	-1.091753
8	1.584656	1.729390	1.060781
1	2.274268	1.877395	0.394466
1	1.835355	0.891664	1.491839

Cartesian coordinates of : IMZW9_21

Atomic number (AN) and Cartesian coordinates

AN	X	Y	Z
0	0.000000	0.000000	0.000000
0	0.000000	0.000000	0.000000
0	0.000000	0.000000	0.000000
0	0.000000	0.000000	0.000000
0	0.000000	0.000000	0.000000

```
  0      0.000000      0.000000      0.000000
  0      0.000000      0.000000      0.000000
  0      0.000000      0.000000      0.000000
  0      0.000000      0.000000      0.000000
  0      0.000000      0.000000      0.000000
  0      0.000000      0.000000      0.000000
  0      0.000000      0.000000      0.000000
  0      0.000000      0.000000      0.000000
  0      0.000000      0.000000      0.000000
  0      0.000000      0.000000      0.000000
  0      0.000000      0.000000      0.000000
  0      0.000000      0.000000      0.000000
  0      0.000000      0.000000      0.000000
  0      0.000000      0.000000      0.000000
  0      0.000000      0.000000      0.000000
  0      0.000000      0.000000      0.000000
  0      0.000000      0.000000      0.000000
  0      0.000000      0.000000      0.000000
  0      0.000000      0.000000      0.000000
  0      0.000000      0.000000      0.000000
  0      0.000000      0.000000      0.000000
  0      0.000000      0.000000      0.000000
  0      0.000000      0.000000      0.000000
  0      0.000000      0.000000      0.000000
  0      0.000000      0.000000      0.000000
  0      0.000000      0.000000      0.000000
  0      0.000000      0.000000      0.000000
  0      0.000000      0.000000      0.000000
  0      0.000000      0.000000      0.000000
  0      0.000000      0.000000      0.000000
  0      0.000000      0.000000      0.000000
 ---------------------------------------------------

Cartesian coordinates of :  IMZW9_22

 ---------------------------------------------------
 Atomic number (AN) and Cartesian coordinates
  AN         X            Y            Z
 ---------------------------------------------------
  6      3.498635     -0.050059     -0.281497
  6      3.213005      1.277107     -0.416684
  6      1.368696      0.100571     -0.429160
  1      4.462899     -0.517787     -0.181564
  1      3.837749      2.150326     -0.457362
  1      1.266597      2.180881     -0.639001
  1      0.312583     -0.119938     -0.465817
  7      2.338658     -0.785187     -0.293351
  7      1.851363      1.353445     -0.513875
  8     -3.134514      1.034943      0.205122
  1     -4.061240      1.257440      0.308076
  1     -3.087393      0.063343      0.311501
  8     -0.327267      3.159963     -0.863612
  1     -0.586806      2.922748      0.046330
  1     -0.722755      2.448483     -1.395783
```

```
  8      0.901281     -3.121752     -0.486546
  1      1.376113     -3.950660     -0.563701
  1      1.592655     -2.422542     -0.425273
  8     -1.639057      0.997719     -2.200341
  1     -1.285711      0.093404     -2.180160
  1     -2.264005      1.013543     -1.456264
  8      0.638080      0.246198      2.707855
  1      1.563837      0.390499      2.495904
  1      0.606307      0.275008      3.666830
  8     -0.474032     -2.207978      1.804572
  1     -0.026730     -1.387106      2.069294
  1      0.067703     -2.571104      1.082969
  8     -1.101452      2.258690      1.685457
  1     -0.498334      1.564325      1.996193
  1     -1.848835      1.780445      1.286760
  8     -2.663645     -1.647793      0.275062
  1     -1.923590     -1.846070      0.890716
  1     -3.357177     -2.269156      0.502909
  8     -1.095899     -1.830701     -2.062089
  1     -1.736517     -1.838110     -1.330651
  1     -0.327473     -2.275787     -1.671122
 ---------------------------------------------------

Cartesian coordinates of :  IMZW9_23

 ---------------------------------------------------
 Atomic number (AN) and Cartesian coordinates
  AN          X             Y             Z
 ---------------------------------------------------
  6      0.013733      2.999333     -0.582124
  6     -1.244612      2.973512     -0.057964
  6     -0.568592      0.938791     -0.493284
  1      0.639692      3.852953     -0.777471
  1     -1.904183      3.753341      0.275236
  1     -2.477227      1.242296      0.311098
  1     -0.584163     -0.136760     -0.559723
  7      0.433000      1.718963     -0.860756
  7     -1.599824      1.653260     -0.012149
  8      2.700227      1.329003      0.968078
  1      3.250064      0.601454      0.639475
  1      2.079549      1.485568      0.238632
  8      0.668542      0.162502      2.473742
  1      1.408189      0.621583      2.026417
  1      0.999164     -0.038453      3.351180
  8     -2.896529     -1.261799     -1.758753
  1     -3.472562     -1.983000     -2.018723
  1     -2.102288     -1.704408     -1.418461
  8     -3.758469     -0.116028      0.677519
  1     -3.086150     -0.580485      1.207615
  1     -3.616796     -0.505850     -0.203502
  8     -1.579680     -1.520307      1.765756
  1     -1.342144     -1.891647      0.902503
  1     -0.856861     -0.904820      1.958463
  8     -0.560416     -2.581095     -0.799727
```

1	-0.687715	-3.529484	-0.738531
1	0.266738	-2.406848	-0.302084
8	3.836085	-0.899107	-0.361072
1	4.642492	-1.310092	-0.675536
1	3.313390	-0.684534	-1.159921
8	1.576290	-2.067987	0.830035
1	1.279774	-1.358451	1.420724
1	2.406302	-1.735600	0.448489
8	1.998688	-0.226038	-2.292781
1	1.368752	-0.943402	-2.187442
1	1.562674	0.528103	-1.850241

Cartesian coordinates of : IMZW9_24

Atomic number (AN) and Cartesian coordinates

AN	X	Y	Z
6	3.271203	-1.075994	0.502810
6	2.194445	-1.777055	0.965606
6	1.780526	-0.973951	-1.024196
1	4.215037	-0.907651	0.992833
1	2.018576	-2.311861	1.881164
1	0.317985	-2.123189	-0.025307
1	1.243192	-0.766220	-1.933961
7	3.009846	-0.575674	-0.751921
7	1.252216	-1.702369	-0.022982
8	-1.568713	-0.719665	-2.261619
1	-1.763723	-0.770471	-3.198503
1	-1.058605	0.108685	-2.144340
8	1.794046	1.592638	1.671242
1	2.213432	0.725158	1.680276
1	1.935370	1.901762	0.759272
8	-2.393533	-1.053075	1.787377
1	-2.791643	-1.356713	2.604439
1	-1.802948	-0.318399	2.040081
8	-1.418306	-2.702553	-0.261165
1	-1.531792	-2.095370	-1.009712
1	-1.785720	-2.207448	0.490291
8	2.308479	2.183050	-1.041790
1	2.923073	2.865421	-1.317032
1	2.796447	1.343347	-1.108272
8	-0.826424	1.158948	2.285185
1	-0.945993	1.553866	3.150383
1	0.118978	1.302689	2.065014
8	-0.395801	1.714271	-1.797426
1	0.515030	1.877873	-1.508745
1	-0.967709	1.997617	-1.058729
8	-2.280936	2.303089	0.181740
1	-2.845739	1.552333	-0.075550
1	-1.805233	1.969145	0.962615
8	-3.705354	-0.058194	-0.514471
1	-3.048175	-0.318357	-1.182150

```
  1     -3.368977     -0.461993      0.302352
 ---------------------------------------------------

Cartesian coordinates of :  IMZW9_25

 ---------------------------------------------------
 Atomic number (AN) and Cartesian coordinates
  AN          X             Y             Z
 ---------------------------------------------------
  0      0.000000      0.000000      0.000000
  0      0.000000      0.000000      0.000000
  0      0.000000      0.000000      0.000000
  0      0.000000      0.000000      0.000000
  0      0.000000      0.000000      0.000000
  0      0.000000      0.000000      0.000000
  0      0.000000      0.000000      0.000000
  0      0.000000      0.000000      0.000000
  0      0.000000      0.000000      0.000000
  0      0.000000      0.000000      0.000000
  0      0.000000      0.000000      0.000000
  0      0.000000      0.000000      0.000000
  0      0.000000      0.000000      0.000000
  0      0.000000      0.000000      0.000000
  0      0.000000      0.000000      0.000000
  0      0.000000      0.000000      0.000000
  0      0.000000      0.000000      0.000000
  0      0.000000      0.000000      0.000000
  0      0.000000      0.000000      0.000000
  0      0.000000      0.000000      0.000000
  0      0.000000      0.000000      0.000000
  0      0.000000      0.000000      0.000000
  0      0.000000      0.000000      0.000000
  0      0.000000      0.000000      0.000000
  0      0.000000      0.000000      0.000000
  0      0.000000      0.000000      0.000000
  0      0.000000      0.000000      0.000000
  0      0.000000      0.000000      0.000000
  0      0.000000      0.000000      0.000000
  0      0.000000      0.000000      0.000000
  0      0.000000      0.000000      0.000000
  0      0.000000      0.000000      0.000000
  0      0.000000      0.000000      0.000000
  0      0.000000      0.000000      0.000000
  0      0.000000      0.000000      0.000000
  0      0.000000      0.000000      0.000000
 ---------------------------------------------------

Cartesian coordinates of :  IMZW9_26

 ---------------------------------------------------
 Atomic number (AN) and Cartesian coordinates
  AN          X             Y             Z
 ---------------------------------------------------
  6     -0.897286      1.948615     -1.212871
```

```
  6      0.456284      2.095602     -1.128418
  6     -0.054805      0.005310     -1.528097
  1     -1.658581      2.703297     -1.111333
  1      1.081105      2.950657     -0.946023
  1      1.963685      0.587959     -1.309207
  1      0.081075     -1.045003     -1.721972
  7     -1.212579      0.637174     -1.473337
  7      0.976896      0.850059     -1.347195
  8      1.536924      0.075546      1.884297
  1      1.706987     -0.680675      1.294866
  1      0.594789      0.275785      1.777918
  8     -3.618337     -0.510170     -0.779940
  1     -2.799553     -0.121648     -1.178075
  1     -4.214411     -0.669796     -1.513351
  8      3.733142     -0.006805     -0.988966
  1      3.776370      0.649383     -0.270934
  1      3.348175     -0.790698     -0.565420
  8      3.383892      1.939346      1.026229
  1      2.691286      1.344830      1.379825
  1      4.071676      1.921286      1.693943
  8     -1.868270     -2.016019      0.865323
  1     -2.547243     -1.690929      0.253529
  1     -1.557818     -1.199614      1.295762
  8      0.014226     -3.390237     -0.602839
  1      0.014632     -4.271644     -0.225550
  1     -0.655131     -2.902645     -0.078878
  8     -1.243109      0.492503      2.023923
  1     -2.037111      0.954007      1.684138
  1     -1.393735      0.414527      2.967875
  8     -3.590839      1.610071      1.051360
  1     -3.354687      2.390787      0.546235
  1     -3.751477      0.929391      0.373184
  8      2.281181     -2.090537      0.251711
  1      1.497545     -2.585755     -0.069511
  1      2.736557     -2.700881      0.834223
 ---------------------------------------------------

Cartesian coordinates of : IMZW10_ 1

 ---------------------------------------------------
 Atomic number (AN) and Cartesian coordinates
  AN          X             Y             Z
 ---------------------------------------------------
  6     -1.846776      2.714412      0.209386
  6     -2.697042      2.051553     -0.628754
  6     -1.672586      0.634163      0.683420
  1     -1.661388      3.772945      0.272044
  1     -3.362147      2.394159     -1.400070
  1     -3.060017     -0.067635     -0.736617
  1     -1.393657     -0.307106      1.127534
  7     -1.210700      1.819409      1.036431
  7     -2.577482      0.727832     -0.308193
  8      1.355967     -1.075781      1.432611
  1      1.301172     -0.181550      1.817476
```

1	0.996489	-0.977041	0.528411
8	-0.825566	-2.479724	2.480642
1	-0.505359	-3.326679	2.795916
1	-0.017595	-2.006608	2.203246
8	2.586651	1.838545	-0.125897
1	3.100833	1.015946	-0.110697
1	2.185045	1.863358	0.757778
8	1.213732	1.578307	2.325265
1	1.247690	1.912068	3.222655
1	0.308340	1.782567	1.988603
8	-3.701972	-1.721217	-1.238387
1	-2.904718	-2.184188	-0.907439
1	-3.623297	-1.770413	-2.192613
8	0.482426	1.708742	-1.941982
1	1.212927	1.808800	-1.298774
1	-0.253546	2.183782	-1.542157
8	3.018430	-1.104952	-2.465771
1	2.972211	-0.207070	-2.802087
1	3.427339	-1.001960	-1.588505
8	3.821452	-0.722410	0.164834
1	3.078047	-0.976680	0.742077
1	4.618266	-0.921448	0.658701
8	0.488575	-0.999130	-1.237054
1	1.328403	-1.179800	-1.698236
1	0.309102	-0.068565	-1.467486
8	-1.375385	-2.869291	-0.280246
1	-0.732736	-2.239174	-0.646643
1	-1.270172	-2.773876	0.682789

Cartesian coordinates of : IMZW10_ 2

Atomic number (AN) and Cartesian coordinates

AN	X	Y	Z
6	1.271551	-2.699985	1.151745
6	-0.023959	-2.698281	1.583302
6	0.535885	-0.707712	0.867273
1	1.970631	-3.518372	1.140364
1	-0.648415	-3.468296	1.997993
1	-1.422279	-1.079357	1.570319
1	0.442135	0.336310	0.607427
7	1.618816	-1.447789	0.708909
7	-0.471561	-1.418682	1.407903
8	-0.357981	-2.433563	-1.819116
1	0.005291	-2.812217	-1.012270
1	0.271294	-1.722477	-2.061050
8	-2.441874	1.659828	-1.184107
1	-2.534156	0.690671	-1.220027
1	-2.316760	1.853120	-0.239627
8	2.904825	1.767302	1.631646
1	2.556208	1.098080	2.224861
1	3.141551	1.263523	0.832149

8	0.150943	2.027293	-1.940324
1	0.191142	2.683256	-2.638867
1	-0.807256	1.914115	-1.750337
8	-1.814259	2.155874	1.560618
1	-0.919351	2.468580	1.303923
1	-2.278133	2.942923	1.850912
8	-3.139830	-0.353815	1.511578
1	-3.153145	-0.594874	0.572217
1	-2.764859	0.540155	1.521264
8	3.327643	0.191088	-0.688697
1	4.234076	-0.107376	-0.783921
1	2.872711	-0.522845	-0.180417
8	0.609518	3.048753	0.647065
1	0.542787	2.733956	-0.271242
1	1.390872	2.588819	1.006226
8	-2.707419	-1.128933	-1.186552
1	-1.876369	-1.603420	-1.399800
1	-3.345327	-1.435294	-1.832976
8	1.314659	-0.393660	-2.666460
1	0.840412	0.438083	-2.481251
1	2.105505	-0.307220	-2.112672

Cartesian coordinates of : IMZW10_ 3

Atomic number (AN) and Cartesian coordinates

AN	X	Y	Z
6	-0.249060	2.987782	-1.172878
6	1.046801	2.580404	-1.294116
6	0.345889	2.276336	0.755538
1	-0.891549	3.402670	-1.930460
1	1.725303	2.563909	-2.127415
1	2.288006	1.678987	0.198971
1	0.363513	1.996711	1.795249
7	-0.683225	2.803021	0.118177
7	1.414419	2.142376	-0.051047
8	-2.907065	1.547142	1.197399
1	-2.226826	2.165353	0.847283
1	-3.524146	2.089108	1.691235
8	-0.338384	-0.590281	-1.177225
1	-0.466038	-1.554298	-1.233688
1	-1.214690	-0.218024	-1.383516
8	-1.147872	-0.637644	1.557720
1	-0.810075	-0.569817	0.644824
1	-1.738079	0.131175	1.634226
8	2.401147	-0.699917	-1.718610
1	1.446438	-0.516518	-1.718228
1	2.588058	-1.067437	-2.584082
8	1.511914	-0.727250	2.354505
1	1.613550	-1.093467	3.234553
1	0.551311	-0.610778	2.231812
8	3.563481	0.336007	0.680305

1	3.351330	-0.093766	-0.160343
1	2.927498	-0.055021	1.299331
8	-0.430575	-3.400896	-0.905624
1	-0.185600	-3.891865	-1.691534
1	0.410611	-3.244195	-0.427643
8	1.933309	-2.669096	0.313266
1	1.764289	-2.067598	1.059018
1	2.183575	-2.067130	-0.407009
8	-3.091670	0.067133	-1.236055
1	-3.143157	-0.830677	-0.863967
1	-3.141660	0.630369	-0.446884
8	-2.900142	-2.437218	0.138437
1	-2.451177	-1.896441	0.806758
1	-2.153773	-2.870017	-0.302618

Cartesian coordinates of : IMZW10_ 4

Atomic number (AN) and Cartesian coordinates

AN	X	Y	Z
6	0.249757	3.139616	-0.382422
6	1.397315	2.495988	-0.750864
6	-0.340732	1.230423	-1.148021
1	0.144002	4.122792	0.042672
1	2.431223	2.786729	-0.713151
1	1.586860	0.534217	-1.620399
1	-0.913367	0.376170	-1.471067
7	-0.840517	2.342298	-0.640908
7	1.000930	1.284668	-1.244411
8	0.340174	0.944474	2.109492
1	0.370834	1.721879	1.541557
1	-0.604755	0.675711	2.100470
8	-2.265457	0.078318	2.027650
1	-2.225957	-0.859539	1.774922
1	-2.721496	0.510188	1.284941
8	3.338446	-1.636384	0.204271
1	3.416475	-0.879687	0.819965
1	3.941312	-2.303688	0.535448
8	-3.393438	1.329920	-0.238767
1	-4.151553	1.912232	-0.174652
1	-2.613371	1.910586	-0.368023
8	-2.963187	-1.328740	-1.185405
1	-2.754321	-1.786681	-0.358424
1	-3.129206	-0.412882	-0.913538
8	2.247012	-1.057320	-2.338064
1	2.677184	-1.342321	-1.516204
1	1.369658	-1.471900	-2.297682
8	-1.965135	-2.607784	1.173600
1	-2.155251	-3.298846	1.809698
1	-0.998517	-2.524290	1.153867
8	0.614855	-1.655893	0.834141
1	1.560999	-1.803700	0.656814

```
  1      0.565199     -0.737710      1.144160
  8      3.177882      0.557369      1.863829
  1      3.569181      0.375565      2.720425
  1      2.225524      0.583353      2.038905
  8     -0.297763     -2.224465     -1.778161
  1     -0.027995     -2.020462     -0.865510
  1     -1.190695     -1.848617     -1.820787
 ----------------------------------------------------
```

Cartesian coordinates of : IMZW10_ 5

```
 ----------------------------------------------------
 Atomic number (AN) and Cartesian coordinates
  AN          X             Y             Z
 ----------------------------------------------------
  6     -2.739184      1.666270     -1.168386
  6     -1.539567      2.268456     -1.427034
  6     -1.270189      0.112885     -1.190454
  1     -3.709333      2.124793     -1.083178
  1     -1.269334      3.294609     -1.598031
  1      0.395199      1.319128     -1.585884
  1     -0.770001     -0.841475     -1.152221
  7     -2.565550      0.309877     -1.025854
  7     -0.617182      1.260569     -1.447474
  8     -1.403304      1.681301      1.890587
  1     -1.899124      1.905050      1.096061
  1     -1.586380      0.731923      2.029500
  8     -1.783141     -1.049978      2.412963
  1     -0.841345     -1.193582      2.252194
  1     -2.218792     -1.489706      1.662504
  8      3.439940      1.286141      0.684134
  1      2.635873      1.770743      0.957305
  1      4.085875      1.967585      0.490549
  8     -2.957631     -2.214683      0.102051
  1     -3.822362     -2.626500      0.133482
  1     -3.088121     -1.347790     -0.334604
  8     -0.209071     -3.002472      0.024945
  1      0.095756     -2.272435      0.588664
  1     -1.162002     -2.843023     -0.055751
  8      2.150342      0.788307     -1.816405
  1      2.638955      0.945401     -0.991201
  1      2.000660     -0.170355     -1.828828
  8      0.905671     -0.804039      1.512203
  1      1.794699     -1.092569      1.225101
  1      0.733656     -0.010471      0.997211
  8      3.382776     -1.555739      0.449701
  1      2.945580     -1.745271     -0.397567
  1      3.592326     -0.611588      0.408784
  8      1.109661      2.689282      1.212764
  1      1.295179      3.308346      1.921110
  1      0.274521      2.261246      1.482620
  8      1.688347     -2.017899     -1.775758
  1      1.912185     -2.632434     -2.476313
  1      0.950377     -2.432176     -1.290460
```

Cartesian coordinates of : IMZW10_ 6

Atomic number (AN) and Cartesian coordinates

AN	X	Y	Z
6	-0.539793	3.051675	-0.366788
6	0.811748	3.212218	-0.311597
6	0.302590	1.085390	-0.188311
1	-1.301608	3.806686	-0.456767
1	1.438034	4.084510	-0.340823
1	2.309041	1.674414	-0.129443
1	0.437745	0.017714	-0.105721
7	-0.856429	1.714835	-0.289959
7	1.329541	1.950911	-0.198983
8	-3.644999	-1.214672	0.400720
1	-3.406048	-0.535293	1.051044
1	-3.506925	-0.751461	-0.442595
8	-0.470265	-1.448994	-2.565284
1	-1.110530	-0.725143	-2.478380
1	0.405275	-1.054174	-2.430352
8	2.266781	-0.479973	2.259088
1	1.297049	-0.533868	2.387566
1	2.641394	-0.940498	3.011893
8	-2.668744	0.191396	-1.840860
1	-3.161366	0.765011	-2.430858
1	-2.145743	0.799383	-1.276651
8	2.297335	-0.937726	-1.965320
1	2.778612	-1.448170	-2.619619
1	2.123598	-1.571125	-1.237412
8	3.791513	0.479632	0.043711
1	3.446686	-0.034493	-0.703678
1	3.353723	0.086005	0.814080
8	-1.100947	-2.361037	0.028790
1	-2.009264	-2.043453	0.193875
1	-0.897465	-2.048412	-0.873226
8	-0.443426	-0.929111	2.450484
1	-1.091378	-0.206855	2.427180
1	-0.591069	-1.385334	1.605971
8	-2.528058	0.902409	1.911375
1	-2.071924	1.269056	1.131259
1	-2.904619	1.660006	2.362716
8	1.794272	-2.484963	0.263943
1	0.830958	-2.536924	0.317411
1	2.029065	-1.839171	0.952128

Cartesian coordinates of : IMZW10_ 7

Atomic number (AN) and Cartesian coordinates

AN X Y Z

```
 ---------------------------------------------------
  6      2.551216      1.846476     -1.125354
  6      3.136344      0.617368     -1.201010
  6      1.036419      0.416621     -0.617838
  1      2.993039      2.809902     -1.312605
  1      4.133289      0.305897     -1.452765
  1      2.224454     -1.296214     -0.821171
  1      0.109566     -0.050386     -0.319838
  7      1.231808      1.716475     -0.763143
  7      2.154763     -0.280288     -0.882068
  8      0.055903      2.527745      1.837148
  1      0.415768      2.487538      0.938054
  1     -0.875822      2.268844      1.709843
  8     -2.593562      2.002398      1.107146
  1     -2.854874      1.070383      1.027214
  1     -2.309329      2.247541      0.211796
  8      0.949863     -2.007766      2.049106
  1      1.259963     -1.095199      2.228333
  1      1.121748     -2.492172      2.858292
  8     -1.388558      2.589276     -1.368371
  1     -1.458151      3.339548     -1.960758
  1     -0.432488      2.476017     -1.193245
  8     -2.339035     -0.032316     -1.855599
  1     -2.755082     -0.207335     -0.993734
  1     -1.976818      0.864992     -1.767178
  8      1.648222     -3.078906     -0.479519
  1      1.434442     -2.810678      0.429874
  1      0.833412     -2.896912     -0.970552
  8     -3.320612     -0.666948      0.720570
  1     -4.195928     -1.046713      0.807703
  1     -2.693391     -1.413291      0.836402
  8     -1.477654     -2.666679      0.809836
  1     -1.216023     -2.609827     -0.124604
  1     -0.682601     -2.384395      1.294085
  8      1.867242      0.536240      2.582198
  1      1.178123      1.193758      2.362773
  1      2.559716      0.710467      1.940648
  8     -0.706342     -2.265241     -1.919927
  1     -1.207367     -2.879350     -2.459400
  1     -1.219255     -1.430936     -1.948169
 ---------------------------------------------------

Cartesian coordinates of : IMZW10_ 8

 ---------------------------------------------------
 Atomic number (AN) and Cartesian coordinates
  AN          X             Y             Z
 ---------------------------------------------------
  6     -4.136008     -0.507223     -0.041597
  6     -3.516893     -1.496208     -0.749513
  6     -2.243732     -0.826395      0.902247
  1     -5.106076     -0.074020     -0.215023
  1     -3.812804     -2.065952     -1.611025
  1     -1.560853     -2.304033     -0.406506
```

1	-1.389933	-0.775639	1.556406
7	-3.337600	-0.094827	1.000390
7	-2.316112	-1.693857	-0.124606
8	0.184115	0.238355	-1.501872
1	0.960506	0.638399	-1.924736
1	-0.589139	0.797362	-1.708096
8	3.055426	2.008338	0.454346
1	2.130405	1.919717	0.739136
1	3.028828	1.747434	-0.481852
8	0.604529	-2.282471	-0.389856
1	0.396763	-1.445190	-0.839395
1	0.802495	-1.992371	0.521302
8	1.304269	-1.176660	2.114572
1	0.896016	-0.319248	1.906634
1	2.250758	-1.003588	1.979663
8	0.404367	1.300082	1.091257
1	0.308478	0.909097	0.201230
1	-0.427659	1.785290	1.221536
8	-2.153484	2.465751	1.063573
1	-2.572598	3.207630	1.502828
1	-2.724153	1.692647	1.241201
8	3.318572	-1.576516	-1.067732
1	3.635799	-1.262654	-0.203753
1	2.428634	-1.916971	-0.876439
8	2.802552	0.915201	-2.137261
1	3.049623	0.006627	-1.863855
1	3.302429	1.089273	-2.936148
8	-2.150110	1.727749	-1.692560
1	-2.814734	1.032426	-1.653315
1	-2.158208	2.098427	-0.793769
8	3.935854	-0.419577	1.412871
1	4.744873	-0.366879	1.923697
1	3.727614	0.499937	1.144269

Cartesian coordinates of : IMZW10_ 9

Atomic number (AN) and Cartesian coordinates

AN	X	Y	Z
6	-3.219533	-1.962144	0.185271
6	-2.946608	-0.957021	1.066713
6	-2.849398	-0.218694	-0.988549
1	-3.456060	-2.989491	0.403881
1	-2.897089	-0.926756	2.140228
1	-2.433990	1.076999	0.635558
1	-2.714341	0.472172	-1.803394
7	-3.162767	-1.495930	-1.108266
7	-2.719752	0.153163	0.300674
8	0.314331	-1.574059	1.821370
1	0.036098	-1.750150	0.902115
1	-0.296834	-2.064979	2.373599
8	3.057667	-1.466186	1.126483

1	2.971854	-1.685426	0.182702
1	2.165094	-1.600663	1.480690
8	0.408047	0.949259	-0.970987
1	1.341226	0.990173	-1.262492
1	0.447827	1.090814	-0.008539
8	-0.015838	-1.778783	-0.928490
1	-0.897220	-1.990145	-1.254922
1	0.050303	-0.804492	-1.014647
8	2.670303	-1.918508	-1.640302
1	1.711688	-2.066948	-1.586438
1	3.026324	-2.685361	-2.091701
8	-1.218406	3.115565	-1.609371
1	-0.673117	3.894755	-1.730325
1	-0.577158	2.387061	-1.506085
8	3.291941	1.257177	1.131626
1	3.301619	0.277865	1.202424
1	4.028327	1.555266	1.668550
8	0.583151	1.214228	1.899721
1	0.458422	0.250678	1.970222
1	1.541177	1.330084	1.777556
8	-1.695580	2.718179	1.157993
1	-0.861424	2.302233	1.428159
1	-1.536089	2.984003	0.235441
8	3.113465	0.882359	-1.684769
1	3.366752	1.071666	-0.767256
1	3.094825	-0.086685	-1.728983

Cartesian coordinates of : IMZW10_10

Atomic number (AN) and Cartesian coordinates

AN	X	Y	Z
6	3.608713	-0.050755	1.111223
6	3.272799	1.267625	0.999209
6	2.491172	0.138887	-0.703157
1	4.171984	-0.530748	1.892953
1	3.470495	2.119797	1.623447
1	2.117047	2.210618	-0.539022
1	1.982260	-0.061422	-1.631544
7	3.123259	-0.754012	0.035875
7	2.572687	1.372966	-0.171305
8	-0.111300	-2.130126	-0.295697
1	0.695830	-2.653525	-0.461675
1	0.145959	-1.485094	0.385988
8	-0.964916	-0.369856	-2.304448
1	-1.930886	-0.404336	-2.217515
1	-0.649765	-1.048522	-1.678746
8	-3.468645	1.690351	0.557241
1	-3.254020	1.115774	1.309346
1	-3.727915	1.069542	-0.142862
8	2.367129	-3.367552	-0.654105
1	2.754400	-2.493122	-0.420500

```
  1      2.540839     -3.460543     -1.592411
  8     -2.837025     -1.965753      0.471889
  1     -2.747324     -1.369196      1.235181
  1     -1.923102     -2.163226      0.215661
  8      0.954727      3.567971     -1.112853
  1      0.215236      2.968514     -0.896796
  1      0.970318      3.588550     -2.071265
  8     -3.690247     -0.264471     -1.493892
  1     -3.471956     -0.946418     -0.822482
  1     -4.473622     -0.591621     -1.939328
  8      0.136620     -0.105505      1.651695
  1     -0.089001      0.577313      0.985015
  1      0.989057      0.159024      2.008809
  8     -0.796713      1.591466     -0.306780
  1     -1.728026      1.710373     -0.036164
  1     -0.834638      0.955335     -1.048541
  8     -2.482609     -0.072467      2.539195
  1     -2.675268     -0.272385      3.456560
  1     -1.511801     -0.054367      2.472301
 ---------------------------------------------------

Cartesian coordinates of : IMZW10_11

 ---------------------------------------------------
 Atomic number (AN) and Cartesian coordinates
  AN          X             Y             Z
 ---------------------------------------------------
  6      0.951888      2.308362     -1.584967
  6     -0.142242      2.881279     -1.002398
  6     -0.192039      0.710016     -0.733693
  1      1.754672      2.789393     -2.116900
  1     -0.470026      3.901939     -0.929735
  1     -1.775588      1.886206     -0.015251
  1     -0.547330     -0.257688     -0.419016
  7      0.911640      0.944623     -1.417661
  7     -0.858911      1.848445     -0.465654
  8      2.553789     -1.210489     -1.881622
  1      2.008095     -0.385107     -1.826506
  1      2.934817     -1.203880     -2.761406
  8      3.559851     -0.468871      0.678289
  1      3.416643     -0.631523     -0.266512
  1      3.130492      0.385563      0.845196
  8     -3.374287     -0.440430     -1.639309
  1     -4.239043     -0.788751     -1.862880
  1     -2.965747     -1.143374     -1.094990
  8      2.213543      1.966769      1.375023
  1      2.677987      2.606495      1.918744
  1      1.869805      2.478929      0.634303
  8      0.520556      0.106582      2.678946
  1      0.795155     -0.695372      2.196316
  1      1.003321      0.821045      2.233423
  8     -3.496577      1.345499      0.555947
  1     -3.098413      0.754440      1.218146
  1     -3.582106      0.767379     -0.223379
```

8	1.383099	-2.169454	1.300480
1	2.183126	-1.668528	1.056239
1	0.949653	-2.354483	0.452855
8	-2.158843	-2.310388	0.036605
1	-1.276987	-2.420348	-0.348266
1	-2.035010	-1.720188	0.801218
8	0.301910	-2.744675	-1.344864
1	1.103463	-2.276931	-1.650473
1	0.516849	-3.675749	-1.424400
8	-2.101160	-0.466812	2.157950
1	-2.450833	-0.813482	2.980572
1	-1.192785	-0.167253	2.372703

Cartesian coordinates of : IMZW10_12

Atomic number (AN) and Cartesian coordinates

AN	X	Y	Z
6	2.268738	-2.154993	1.265464
6	2.935310	-0.979733	1.468897
6	2.347671	-1.167713	-0.627419
1	2.038000	-2.921389	1.985419
1	3.372759	-0.539573	2.346308
1	3.363995	0.558631	0.026702
1	2.237294	-0.911895	-1.667937
7	1.905897	-2.272669	-0.055995
7	2.982411	-0.365536	0.247667
8	-3.238827	0.666150	-1.489618
1	-3.285868	1.156983	-0.642458
1	-3.826282	1.126030	-2.090987
8	-0.442002	-2.551168	-1.730375
1	0.298886	-2.678671	-1.111274
1	-0.424961	-1.589226	-1.871488
8	-0.473104	0.288617	-1.567038
1	-1.417162	0.503338	-1.673017
1	-0.382159	0.132007	-0.608663
8	1.053718	2.644100	-1.216688
1	0.624474	2.847458	-0.369739
1	0.596433	1.828219	-1.485526
8	-3.178023	1.794863	1.020099
1	-3.086687	0.931915	1.454110
1	-2.296005	2.194632	1.100859
8	3.713398	2.261196	-0.587346
1	2.785982	2.413691	-0.863725
1	4.194431	2.168176	-1.411387
8	-0.477263	2.713679	1.167702
1	-0.257072	1.777568	1.336355
1	-0.236861	3.173770	1.974153
8	-2.756072	-0.871953	1.869014
1	-2.990923	-1.462710	2.585949
1	-2.879153	-1.387465	1.042906
8	-2.989825	-2.009832	-0.607217

1	-2.103130	-2.270487	-0.914535
1	-3.139684	-1.147192	-1.027721
8	-0.229498	-0.017275	1.233793
1	0.482012	-0.569654	1.575892
1	-1.056807	-0.399104	1.587845

Cartesian coordinates of : IMZW10_13

Atomic number (AN) and Cartesian coordinates

AN	X	Y	Z
6	-2.459372	2.086191	1.146781
6	-3.219752	0.983403	0.883517
6	-1.877564	1.393861	-0.792164
1	-2.470027	2.709610	2.024175
1	-3.977333	0.475482	1.452155
1	-3.123504	-0.311250	-0.817840
1	-1.394788	1.279909	-1.748071
7	-1.622230	2.344947	0.086996
7	-2.844050	0.561792	-0.362755
8	0.118452	-1.152583	-1.269066
1	-0.188225	-0.819615	-0.409689
1	0.600958	-0.396898	-1.655568
8	-3.273326	-2.063153	-1.432376
1	-2.725707	-2.051206	-2.219844
1	-2.690318	-2.443548	-0.746075
8	1.646000	0.975459	-2.291031
1	2.457004	0.543127	-1.982164
1	1.472433	1.664899	-1.626090
8	3.100267	1.303678	0.926945
1	2.388734	1.849440	0.552538
1	3.398001	0.746751	0.187378
8	-1.675937	-3.049705	0.648413
1	-1.278821	-2.234275	1.002486
1	-0.941356	-3.486126	0.211333
8	2.186521	-2.465227	0.126581
1	2.154709	-1.998640	0.976167
1	1.434117	-2.092192	-0.370093
8	-0.500992	-0.635306	1.441779
1	-1.036585	0.077225	1.803253
1	0.349401	-0.611793	1.922118
8	3.747342	-0.520296	-1.117382
1	4.643841	-0.785284	-1.327716
1	3.326287	-1.302272	-0.708797
8	1.094044	2.865538	-0.271467
1	0.127513	2.823597	-0.094246
1	1.280141	3.784578	-0.469875
8	2.057165	-0.742878	2.396524
1	2.345831	-0.717503	3.309748
1	2.455493	0.043852	1.964859

Cartesian coordinates of : IMZW10_14

Atomic number (AN) and Cartesian coordinates

AN	X	Y	Z
6	3.765722	-0.702181	-0.555135
6	3.517446	0.635673	-0.470817
6	2.547705	-0.422708	1.180278
1	4.341429	-1.226820	-1.298131
1	3.810213	1.468294	-1.084326
1	2.299003	1.660290	0.949793
1	1.956477	-0.579561	2.066375
7	3.161925	-1.364946	0.488626
7	2.751690	0.797940	0.651673
8	0.212026	-0.480684	-1.257698
1	0.511533	-1.369245	-1.003045
1	-0.712405	-0.604183	-1.534187
8	-1.794447	-2.866632	0.111399
1	-0.827811	-2.906747	0.057583
1	-2.048259	-2.278141	-0.619999
8	-2.122195	2.509127	-0.394609
1	-1.618346	1.943888	0.227040
1	-2.156423	3.365122	0.037947
8	1.024346	-3.066332	-0.258539
1	1.285630	-3.616675	-0.999079
1	1.852007	-2.646080	0.049650
8	0.961300	3.070927	1.117243
1	0.868027	3.002510	0.151663
1	0.340134	2.395289	1.421774
8	0.481374	2.372550	-1.632082
1	0.598395	1.418256	-1.519605
1	-0.454901	2.501102	-1.412825
8	-2.707353	-1.022818	1.928405
1	-2.846646	-1.366069	2.812173
1	-2.376867	-1.776583	1.396786
8	-0.680784	0.722740	1.159733
1	-1.355469	0.124979	1.530887
1	-0.311216	0.242971	0.395361
8	-4.126627	0.522778	0.031427
1	-3.746919	0.048800	0.788990
1	-3.583983	1.324084	-0.040897
8	-2.510269	-0.865696	-1.743988
1	-3.148389	-0.366803	-1.193701
1	-2.954724	-1.022017	-2.578611

Cartesian coordinates of : IMZW10_15

Atomic number (AN) and Cartesian coordinates

AN	X	Y	Z
6	0.819616	3.222311	0.047448

6	-0.521097	3.426154	-0.082555
6	-0.093720	1.280896	0.007526
1	1.606484	3.954506	0.103782
1	-1.112377	4.319912	-0.156996
1	-2.072972	1.932133	-0.173350
1	-0.268743	0.215401	0.015752
7	1.085508	1.873955	0.104678
7	-1.084464	2.179856	-0.108083
8	2.655470	0.466800	-1.845448
1	2.259539	1.056659	-1.177190
1	3.225296	1.026020	-2.376222
8	3.190362	-1.551968	0.016690
1	3.101764	-0.986256	0.801488
1	3.146576	-0.911836	-0.713821
8	-2.357513	-0.819307	-2.158732
1	-1.413499	-0.705670	-2.358943
1	-2.335342	-1.470721	-1.432974
8	2.521141	0.315381	1.997080
1	2.153819	0.955523	1.354642
1	3.016665	0.839393	2.628573
8	0.277834	-1.343592	2.410744
1	0.384048	-1.892662	1.619752
1	1.025961	-0.724398	2.362613
8	-3.580107	0.798922	-0.172197
1	-3.200966	0.298187	-0.915764
1	-3.228149	0.345536	0.608658
8	0.740827	-2.715272	-0.128143
1	1.650146	-2.339085	-0.072525
1	0.866078	-3.665700	-0.162326
8	-2.106576	-2.439359	0.111216
1	-1.141014	-2.488581	0.061165
1	-2.267689	-1.762134	0.787680
8	0.426000	-1.142710	-2.543525
1	0.475590	-1.749646	-1.788842
1	1.164608	-0.530843	-2.393180
8	-2.286274	-0.356516	2.118899
1	-1.353334	-0.615667	2.267137
1	-2.767920	-0.751929	2.847554

Cartesian coordinates of : IMZW10_16

Atomic number (AN) and Cartesian coordinates

AN	X	Y	Z
6	0.541531	2.817721	1.065130
6	-0.524838	3.036879	0.240347
6	-0.289955	0.894448	0.619464
1	1.206652	3.541645	1.503603
1	-0.954665	3.936442	-0.160417
1	-1.896221	1.582667	-0.549055
1	-0.484935	-0.165997	0.577006
7	0.679361	1.471434	1.304311

```
  7     -1.042144      1.801607     -0.032643
  8     -3.421759     -0.177968      1.657712
  1     -2.892531     -0.954779      1.382690
  1     -4.258639     -0.551404      1.939214
  8      1.676874     -2.362893     -1.027937
  1      2.374868     -1.726458     -0.757119
  1      2.151840     -3.111150     -1.394666
  8      2.129169      1.804067     -1.571545
  1      2.631079      2.362533     -2.168839
  1      1.676060      2.420467     -0.984437
  8      0.688461     -2.608645      1.602834
  1      1.040474     -2.635185      0.696223
  1      1.180323     -1.872947      2.004239
  8      2.337546     -0.449626      2.370408
  1      2.729006     -0.226695      3.216360
  1      1.780128      0.326668      2.120447
  8     -1.782516     -1.267015     -1.928998
  1     -0.939071     -0.864885     -2.221142
  1     -2.024111     -1.873260     -2.631298
  8      0.688245     -0.344500     -2.734392
  1      1.098136     -1.084705     -2.256111
  1      1.093251      0.442359     -2.335105
  8      3.407061     -0.387926     -0.245739
  1      3.167658     -0.352742      0.695381
  1      2.989841      0.397185     -0.635836
  8     -1.948538     -2.310864      0.681925
  1     -1.814634     -1.998948     -0.229877
  1     -1.050240     -2.358021      1.057285
  8     -3.523328      0.722129     -1.026525
  1     -3.012440      0.003113     -1.437363
  1     -3.622493      0.424448     -0.104437
 ---------------------------------------------------

Cartesian coordinates of : IMZW10_17

 ---------------------------------------------------
 Atomic number (AN) and Cartesian coordinates
  AN          X             Y             Z
 ---------------------------------------------------
  6      0.470364      2.754183      1.226594
  6     -0.584484      3.015994      0.399534
  6     -0.294481      0.855909      0.593987
  1      1.104046      3.454419      1.743128
  1     -1.032636      3.935334      0.070076
  1     -1.892897      1.600181     -0.551228
  1     -0.450473     -0.200291      0.445252
  7      0.642737      1.396444      1.349782
  7     -1.059216      1.796047      0.006793
  8      1.742665     -2.285877     -1.166855
  1      2.425098     -1.643364     -0.872072
  1      2.233892     -2.999807     -1.577777
  8      3.421346     -0.306713     -0.289470
  1      3.188226     -0.330664      0.653409
  1      2.986631      0.491940     -0.630478
```

8	-1.807870	-1.322573	-1.958524
1	-1.709282	-1.681532	-1.059774
1	-0.974049	-0.854354	-2.138392
8	2.101014	1.933051	-1.471555
1	2.591446	2.512477	-2.058341
1	1.658363	2.528246	-0.855832
8	2.359134	-0.519194	2.320990
1	1.774110	0.247734	2.104594
1	2.729905	-0.328991	3.184065
8	-3.486469	0.754724	-1.145886
1	-3.644956	0.445063	-0.236763
1	-2.958046	0.027820	-1.528014
8	0.771539	-2.670131	1.447477
1	1.120114	-2.645517	0.538984
1	1.246826	-1.942575	1.883843
8	-1.841404	-2.311496	0.696570
1	-0.932085	-2.430203	1.045553
1	-2.234242	-3.185830	0.720883
8	0.661197	-0.187712	-2.720135
1	1.074376	0.575145	-2.285783
1	1.091730	-0.952684	-2.303913
8	-3.513410	-0.224004	1.517725
1	-2.937442	-0.980856	1.304592
1	-4.353646	-0.626245	1.744580

Cartesian coordinates of : IMZW10_18

Atomic number (AN) and Cartesian coordinates

AN	X	Y	Z
6	1.965617	2.909479	0.092719
6	3.108789	2.170920	0.027872
6	1.364394	0.873721	-0.214868
1	1.860436	3.970707	0.238274
1	4.146591	2.439036	0.101485
1	3.313277	0.055177	-0.248153
1	0.784958	-0.023515	-0.358458
7	0.871796	2.091522	-0.061511
7	2.707619	0.877461	-0.168585
8	-1.101491	1.059009	1.986088
1	-0.543331	1.527114	1.350070
1	-1.999896	1.129714	1.612390
8	-3.608740	1.416360	0.755456
1	-3.718729	0.515762	0.412534
1	-3.124002	1.882355	0.058135
8	1.773828	-2.640415	0.224113
1	1.233045	-2.422032	-0.550852
1	1.331220	-2.169705	0.946986
8	-1.728820	2.674938	-0.950215
1	-1.677979	3.258325	-1.708969
1	-0.802569	2.568688	-0.635982
8	-1.864971	0.006534	-1.917139

1	-2.547827	-0.382839	-1.345411
1	-1.782983	0.925357	-1.608813
8	4.224653	-1.542251	-0.353133
1	3.397916	-2.002314	-0.100187
1	4.773814	-1.592574	0.431024
8	-3.671359	-1.256313	-0.118476
1	-4.314743	-1.789092	-0.587769
1	-2.943936	-1.870149	0.120842
8	-1.483432	-2.803582	0.421670
1	-0.953363	-2.345518	1.098202
1	-0.985854	-2.647160	-0.396996
8	0.170169	-1.366690	2.204925
1	0.092168	-1.649998	3.117194
1	-0.303398	-0.510214	2.158207
8	0.024071	-1.996942	-1.916255
1	-0.216332	-2.552679	-2.659317
1	-0.578447	-1.227233	-1.964379

Cartesian coordinates of : IMZW10_19

Atomic number (AN) and Cartesian coordinates

AN	X	Y	Z
6	-3.829317	-0.024046	0.215203
6	-3.445267	1.273128	0.407080
6	-2.082638	0.276863	-0.982155
1	-4.667256	-0.541668	0.649649
1	-3.856429	2.067080	1.003225
1	-1.776545	2.301395	-0.479670
1	-1.258088	0.142924	-1.662477
7	-2.974052	-0.643285	-0.663900
7	-2.333023	1.448965	-0.369008
8	2.623151	-1.585410	1.332083
1	1.793405	-1.974076	1.016628
1	3.119761	-1.404999	0.516279
8	1.188692	-0.857340	-1.684567
1	2.137568	-1.022366	-1.577365
1	0.784235	-1.280508	-0.907847
8	1.377602	1.661030	-0.448258
1	1.187590	1.379991	0.471360
1	1.262316	0.838372	-0.962459
8	-1.401968	-0.546952	2.370834
1	-1.661978	-0.994913	3.178469
1	-2.219902	-0.168024	2.028149
8	-1.807830	-3.093345	-1.419246
1	-2.340065	-2.296907	-1.200852
1	-1.290035	-2.820191	-2.179869
8	-0.009976	-2.207912	0.549422
1	-0.559291	-1.655822	1.131074
1	-0.633144	-2.623415	-0.075122
8	1.140217	0.692350	2.120497
1	0.260354	0.305115	2.248872

1	1.704832	-0.070385	1.895739
8	4.131730	1.573059	0.082632
1	3.179402	1.678926	-0.106536
1	4.153329	1.329795	1.010836
8	3.961974	-0.923905	-1.094181
1	4.797653	-1.366728	-1.248312
1	4.188784	-0.041855	-0.739618
8	-0.529192	3.617307	-0.820542
1	-0.603694	3.830824	-1.752182
1	0.194855	2.960975	-0.777777

Cartesian coordinates of : IMZW10_20

Atomic number (AN) and Cartesian coordinates

AN	X	Y	Z
6	0.652713	2.313945	-1.616534
6	-0.591984	2.668120	-1.183713
6	0.121418	0.923583	-0.072885
1	1.252274	2.777390	-2.381073
1	-1.260610	3.457261	-1.475152
1	-1.767212	1.778143	0.365168
1	0.134014	0.118884	0.644806
7	1.099565	1.220046	-0.913105
7	-0.910657	1.777667	-0.196052
8	2.697255	2.168736	1.504774
1	3.617557	2.403311	1.368488
1	2.370176	1.973568	0.616529
8	-1.048068	-0.373068	-2.947402
1	-0.676725	0.508798	-2.847715
1	-0.310255	-0.972419	-2.726229
8	-2.418936	-0.774207	2.020486
1	-2.480769	-0.909410	1.062631
1	-1.492415	-0.974358	2.227998
8	-2.638829	-1.117096	-0.794442
1	-2.118352	-0.787342	-1.555156
1	-3.320896	-1.669548	-1.180044
8	-3.227461	1.815572	1.509634
1	-2.999544	2.454088	2.187535
1	-2.945158	0.956297	1.876923
8	0.230743	-1.630420	2.524011
1	0.350856	-2.292055	3.206821
1	1.124456	-1.252829	2.367434
8	3.026259	-0.868568	-0.799361
1	2.490054	-0.054420	-0.887175
1	2.467110	-1.515956	-1.257348
8	0.924942	-2.199782	-2.126059
1	0.976727	-2.983995	-2.675472
1	0.447262	-2.488038	-1.318244
8	2.710800	-0.639779	1.971600
1	2.825911	-0.860302	1.027720
1	2.703178	0.332359	1.959633

8	-0.539445	-2.841344	0.092305
1	-0.237463	-2.434912	0.924500
1	-1.303501	-2.302762	-0.174061

Cartesian coordinates of : IMZW10_21

Atomic number (AN) and Cartesian coordinates

AN	X	Y	Z
6	2.343267	-2.570750	0.198297
6	3.286518	-1.726884	0.705305
6	2.061360	-0.682194	-0.774484
1	2.168177	-3.603954	0.444011
1	4.057890	-1.864649	1.440501
1	3.621897	0.336328	0.220299
1	1.702202	0.111325	-1.408119
7	1.578560	-1.911840	-0.736029
7	3.094895	-0.529553	0.072378
8	-3.761474	1.327259	-0.962127
1	-3.528353	1.602047	-0.051570
1	-4.565638	1.799489	-1.182199
8	-1.204714	0.880443	-2.070044
1	-1.135946	-0.091015	-2.054712
1	-2.129224	1.058978	-1.834961
8	1.959267	2.992348	-0.570336
1	1.820122	3.891034	-0.266861
1	1.239442	2.482821	-0.149365
8	-0.973053	-1.912663	-1.812034
1	-0.061856	-2.033729	-1.452493
1	-1.001657	-2.430062	-2.618265
8	-0.208922	1.569714	0.436030
1	-0.536917	1.297074	-0.445559
1	-0.053917	0.725899	0.904399
8	4.336142	2.017937	0.438658
1	4.992874	2.134933	-0.249881
1	3.530255	2.440412	0.078113
8	-0.039718	-0.850662	1.816002
1	-0.994676	-0.985937	1.961814
1	0.192537	-1.476844	1.123212
8	-3.393721	-1.425219	-0.487137
1	-3.593105	-0.508261	-0.739971
1	-2.550477	-1.621549	-0.928857
8	-2.781882	1.819329	1.576155
1	-1.867191	1.830406	1.239919
1	-2.881579	0.911959	1.906998
8	-2.800313	-0.953415	2.132831
1	-3.269928	-1.542662	2.724958
1	-3.068873	-1.215890	1.225471

Cartesian coordinates of : IMZW10_22

AN	X	Y	Z
6	-3.431045	-0.462705	-0.444749
6	-3.479424	0.873607	-0.177825
6	-1.476542	0.313148	-0.849995
1	-4.218280	-1.191356	-0.354459
1	-4.271267	1.511551	0.169052
1	-1.883659	2.313069	-0.372839
1	-0.442757	0.417718	-1.134894
7	-2.171642	-0.810824	-0.875303
7	-2.225624	1.352034	-0.444606
8	-1.733147	-0.508758	2.755039
1	-1.594177	-0.733320	3.676842
1	-1.621412	-1.355239	2.281001
8	-1.216902	-2.734242	1.128327
1	-0.254754	-2.670196	1.081735
1	-1.505261	-2.226482	0.351590
8	1.419076	2.098051	-0.249137
1	1.059621	1.486085	0.419423
1	1.561479	1.515315	-1.017326
8	0.042645	-2.031495	-2.211394
1	-0.023505	-2.846264	-2.711967
1	-0.851068	-1.863736	-1.863184
8	1.962738	0.154565	-2.247257
1	2.649510	-0.207782	-1.664461
1	1.302057	-0.550894	-2.300565
8	-0.765823	3.800620	-0.496906
1	0.043780	3.256442	-0.428556
1	-0.791156	4.288113	0.328129
8	1.464458	-2.172109	0.205207
1	2.347889	-1.794454	0.047230
1	1.028299	-2.156542	-0.665284
8	3.430951	1.208366	1.693492
1	3.085188	1.809970	1.020546
1	2.616966	0.786912	2.007662
8	0.788650	0.148680	1.678515
1	-0.076660	0.012835	2.106201
1	0.933125	-0.662912	1.157304
8	3.797090	-0.729107	-0.278688
1	3.800766	-0.074644	0.447122
1	4.690324	-1.073514	-0.323100

Cartesian coordinates of : IMZW10_23

Atomic number (AN) and Cartesian coordinates

AN	X	Y	Z
6	2.002922	2.191534	1.140683
6	3.075534	1.366585	0.959768
6	1.741874	1.262299	-0.770421

```
  1      1.784598      2.829200      1.979887
  1      3.928689      1.145409      1.574690
  1      3.472638      0.051497     -0.682040
  1      1.362510      0.963864     -1.732947
  7      1.171647      2.126318      0.048282
  7      2.897645      0.790143     -0.268109
  8     -1.765598     -0.992530      2.351223
  1     -2.172661     -0.192911      1.951669
  1     -2.073785     -1.004789      3.258962
  8     -3.001871      1.072479      1.067621
  1     -3.449765      0.600966      0.346024
  1     -2.453607      1.732594      0.607822
  8      0.907691     -0.938081      1.720514
  1      1.274801     -0.062921      1.881431
  1     -0.004412     -0.905196      2.069700
  8     -2.703632     -2.529481      0.152075
  1     -3.261212     -1.872930     -0.296403
  1     -2.499900     -2.114679      1.005908
  8      2.048252     -2.814417     -0.117289
  1      1.315468     -2.623535     -0.716084
  1      1.836909     -2.240005      0.634120
  8     -3.969319     -0.382471     -1.158071
  1     -3.259401      0.020007     -1.699260
  1     -4.783228     -0.237027     -1.642566
  8     -1.449298      2.833069     -0.444600
  1     -0.491069      2.695603     -0.242060
  1     -1.568070      3.779911     -0.529817
  8     -0.372737     -1.313075     -0.888625
  1     -1.150038     -1.809884     -0.568601
  1      0.041966     -0.990967     -0.072978
  8      4.247417     -1.537188     -1.252433
  1      3.489668     -2.053389     -0.912707
  1      4.151208     -1.574099     -2.205602
  8     -1.701927      0.718485     -2.292714
  1     -1.164615      0.068267     -1.802042
  1     -1.645541      1.522950     -1.750108
 ---------------------------------------------------

Cartesian coordinates of : IMZW10_24

 ---------------------------------------------------
 Atomic number (AN) and Cartesian coordinates
  AN          X             Y             Z
 ---------------------------------------------------
  6     -1.303669     -2.308918     -0.047296
  6     -0.156900     -2.478720     -0.767812
  6      0.152321     -0.992902      0.810375
  1     -2.256429     -2.793384     -0.180533
  1      0.087612     -3.110566     -1.601872
  1      1.743236     -1.543461     -0.480541
  1      0.658981     -0.276547      1.434614
  7     -1.101790     -1.382349      0.946619
  7      0.761068     -1.633860     -0.205846
  8     -4.378838     -0.282799     -0.913085
```

1	-4.876342	0.532986	-0.998873
1	-4.169009	-0.329271	0.036902
8	-2.334377	1.950813	0.526746
1	-2.105555	1.417344	-0.245852
1	-2.811310	1.324819	1.094149
8	2.842246	1.049660	-1.935234
1	3.346976	1.784765	-2.286556
1	1.920111	1.375157	-1.878360
8	-3.506678	-0.288840	1.753670
1	-2.631485	-0.728166	1.637678
1	-3.874554	-0.632239	2.568748
8	3.628209	-0.967500	1.916185
1	3.306105	-0.077963	1.667295
1	4.510286	-0.810964	2.257634
8	3.570446	-1.459123	-0.872631
1	3.755496	-1.316312	0.071785
1	3.463055	-0.566394	-1.234977
8	0.203782	2.479058	1.435652
1	-0.731794	2.286760	1.216940
1	0.257415	3.436410	1.449574
8	0.318719	2.014417	-1.483203
1	-0.435568	1.423244	-1.632888
1	0.336080	2.145018	-0.523455
8	-1.869095	0.246171	-1.987805
1	-2.777808	0.073969	-1.665398
1	-1.422489	-0.594279	-1.838334
8	2.800702	1.470013	0.925969
1	2.857792	1.336600	-0.033851
1	1.882820	1.744222	1.079043

Cartesian coordinates of : IMZW10_25

Atomic number (AN) and Cartesian coordinates

AN	X	Y	Z
6	-0.186884	2.786308	0.380399
6	-1.394792	2.919542	-0.237892
6	-1.266124	0.969929	0.748342
1	0.630345	3.486611	0.417203
1	-1.826751	3.715960	-0.815307
1	-3.012309	1.503035	-0.299239
1	-1.560735	-0.009543	1.086237
7	-0.111706	1.561955	1.005170
7	-2.069482	1.756478	0.011235
8	1.277689	0.161314	-1.416312
1	0.871905	0.749120	-0.766692
1	1.671081	-0.532796	-0.857264
8	3.732213	1.522441	-0.510550
1	3.386288	1.351431	0.382343
1	2.988073	1.263735	-1.070657
8	-3.355779	-1.715210	-0.426101
1	-2.988790	-1.856152	0.464014

```
  1     -2.578894     -1.515349     -0.970599
  8      2.412469      0.748670      1.860991
  1      2.550549      0.932289      2.791341
  1      1.485560      1.025341      1.666573
  8      2.523444     -1.655063      0.473023
  1      3.361380     -1.581527     -0.019942
  1      2.534441     -0.889064      1.074797
  8     -4.619551      0.716437     -0.739650
  1     -4.258695     -0.177589     -0.568074
  1     -5.210380      0.881112     -0.002821
  8      4.849520     -1.020294     -0.958119
  1      5.607807     -1.162646     -0.388844
  1      4.600015     -0.090404     -0.798031
  8     -0.082346     -2.661665      0.181735
  1     -0.442752     -2.184420     -0.580653
  1      0.828049     -2.333673      0.262378
  8     -1.111604     -1.129130     -2.092711
  1     -0.943050     -1.728526     -2.821956
  1     -0.273537     -0.644153     -1.967682
  8     -2.052747     -2.217066      2.064472
  1     -2.371734     -3.081222      2.330405
  1     -1.279213     -2.408747      1.498308
 ---------------------------------------------------

Cartesian coordinates of : IMZW10_26

 ---------------------------------------------------
 Atomic number (AN) and Cartesian coordinates
  AN         X             Y             Z
 ---------------------------------------------------
  6      0.186357      2.786396      0.381986
  6      1.393824      2.919844     -0.237125
  6      1.265853      0.969879      0.748507
  1     -0.630832      3.486699      0.419651
  1      1.825390      3.716479     -0.814536
  1      3.011295      1.503400     -0.300146
  1      1.560693     -0.009722      1.085828
  7      0.111618      1.561815      1.006349
  7      2.068691      1.756699      0.011118
  8     -2.412465      0.747352      1.860836
  1     -1.485535      1.024377      1.666916
  1     -2.551219      0.931124      2.791053
  8     -2.523480     -1.656023      0.472561
  1     -3.361061     -1.582110     -0.020938
  1     -2.534648     -0.890159      1.074514
  8      3.356159     -1.714802     -0.425520
  1      2.989135     -1.855948      0.464553
  1      2.579226     -1.515306     -0.970084
  8     -3.731985      1.522806     -0.510075
  1     -3.385657      1.351165      0.382555
  1     -2.987954      1.265089     -1.070733
  8     -1.277257      0.161296     -1.416095
  1     -1.670669     -0.532734     -0.857000
  1     -0.872066      0.749543     -0.766519
```

8	4.618364	0.717268	-0.741731
1	5.209883	0.882953	-0.005687
1	4.258198	-0.176807	-0.569010
8	-4.848605	-1.019964	-0.959869
1	-4.599167	-0.090200	-0.798967
1	-5.606800	-1.162899	-0.390617
8	0.082645	-2.661749	0.181861
1	-0.827831	-2.333893	0.262195
1	0.443289	-2.184377	-0.580326
8	2.052990	-2.217380	2.064755
1	1.279492	-2.408907	1.498493
1	2.372331	-3.081633	2.329950
8	1.112192	-1.128822	-2.092335
1	0.274067	-0.643930	-1.967280
1	0.943600	-1.728379	-2.821436

Cartesian coordinates of : IMZW10_27

Atomic number (AN) and Cartesian coordinates

AN	X	Y	Z
6	-2.162534	-2.462108	-0.852307
6	-3.187546	-1.566151	-0.789749
6	-1.268955	-0.515028	-0.758409
1	-2.213874	-3.535868	-0.907557
1	-4.254872	-1.689289	-0.781256
1	-3.081467	0.569443	-0.660710
1	-0.564106	0.301765	-0.721751
7	-0.958462	-1.798198	-0.838602
7	-2.600772	-0.331415	-0.735063
8	0.137180	-2.287728	1.887331
1	-0.141878	-2.248859	0.960163
1	1.095683	-2.150626	1.833508
8	2.785575	-1.625178	1.169881
1	3.712031	-1.843057	1.280283
1	2.572426	-1.802815	0.227391
8	-1.445820	2.579153	1.050996
1	-1.284050	1.748857	1.524024
1	-0.752033	2.597896	0.369438
8	1.846694	-1.987085	-1.360289
1	0.889332	-2.045425	-1.168969
1	1.940021	-1.103471	-1.759886
8	2.030879	0.639842	-2.335971
1	2.753663	0.944138	-1.752770
1	2.397392	0.684467	-3.221281
8	-3.797243	2.239885	-0.334054
1	-2.976562	2.454375	0.156258
1	-3.728426	2.745691	-1.145567
8	3.929108	1.453298	-0.441839
1	3.325293	1.496291	0.308758
1	4.115391	2.374350	-0.636927
8	1.694879	0.922316	1.428890

```
  1      0.833396      0.780815      1.856199
  1      2.082663      0.030559      1.359380
  8     -0.846187      0.217148      2.553812
  1     -0.547926     -0.700289      2.383765
  1     -0.801112      0.321589      3.505781
  8      0.806412      2.611739     -0.639142
  1      0.936402      2.113040     -1.456120
  1      1.189636      2.016304      0.029377
 ---------------------------------------------------
```

Cartesian coordinates of : IMZW10_28

```
 ---------------------------------------------------
 Atomic number (AN) and Cartesian coordinates
  AN          X             Y             Z
 ---------------------------------------------------
  6     -1.504422     -3.065820      0.366380
  6     -2.747936     -2.509985      0.400100
  6     -1.317451     -1.091260     -0.452200
  1     -1.198184     -4.044576      0.692992
  1     -3.695950     -2.882700      0.741538
  1     -3.346169     -0.552391     -0.250618
  1     -0.934032     -0.184767     -0.891501
  7     -0.608642     -2.173916     -0.175234
  7     -2.609941     -1.254193     -0.125493
  8      1.079301     -0.898000      1.883283
  1      0.624720     -1.452482      1.230724
  1      1.098249     -1.419113      2.688054
  8      3.008451      0.980249      1.041659
  1      3.635028      0.361279      0.623210
  1      2.353488      0.382670      1.436907
  8     -2.254091      2.428647      0.066630
  1     -1.733469      2.003579      0.770720
  1     -1.635139      2.436824     -0.680969
  8      2.020637     -2.321289     -1.140742
  1      2.007775     -2.817299     -1.960851
  1      1.099822     -2.365856     -0.794544
  8      1.750092      0.474523     -1.549730
  1      2.115237      0.740655     -0.692300
  1      1.775819     -0.496821     -1.513730
  8     -4.441536      0.889068     -0.549219
  1     -3.706388      1.493940     -0.315743
  1     -5.043724      0.950579      0.194124
  8      4.416362     -1.069125     -0.268527
  1      4.750553     -0.657989     -1.068244
  1      3.608155     -1.520599     -0.562974
  8      1.320616      3.238621      0.508843
  1      2.025001      2.575219      0.555650
  1      0.868655      3.051296     -0.330088
  8     -0.405643      1.521316      1.983554
  1      0.220766      2.123555      1.543786
  1      0.037058      0.657368      1.954251
  8     -0.163062      2.400203     -1.807812
  1     -0.050646      2.843545     -2.650124
```

```
  1      0.423300      1.619540     -1.844372
 ---------------------------------------------------

Cartesian coordinates of : IMZW10_29

 ---------------------------------------------------
 Atomic number (AN) and Cartesian coordinates
  AN          X             Y             Z
 ---------------------------------------------------
  6     -1.282760     -3.059762      0.362953
  6     -2.490431     -2.616220     -0.086930
  6     -1.108813     -0.935517      0.133724
  1     -0.982471     -4.067812      0.591402
  1     -3.408221     -3.126842     -0.313551
  1     -3.091820     -0.600106     -0.504354
  1     -0.740027      0.077119      0.123337
  7     -0.418228     -2.000214      0.504788
  7     -2.360889     -1.261210     -0.229891
  8      1.553972     -0.374784      1.827541
  1      1.667757      0.207742      1.068566
  1      1.013635     -1.102910      1.467779
  8      4.082083     -0.969973      0.472421
  1      3.488798     -0.990392      1.233467
  1      3.914194     -0.084017      0.108315
  8     -2.313974      2.327624      0.431626
  1     -1.823538      1.933423      1.167555
  1     -1.745599      2.160733     -0.336708
  8      2.065418     -2.335969     -1.007823
  1      1.306363     -2.289570     -0.402988
  1      2.794308     -1.929695     -0.505408
  8      1.511092      0.169286     -2.186520
  1      2.197359      0.675393     -1.720645
  1      1.616339     -0.733296     -1.834109
  8     -4.295214      0.800901     -0.767435
  1     -3.707297      1.382376     -0.247777
  1     -5.030918      0.620529     -0.179714
  8      3.324661      1.567196     -0.523332
  1      2.566956      2.013698     -0.097045
  1      3.911473      2.270181     -0.806463
  8      0.952528      2.634128      0.409418
  1      0.460989      2.251309      1.153391
  1      0.402614      2.424945     -0.362012
  8     -0.587309      1.328046      2.529913
  1      0.119318      0.661168      2.456523
  1     -0.374940      1.828018      3.320417
  8     -0.700116      1.770392     -1.842566
  1     -0.581822      2.449233     -2.509281
  1      0.027278      1.132964     -1.999393
 ---------------------------------------------------

Cartesian coordinates of : IMZW10_30

 ---------------------------------------------------
 Atomic number (AN) and Cartesian coordinates
```

AN	X	Y	Z
6	-1.641669	2.954165	0.114800
6	-2.829288	2.334415	-0.134284
6	-1.194627	0.884225	-0.227328
1	-1.459936	3.992775	0.330686
1	-3.838913	2.699232	-0.176775
1	-3.186425	0.263720	-0.561775
1	-0.685887	-0.058938	-0.345718
7	-0.616782	2.040194	0.054827
7	-2.526058	1.017470	-0.349999
8	1.877347	-0.302600	-2.229677
1	1.864138	0.600588	-1.866373
1	2.580112	-0.746305	-1.722735
8	3.737181	0.933605	0.733775
1	3.029632	0.753594	1.368093
1	3.272637	1.462329	0.064286
8	-2.116708	-2.533190	0.346502
1	-1.420446	-2.346628	-0.302415
1	-1.846473	-2.031393	1.130727
8	1.982980	2.237033	-1.033080
1	2.042829	3.006818	-1.601289
1	1.093000	2.282464	-0.623044
8	1.199756	0.871936	2.072312
1	0.699485	1.401604	1.431028
1	0.515331	0.346687	2.509501
8	-4.242616	-1.221539	-0.828997
1	-3.536269	-1.764985	-0.424140
1	-4.190619	-1.427785	-1.764011
8	3.781139	-1.579854	-0.518440
1	3.028859	-1.941297	-0.026663
1	3.900073	-0.711727	-0.088494
8	1.287275	-1.849486	0.911066
1	1.399555	-0.890405	0.998656
1	0.775810	-1.957484	0.091072
8	-0.102925	-2.107175	-1.624001
1	0.536444	-1.416185	-1.898855
1	0.264935	-2.926896	-1.959605
8	-0.867661	-1.169905	2.592688
1	-0.114256	-1.631474	2.186011
1	-1.016392	-1.629392	3.421735

Cartesian coordinates of : IMZW10_31

Atomic number (AN) and Cartesian coordinates

AN	X	Y	Z
6	1.487620	2.812311	0.052811
6	2.735613	2.304386	-0.150529
6	1.228110	0.775059	-0.569567
1	1.209181	3.801943	0.371837
1	3.715101	2.734368	-0.050659

```
  1      3.275802      0.320318     -0.770600
  1      0.804153     -0.175383     -0.853590
  7      0.542853      1.849535     -0.215389
  7      2.550461      1.010003     -0.553535
  8     -1.630101      1.840670      1.667005
  1     -0.905865      1.914290      1.014784
  1     -1.220858      2.044418      2.510435
  8     -3.897041      1.011473      0.092082
  1     -3.373466      1.253632      0.866434
  1     -3.279357      1.210014     -0.634560
  8      2.517550     -2.433056      0.538604
  1      2.247258     -1.804198      1.230787
  1      1.694322     -2.542555      0.032181
  8     -1.843926      1.446863     -1.748114
  1     -1.884816      2.060987     -2.483475
  1     -1.068152      1.723875     -1.223412
  8     -1.849316     -1.329371     -2.217957
  1     -2.534827     -1.563665     -1.572925
  1     -1.773276     -0.364871     -2.118233
  8      4.381630     -1.134155     -1.003674
  1      4.280209     -1.429067     -1.910190
  1      3.738441     -1.674612     -0.498284
  8     -3.579696     -1.719197      0.013864
  1     -4.381156     -2.211023      0.200225
  1     -3.838030     -0.774481      0.052049
  8     -1.383026     -0.973895      1.614456
  1     -1.530601     -0.011180      1.609342
  1     -2.177468     -1.348555      1.199308
  8      1.353087     -0.655943      2.423202
  1      0.441726     -0.881511      2.185696
  1      1.466929     -1.036243      3.296311
  8     -0.107945     -2.601532     -0.408710
  1     -0.389676     -2.039601      0.329023
  1     -0.566549     -2.189078     -1.162375
 ---------------------------------------------------

Cartesian coordinates of : IMZW64

 ---------------------------------------------------
 Atomic number (AN) and Cartesian coordinates
  AN          X             Y             Z
 ---------------------------------------------------
  6       -0.775383    5.357865   -3.890170
  6       -1.947984    4.809717   -4.332267
  6       -0.597422    3.236730   -3.651175
  1       -0.487170    6.394242   -3.852778
  1       -2.842024    5.248740   -4.735934
  1       -2.525022    2.732423   -4.398332
  1       -0.234583    2.247309   -3.416729
  7        0.071743    4.364184   -3.461879
  7       -1.815757    3.458922   -4.172348
  8        3.687160   -2.837308    3.434720
  1        4.070093   -3.737749    3.340109
  1        4.425010   -2.221076    3.504365
```

8	1.684096	-5.637152	2.128454
1	0.891402	-5.396112	2.628057
1	1.903002	-4.802084	1.668500
8	-0.413376	2.800099	4.644392
1	0.519569	2.833531	4.366400
1	-0.849642	3.503567	4.135159
8	3.351542	6.021382	0.379457
1	3.480096	6.964991	0.468997
1	2.568757	5.934088	-0.217330
8	-0.395816	-3.965891	2.947451
1	-0.804122	-4.046342	2.064548
1	-1.109919	-3.699009	3.546093
8	-4.396144	1.585013	4.592292
1	-4.464464	0.610483	4.549113
1	-3.821308	1.805124	5.343463
8	-0.915144	0.133633	4.005386
1	-0.071030	-0.328787	4.115924
1	-0.793560	1.036081	4.351716
8	2.341745	-3.179166	1.011067
1	2.881876	-2.887477	1.770121
1	2.944494	-3.214196	0.239396
8	1.174360	5.964162	-1.180475
1	0.436786	5.901362	-0.551713
1	0.979175	5.306372	-1.864139
8	-7.164988	0.925515	-0.467960
1	-8.091364	1.166527	-0.473875
1	-6.712751	1.669063	-0.005954
8	-5.194091	3.878826	-1.726153
1	-4.279256	4.145533	-1.556206
1	-5.118320	2.983583	-2.095971
8	0.330088	-1.585030	0.135889
1	0.796557	-0.989213	-0.475045
1	1.033240	-2.161210	0.508817
8	-3.720077	-5.028234	-3.425180
1	-2.923335	-5.532372	-3.132784
1	-4.188728	-5.618487	-4.014903
8	2.000975	2.657434	3.271534
1	2.755945	3.278929	3.287170
1	1.563899	2.894220	2.429489
8	-2.907435	-6.656282	0.027572
1	-3.695811	-6.107034	-0.220225
1	-3.218565	-7.326876	0.632496
8	4.444439	-5.371411	2.860643
1	3.539364	-5.705523	2.911372
1	4.640553	-5.461458	1.909743
8	-3.552489	-0.693957	-1.612167
1	-4.074503	-1.197268	-0.962803
1	-3.177649	0.042502	-1.097185
8	0.862568	-1.321689	-5.461208
1	0.786603	-2.062620	-4.822270
1	1.071549	-1.720334	-6.304599
8	-3.684153	0.410325	1.084676
1	-3.509418	1.283699	1.474311
1	-4.588968	0.174536	1.355738

8	0.295718	-3.226915	-3.611132
1	0.770886	-3.886070	-3.084650
1	-0.385111	-2.905609	-2.988612
8	2.823495	2.756770	-0.968528
1	3.683565	3.119093	-0.687877
1	2.760893	2.971152	-1.923699
8	-1.980664	1.687758	-0.962767
1	-1.744511	1.458454	-0.050735
1	-1.253891	1.282257	-1.473475
8	-2.520863	2.502276	6.452283
1	-2.350919	2.145917	7.322336
1	-1.652443	2.584774	6.029989
8	4.011823	-0.841378	-2.485870
1	3.621018	-0.540499	-1.639669
1	4.855778	-0.364807	-2.570199
8	-2.369140	-2.318614	4.140376
1	-2.325801	-2.105233	3.188007
1	-1.926028	-1.549505	4.522327
8	-5.347674	1.186605	-2.645449
1	-6.170986	1.035910	-2.161774
1	-4.750585	0.499857	-2.299269
8	2.512753	0.092383	-0.440673
1	2.606958	0.067775	0.529302
1	2.601478	1.039418	-0.661133
8	-5.950802	3.037517	0.764074
1	-5.102297	3.005402	1.236554
1	-5.744706	3.504112	-0.074903
8	-2.448083	4.371040	-1.124068
1	-2.081935	4.722720	-1.942300
1	-2.222296	3.413556	-1.124905
8	-0.581441	0.810515	1.336180
1	-0.147191	0.002413	1.017970
1	-0.794167	0.617485	2.272629
8	-4.814446	-5.034547	-0.829099
1	-4.856888	-4.157981	-0.414891
1	-4.558013	-4.878435	-1.751497
8	-0.756247	5.333963	0.807994
1	-1.473106	5.082652	0.193010
1	-0.199221	4.527896	0.828655
8	2.673457	0.150684	2.325211
1	2.445881	1.016229	2.724275
1	2.049702	-0.497027	2.703951
8	-2.239984	-1.774962	1.457998
1	-2.594315	-0.860430	1.360068
1	-1.373910	-1.776267	1.030708
8	4.160507	-3.245182	-1.051636
1	4.894467	-2.881213	-0.525819
1	4.059694	-2.615984	-1.782584
8	-6.257176	-0.512430	1.751518
1	-6.516957	0.218345	2.357670
1	-6.713210	-0.271145	0.928823
8	6.103827	-2.371307	0.702943
1	5.739844	-1.816837	1.430274
1	6.467806	-3.153852	1.118538

8	6.578594	3.773515	1.909020
1	7.313650	4.345344	2.125088
1	6.906319	2.858563	2.030101
8	7.467851	-0.056891	-0.221314
1	7.077823	0.279274	-1.045138
1	7.088393	-0.936389	-0.084545
8	-1.558732	-2.643703	-1.633767
1	-2.270851	-1.984721	-1.752297
1	-0.893489	-2.172052	-1.100386
8	4.694011	-5.759433	0.090156
1	3.978131	-6.392203	-0.053748
1	4.494364	-5.036544	-0.519940
8	-1.794177	4.795680	3.224354
1	-2.120480	5.544604	3.721174
1	-1.409975	5.154579	2.391490
8	5.538305	3.228543	-3.102517
1	5.518428	3.679605	-2.244325
1	4.642937	3.339380	-3.452456
8	-3.543473	1.455811	-4.800129
1	-4.322709	1.350680	-4.238100
1	-3.012085	0.652201	-4.675435
8	0.776444	-5.108312	-1.574306
1	1.357471	-5.803511	-1.230637
1	0.246224	-4.840713	-0.804224
8	7.337637	1.162378	2.121421
1	7.414502	0.767708	1.212845
1	8.163845	0.955355	2.557072
8	-4.648068	-2.482546	0.321812
1	-5.259975	-2.054876	0.933738
1	-3.775219	-2.425821	0.752242
8	6.333275	0.723563	-2.670801
1	6.950318	0.535465	-3.377271
1	6.025066	1.651541	-2.840305
8	-0.128574	0.092055	-2.194281
1	-0.710680	-0.263477	-2.894472
1	0.709933	0.272136	-2.654666
8	2.746476	3.234370	-3.652512
1	1.954706	3.791428	-3.741607
1	2.477886	2.338514	-3.928826
8	2.099717	0.567060	-3.859863
1	2.830401	0.048374	-3.462849
1	1.773618	0.008715	-4.585306
8	-2.234710	-3.204807	-4.958590
1	-1.372019	-3.553201	-4.702304
1	-2.861287	-3.746517	-4.451624
8	-3.608509	2.833626	2.331109
1	-3.022918	3.570100	2.563902
1	-3.844436	2.414159	3.187966
8	-1.193508	-4.517906	0.376164
1	-1.599622	-3.903676	-0.261097
1	-1.764973	-5.306804	0.372152
8	1.131600	-1.744161	3.623567
1	1.950944	-2.188266	3.890815
1	0.586138	-2.467521	3.256975

8	-1.674242	-0.613136	-4.411240
1	-2.048044	-1.516773	-4.511834
1	-0.937842	-0.629119	-5.037956
8	-1.609679	-6.413067	-2.493661
1	-0.773539	-5.956180	-2.307402
1	-1.928725	-6.705823	-1.628858
8	3.935629	4.540712	2.807965
1	3.627499	5.028119	2.031167
1	4.833664	4.263253	2.587377
8	5.171253	4.158542	-0.483357
1	5.759552	3.999325	0.271460
1	4.663013	4.954884	-0.245414
8	-4.899742	-1.107741	4.333135
1	-5.386211	-1.293133	3.526120
1	-4.107728	-1.669856	4.297607
8	0.791835	3.109003	0.842760
1	1.461407	3.038649	0.135944
1	0.326586	2.251152	0.893922
8	-6.854286	1.726171	3.195811
1	-6.752056	2.424005	2.538317
1	-6.157244	1.886606	3.848214
8	2.165045	-7.100545	-0.088780
1	1.859892	-6.756690	0.776642
1	1.900396	-8.018379	-0.124617
8	5.253889	-0.691624	2.637782
1	5.899413	0.029696	2.653241
1	4.369042	-0.287804	2.526406
